# Copper(II)-Catalyzed Three-Component Arylation/Hydroamination Cascade from Allyl Alcohol: Access to 1-Aryl-2-sulfonylamino-propanes

**DOI:** 10.1021/acs.joc.3c01536

**Published:** 2023-09-25

**Authors:** Camilla Loro, Marta Papis, Francesca Foschi, Gianluigi Broggini, Giovanni Poli, Julie Oble

**Affiliations:** † Dipartimento di Scienza e Alta Tecnologia, Università dell’Insubria, via Valleggio 11, 22100 Como, Italy; ‡ Faculté des Sciences et Ingénierie, CNRS, Institut Parisien de Chimie Moléculaire, IPCM, Sorbonne Université, 4 place Jussieu, 75005 Paris, France

## Abstract

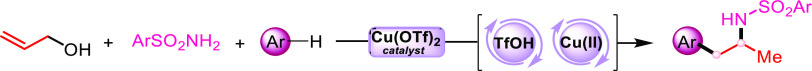

A new straightforward approach to 1-aryl-2-aminopropanes using easily accessible substrates has been developed. Simple allyl alcohol is shown to be an ideal synthetic equivalent of the C3 propane-1,2-diylium bis-cation synthon in three-component cascade reactions with arenes and sulfonamide nucleophiles to regioselectively afford 1-aryl-2-aminopropanes. The reaction is catalyzed by Cu(OTf)_2_ and is expected to involve a Friedel–Crafts-type allylation of the arene, followed by hydroamination.

## Introduction

1-Aryl-2-aminopropanes are widely applied in synthetic chemistry and in the pharmacological field. Also known as amphetamines, they are substances that belong to the psychoanaleptic group, known for their stimulating effects on the sympathetic nervous system as well as for their ability to inhibit various enzymes.^1^

**Scheme 1:**
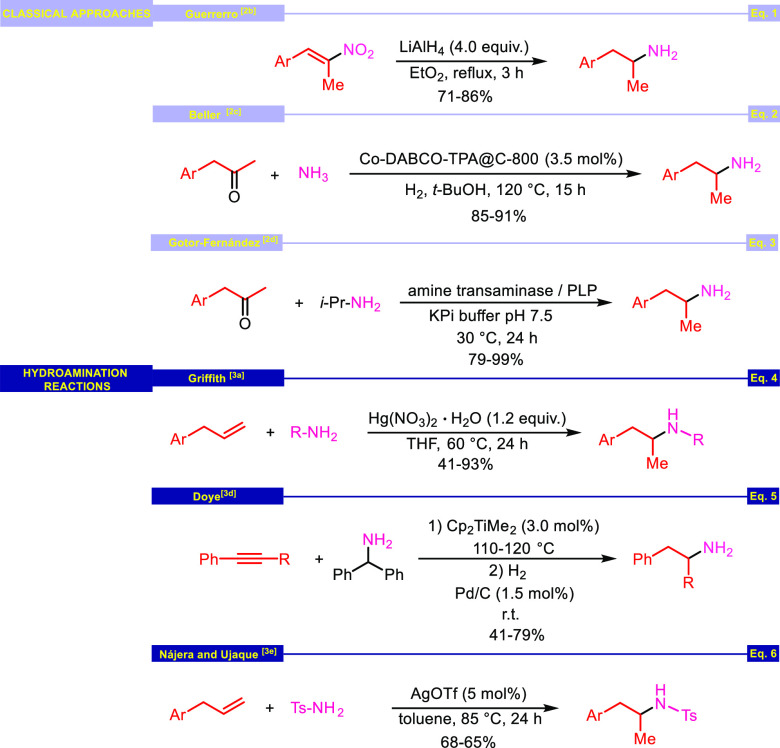
Selected Procedures for the Synthesis of 1-Aryl-2-aminopropanes

Several methods for the synthesis of 1-aryl-2-aminopropanes are reported in the literature, many of which are based on classical reactions of organic chemistry (Scheme 1, eqs 1–3).^2^ Complementary methods reach these compounds by hydroamination of allyl, vinyl, or alkynyl arenes promoted by various catalysts or promoters (Scheme 1, eqs 4–6).^3^

Being interested in transformations involving the concatenated generation of several bonds in a single synthetic operation,^4^ we have recently developed a new copper-promoted reaction, which allows access to 1-aryl-2-aminopropanes starting from *O*-allyl *N*-tosyl carbamates.^5^ This synthetic procedure, although innovative, required the use of a large excess of Cu(OTf)_2_. For this reason, we decided to pursue our studies to further upgrade this synthetic transformation.

**Scheme 2:**
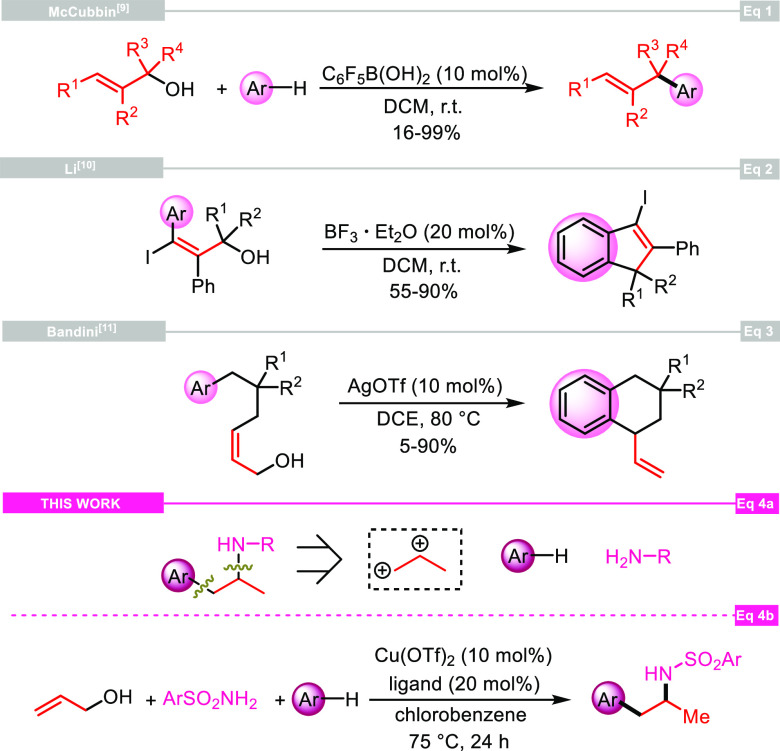
Allylic Alcohols as Variously Substituted C3 Synthons in Friedel–Crafts Reactions

Allyl alcohol derivatives have been used in various protocols as variously substituted electrophilic C3 synthons through the involvement of either the corresponding π-allyl metal complexes (in the presence of catalytic amounts of low-valent transition metals)^6^ or the corresponding allylic cations (in the presence of a protic acid promoter).^7^ Allylic alcohols have also attracted considerable interest in the field of Friedel–Crafts (FC) reactions, enabling the allylation of aromatic or heteroaromatic systems.^8^ A representative example involves the allylation of electron-rich (hetero)arenes with allylic alcohols, in which the carbocationic species is generated by catalytic amounts of pentafluorophenylboronic acid (Scheme 2, eq 1).^9^ In 2008, an innovative way to synthesize 3-iodoindenes through an intramolecular FC reaction of 3-iodo-3-arylprop-2-en-1-ols in the presence of catalytic amounts of F_3_B·OEt_2_ was reported (Scheme 2, eq 2).^10^ One year later, Bandini and co-workers obtained 1-vinyl-tetrahydronaphthalenes by the cyclization of 6-arylhex-2-en-1-ol motifs with AgOTf (Scheme 2, eq 3).^11^ As for our contribution, we envisioned the use of the allyl alcohol motif as a synthetic equivalent of the C3 propane-1,2-diylium bis-cation synthon in cascade reactions with aryl derivatives and nitrogen-based nucleophiles to regioselectively reach 1-aryl-2-aminopropanes (Scheme 2, eq 4a). Such a three-component process could be successfully attained using sulfonamides and electron-rich arenes in the presence of catalytic Cu(OTf)_2_ under very mild conditions (Scheme 2, eq 4b).

## Results and Discussion

Our investigation began with the evaluation of the reaction conditions previously adopted with the *O*-allyl carbamates.^5^ Accordingly, reacting allyl alcohol with 2.0 equiv of tosylamide in the presence of 4.0 equiv. of Cu(OTf)_2_ in mesitylene as the solvent at 130 °C for 3.0 h gave a mixture of 1,2-arylation/hydroamination **1a** and 1,2-diarylation products **2** in 34 and 23% yields, respectively (Table 1, entry 1). Using chlorobenzene as the solvent and 5 equiv of mesitylene gave selectively the three-component C–C/C–N coupling product **1a** in 63% isolated yield (entry 2). Lowering the amount of Cu(OTf)_2_ to 1.0 equiv was nearly as effective (entry 3), while using 10 mol % Cu(OTf)_2_ gave a lower yield for **1a** (entry 4). To improve this result, this copper-catalyzed reaction was studied in the presence of different ligands,^12^ and to our delight, the use of the diphosphine ligand xantphos selectively led to **1a** in 78% yield after 24 h at 100 °C (entry 5). By lowering the reaction temperature to 75 °C, the yield of **1a** further increased to 81% (entry 6).^13^ However, further lowering the reaction temperature to 50 °C only returned the starting substrate back (entry 7), while dropping the amount of mesitylene to 2.0 equiv lowered the yield of **1a** to 35%. Carrying out the coupling in the presence of 5 mol % TfOH, without Cu(OTf)_2_, gave **1a** and **2** in 71% and 12% isolated yields, respectively, which suggests the *in situ* generation of this acid in the reaction medium (entry 8). Finally, an additional experiment using 20 mol % TfOH at 75 °C afforded only traces of **1a** with a lot of degradation (entry 9).

**Table 1:**
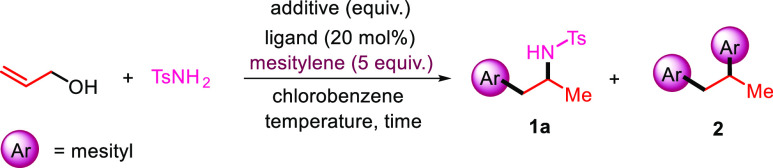
Optimization of the Reaction Conditions^a^

entry	additive (equiv)	ligand (20 mol %)	time (h)	temp. (°C)	yield^b^ (%)
1^c^	Cu(OTf)_2_ (4.0)		3	130	**1a** (34) + **2** (23)
2	Cu(OTf)_2_ (4.0)		4	130	**1a** (63)
3	Cu(OTf)_2_ (1.0)		6	130	**1a** (60)
4	Cu(OTf)_2_ (0.1)		6	130	**1a** (49)
5	Cu(OTf)_2_ (0.1)	xantphos	24	100	**1a** (78)
6^d^	Cu(OTf)_2_ (0.1)	xantphos	24	75	**1a** (81)
7	Cu(OTf)_2_ (0.1)	xantphos	24	50	S.M.
8	TfOH (0.05)		4	130	**1a** (71) + **2** (12)
9	TfOH (0.2)		24	75	**1a** (trace) + degr. products

a Reaction conditions: allyl alcohol (1.0 equiv), tosylamide (2.0 equiv), mesitylene (5.0 equiv), and chlorobenzene (0.25 M) at 75 °C in an oil bath for 24 h.

b Isolated yields.

c Reaction performed in mesitylene as the solvent (0.25 M).

d Reaction performed with 2.0 equiv of mesitylene gave compound **1a** with 35% yield.

**Scheme 3:**
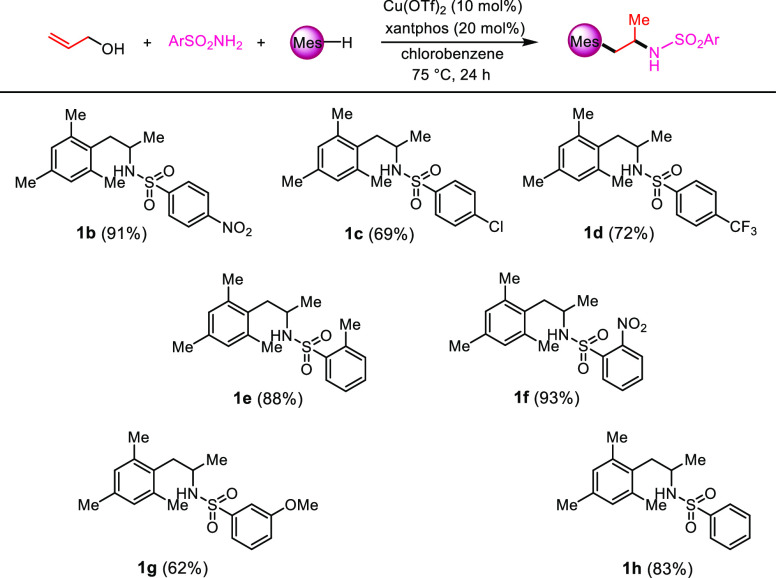
Synthesis of 1-Mesityl-2-sulfonylamino-propanes **1b**–**h**, Reaction conditions: allyl alcohol (1.0 equiv), sulfonamide (2.0 equiv), mesitylene (5.0 equiv), Cu(OTf)_2_ (10 mol %), xantphos (20 mol %), and chlorobenzene as the solvent (0.25 M), 75 °C in an oil bath for 24 h. Isolated yields.

With these optimized conditions in hand, we then proceeded to test this three-component reaction with other sulfonamides (Scheme 3). All of the aryl sulfonamides tested, which incorporated electron-donating and -withdrawing groups on the phenyl ring, gave the expected corresponding products (**1b**–**h**) in good to excellent yields.

**Scheme 4:**
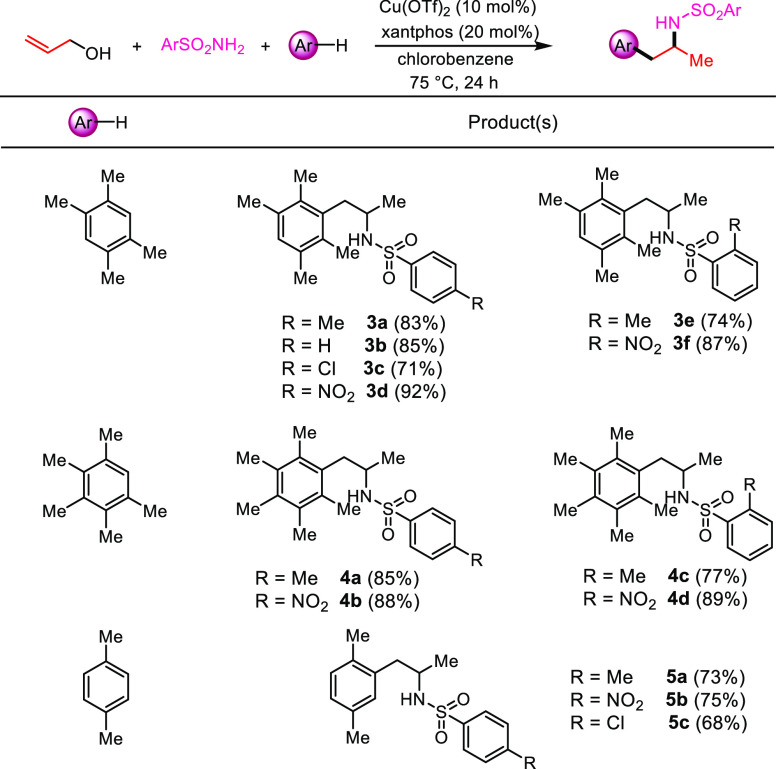
Arylation/Hydroamination with Different Hydrocarbons, Reaction conditions: allyl alcohol (1.0 equiv), sulfonamide (2.0 equiv), hydrocarbons (5.0 equiv), Cu(OTf)_2_ (10 mol %), xantphos (20 mol %), and chlorobenzene as the solvent (0.25 M), 75 °C in an oil bath for 24 h. Isolated yields.

We then explored the substrate scope by using a series of different electron-rich aromatic hydrocarbons and variously substituted aromatic *N*-sulfonamides (Scheme 4). Gratifyingly, durene, 1,2,3,4,5-pentamethylbenzene, and *p*-xylene gave the expected three-component coupling products **3a**–**f**, **4a**–**d**, and **5a**–**c** in good to excellent yields, irrespectively of the steric hindrance of the arene and the electron-donating or -withdrawing character of the substituents of the aromatic ring of the sulfonamide partners.

**Scheme 5:**
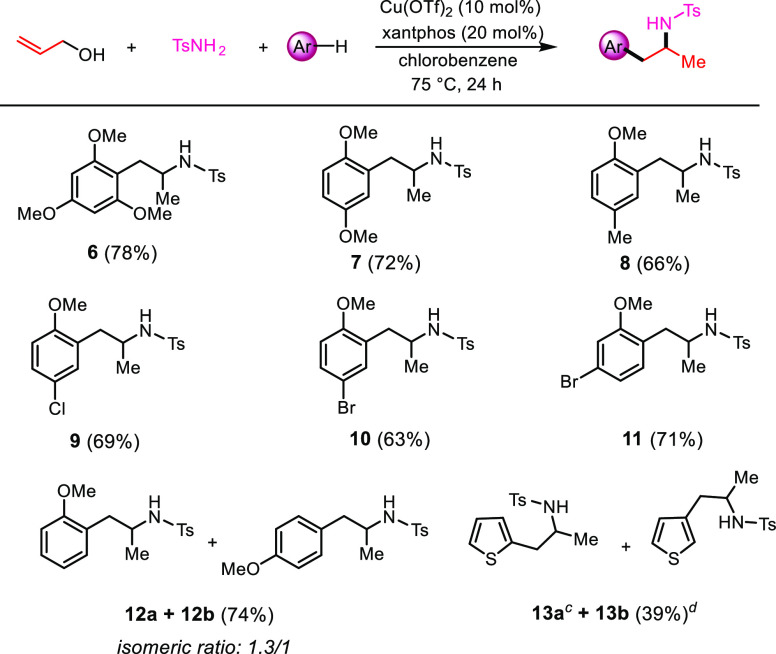
Arylation/Hydroamination with Different Arenes, **13a** only observed by NMR and not isolated pure. **13b** isolated with 39% yield. Reaction conditions: allyl alcohol (1.0 equiv), tosylamide (2.0 equiv), arene (5.0 equiv), Cu(OTf)_2_ (10 mol %), xantphos (20 mol %), and chlorobenzene as the solvent (0.25 M), 75 °C in an oil bath for 24 h. Isolated yields.

The scope of arene coupling was explored, keeping tosylamide as the nitrogen nucleophile and the promoting system [Cu(OTf)_2_/xantphos] in chlorobenzene at 75 °C (Scheme 5). Reacting allyl alcohol and tosylamide with six different arenes bearing electron donor or acceptor heteroatom-based substituents gave the corresponding *N*-tosyl 1-aryl-2-aminopropanes **6**–**11** in good yields. Worthy of note, this approach is complementary to our previously studied one that used *O*-allyl carbamates as the starting bis-cationic C3 synthetic equivalents.^5^ Indeed, in that case, strongly activated arenes such as 1,4-dimethoxybenzene and 1,3,5-trimethoxybenzene selectively led to 1,2-diarylation products rather than the arylation/hydroamination products **6**–**7**, whereas the reaction carried on with anisole gave a mixture of the two possible regioisomers **12a**/**12b**.

Concerning the use of heteroarenes, furan, indole, and *N-*methylindole furnished only a mixture of degradation products. On the other hand, thiophene afforded the two possible products 1-(2-thienyl)-2-tosylaminopropane **13a** and 1-(3-thienyl)-2-tosylaminopropane **13b**. Only isomer **13b**, substituted at the C3 position of the thiophene, was isolated in pure form, whereas isomer **13a** was only observed in the crude NMR spectrum.

**Scheme 6:**
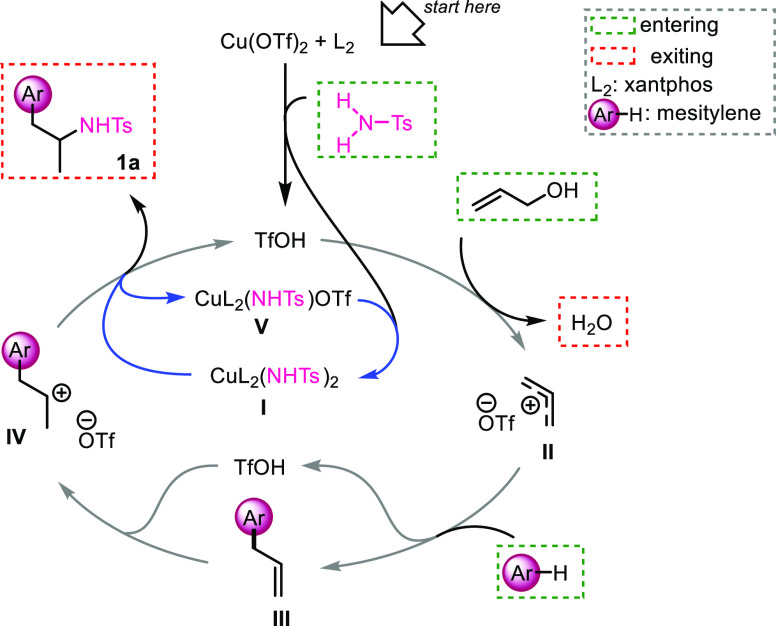
Proposed Mechanism for Arylation/Hydroamination of Allyl Alcohol

For the present coupling reaction, we propose the following mechanism (Scheme 6). First, we postulate that the interaction between tosylamide and Cu(OTf)_2_ in the presence of the bidentate ligand xantphos generates TfOH and the bis-amido Cu(II) complex CuL_2_(NHTs)_2_ (**I**).^14^ The following protonation of allyl alcohol generates allyl carbenium ion **II** accompanied by water release. Subsequent FC allylation of the arene gives the allylated arene **III**, and its subsequent Markovnikov protonation by TfOH generates the new carbenium ion **IV**. At this stage, ligand exchange between a sulfonylamino ligand of **I** and triflate anion generates the final product **1a** and the monoamido Cu(II) complex **V**. Finally, the interaction between tosylamide and **V** regenerates **I**.^15^ In this mechanism, it is possible to distinguish the double TfOH catalytic cycle (arrows in gray) and the interconnected Cu(II) cycle (arrows in blue).

**Scheme 7:**
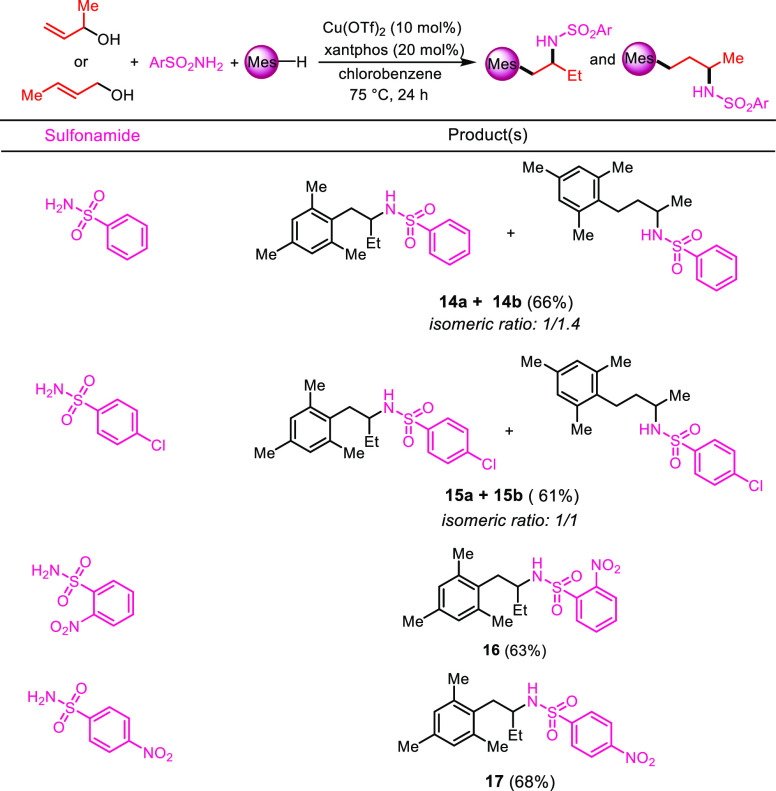
Variation on the Nature of the Alcohol, Reaction conditions: allyl alcohol (1.0 equiv), sulfonamide (2.0 equiv), mesitylene (5.0 equiv), Cu(OTf)_2_ (10 mol %), xantphos (20 mol %), and chlorobenzene as the solvent (0.25 M), 75 °C in an oil bath for 24 h. Isolated yields.

Finally, the three-component coupling has been tested using substituted allylic alcohols (Scheme 7).^16^ On the one hand, reacting crotyl alcohol with mesitylene and benzenesulfonamide or 4-chlorobenzenesulfonamide under the previously optimized conditions gave a 1:1.4 and 1:1 mixture of 3-arylsulfonylamino-4-mesitylbutanes and 2-arylsulfonylamino-4-mesitylbutanes **14a**/**14b** or **15a**/**15b** in 66 and 61% isolated yields, respectively. On the other hand, using 2-nitrobenzenesulfonamide or 4-nitrobenzenesulfonamide as the nitrogen nucleophile gave exclusively the 3-arylsulfonylamino-4-mesitylbutanes **16** and **17** in 63 and 68% yields, respectively. Repeating the same four couplings as above using 3-buten-2-ol instead of crotyl alcohol gave precisely the same results. Thus, the two isomeric allylic alcohols can act in this three-component coupling as butane-1,2-diylium or butane-1,3-diylium C4 synthons. Conversely, treatment of α,α- and γ,γ-dimethyl-substituted allyl alcohols afforded only diarylated derivatives with indane structures.^5,17^

**Scheme 8:**
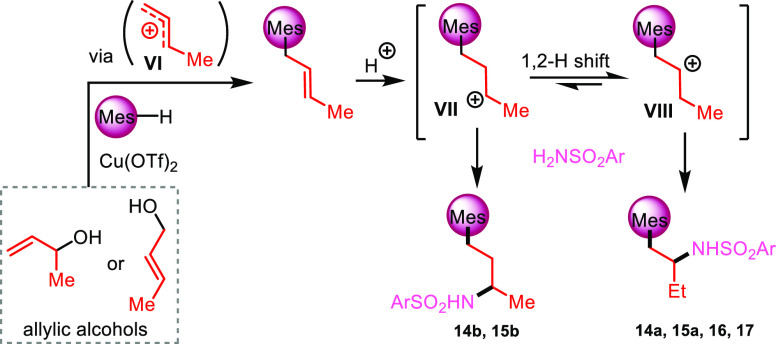
Key Intermediates for Arylation/Hydroamination of Butenol Substrates

The above outcome can be interpreted as follows. Protonation of crotyl alcohol or 3-buten-2-ol generates the common allylic carbenium ion **VI** that is intercepted by the arene to give a crotylated arene. Further protonation of this latter at position 2 or 3 of the chain generates the transient carbenium ions **VII** and **VIII**, which can in turn be trapped by the sulfonamides to give the two regioisomeric final products (Scheme 8).^18^

## Conclusions

In conclusion, we have shown that simple allylic alcohols are ideal C3 (or higher) bis-cationic alkane-1,2-diylium synthons in [FC allylation/hydroamination] cascades. This copper-catalyzed three-component reaction discloses a novel, straightforward, and general preparation of the pharmacologically relevant class of 1-aryl-2-aminopropanes. Future studies will be addressed to test new nucleophiles and intramolecular variants.

## Experimental Section

### General Information

All available chemicals and solvents were purchased from commercial sources and were used without any further purification. Thin-layer chromatography (TLC) was performed using 0.25 mm silica gel precoated plates Si 60-F254 (Merck, Darmstadt, Germany) visualized by UV-254 light and cerium ammonium molybdate (CAM) staining. Purification by flash column chromatography (FCC) was conducted by using silica gel Si 60, 230–400 mesh, 0.040–0.063 mm (Merck). Melting points were determined on a Stuart Scientific SMP3 and are corrected. ^1^H and ^13^C NMR spectra were recorded on a Bruker Avance 400 (400 and 101 MHz, respectively); chemical shifts are indicated in parts per million downfield from SiMe_4_, using the residual proton (CHCl_3_ = 7.27 ppm) and carbon (CDCl_3_ = 77.0 ppm) solvent resonances as an internal reference. Coupling constant values *J* are given in Hz. High-resolution mass spectra (HRMS) were recorded using a mass spectrometer MicroTOF from Bruker with an electron spray ion source (ESI) and a TOF detector or using a mass spectrometer from Thermo Fisher Scientific with an electron spray ion source (ESI) and a LTQ Orbitrap as a detector. FTIR spectra were recorded on a Tensor 27 (ATR Diamond) Bruker infrared spectrophotometer and are reported in frequency of absorption (cm^–1^).

### Safety Note

TfOH is a strong protic acid and corrosive; therefore, it requires careful handling. All reactions should be carried out with safety precautions in a ventilated hood using protective clothing.

#### General Procedure for the Synthesis of Arylated/Hydroaminated Products with Allyl Alcohol

In a sealed tube, after 15 min, the allyl alcohol (1.0 mmol, 58 mg), hydrocarbons (5.0 mmol), and sulfonamide (2.0 mmol) were added to a solution of Cu(OTf)_2_ (10 mol %, 36.2 mg) and xantphos (20 mol %, 11.6 mg) in chlorobenzene (0.25 M). The resulted solution was magnetically stirred and heated at 75 °C in an oil bath for 24 h. The reaction mixture was washed with brine (3 × 5 mL) and the organic layer was extracted with AcOEt (2 × 5 mL), dried over MgSO_4_, and filtered. The solvent was evaporated under reduced pressure. The residue was purified by FCC. Starting from the aromatic source and appropriate sulfonamide, yield, physical, spectroscopic, and analytical data of compounds **1a**–**f**, **3a**–**f**, **4a**–**d**, **5a**–**b**, and **6**–**11** are as follows.

##### 1-(2,4,6-Trimethylphenyl)-2-tosylamino-propane (**1a**)

Mesitylene (0.69 mL); tosylamide (342.4 mg); FCC–AcOEt/hexane (1:1). **1a** (268.2 mg, 81%); light brown oil. ^1^H NMR (CDCl_3_, 400 MHz) δ 7.58 (d, 2H, *J* = 8.3 Hz), 7.19 (d, 2H, *J* = 7.9 Hz), 6.74 (s, 2H), 4.43 (d, 1H, *J* = 7.2 Hz), 3.49–3.42 (m, 1H), 2.79 (dd, 1H, *J* = 14.0, 7.2 Hz,), 2.64 (dd, 1H, *J* = 14.0, 8.1 Hz), 2.41 (s, 3H), 2.23 (s, 3H), 2.14 (s, 6H), 1.14 (d, 3H, *J* = 6.5 Hz); ^13^C{^1^H} NMR (CDCl_3_, 101 MHz) δ 143.1, 137.5, 136.6, 135.8, 131.5, 129.5, 129.2, 127.0, 49.9, 36.9, 21.5, 21.4, 20.8, 20.3. The characterization of product **1a** is consistent with that reported in the literature.^5^

##### 1-(2,4,6-Trimethylphenyl)-2-(*p*-nosylamino)-propane (**1b**)

Mesitylene (0.69 mL); *p*-nosylamide (404.4 mg); FCC–AcOEt/hexane (1:4), R_f_: 0.29. **1b** (329.5 mg, 91%); light brown oil. ^1^H NMR (CDCl_3_, 400 MHz) δ 8.12 (d, 2H, *J* = 8.7 Hz), 7.69 (d, 2H, *J* = 8.7 Hz), 6.64 (s, 2H), 4.49 (d, 1H, *J* = 7.8 Hz), 3.62–3.55 (m, 1H), 2.73 (dd, 1H, *J* = 14.2, 9.2 Hz), 2.65 (dd, 1H, *J* = 14.4, 5.8 Hz), 2.19 (s, 3H), 2.13 (s, 6H), 1.32 (d, 3H, *J* = 6.4 Hz); ^13^C{^1^H} NMR (CDCl_3_, 101 MHz) δ 149.5, 146.0, 136.4, 136.2, 131.1, 129.3, 127.7, 123.8, 50.9, 36.5, 23.1, 20.6, 20.2; IR ν_max_ 2918, 1342, 1158 cm^–1^. HRMS(ESI): *m*/*z* calc. for C_18_H_21_N_2_O_4_S [M – H]^−^: 361.1228; found: 361.1216.

##### 1-(2,4,6-Trimethylphenyl)-2-(4-chlorobenzenesulfonamido)-propane (**1c**)

Mesitylene (0.69 mL); 4-chlorobenzenesulfonamide (383.3 mg); FCC–AcOEt/hexane (3:7), R_f_: 0.33. **1c** (242.3 mg, 69%); light brown oil. ^1^H NMR (CDCl_3_, 400 MHz) δ 7.53 (d, 2H, *J* = 8.4 Hz), 7.30 (d, 2H, *J* = 8.4 Hz), 6.71 (s, 2H), 4.49 (d, 1H, *J* = 7.3 Hz), 3.53–3.44 (m, 1H), 2.76 (dd, 1H, *J* = 14.1, 8.2 Hz), 2.64 (dd, 1H, *J* = 14.2, 6.9 Hz), 2.25 (s, 3H), 2.14 (s, 6H), 1.23 (d, 3H, *J* = 6.4 Hz); ^13^C{^1^H} NMR (CDCl_3_, 101 MHz) δ 138.8, 138.7, 136.3, 136.1, 131.2, 129.3, 128.9, 128.2, 50.3, 36.6, 22.4, 20.8, 20.3; IR ν_max_ 2923, 1321, 1163 cm^–1^. HRMS(ESI): *m*/*z* calc. for C_18_H_21_ClNO_2_S [M – H]^−^: 350.0987; found: 350.0977.

##### 1-(2,4,6-Trimethylphenyl)-2-(4-trifluoromethylbenzenesulfonamido)-propane (**1d**)

Mesitylene (0.69 mL); 4-(trifluoromethyl)benzenesulfonamide (450.4 mg); FCC–AcOEt/hexane (1:9), R_f_: 0.31. **1d** (277.3 mg, 72%); colorless oil. ^1^H NMR (CDCl_3_, 400 MHz) δ 7.69 (d, 2H, *J* = 8.2 Hz), 7.58 (d, 2H, *J* = 8.3 Hz), 6.68 (s, 2H), 4.56 (d, 1H, *J* = 7.4 Hz), 3.55–3.48 (m, 1H), 2.75 (dd, 1H, *J* = 14.2, 8.6 Hz), 2.65 (dd, 1H, *J* = 14.2, 6.6 Hz), 2.23 (s, 3H), 2.12 (s, 6H), 1.27 (d, 3H, *J* = 6.0 Hz); ^13^C{^1^H} NMR (CDCl_3_, 101 MHz) δ 143.8, 136.3, 136.1, 133.9 (q, *J*C–F = 33.0 Hz), 131.1, 129.3, 127.2, 125.8 (q, *J*C–F = 3.7 Hz), 124.7 (q, *J*C–F = 253.6 Hz), 50.4, 36.5, 22.6, 20.6, 20.2; IR ν_max_ 2922, 1339, 1125, 1018 cm^–1^. HRMS(ESI): *m*/*z* calc. for C_19_H_21_F_3_NO_2_S [M – H]^−^: 384.1251; found: 384.1240.

##### 1-(2,4,6-Trimethylphenyl)-2-(2-methylbenzenesulfonamido)-propane (**1e**)

Mesitylene (0.69 mL); 2-methylbenzenesulfonamide (342.4 mg); FCC–AcOEt/hexane (1:4), R_f_: 0.40. **1e** (291.4 mg, 88%); light brown oil. ^1^H NMR (CDCl_3_, 400 MHz) δ 7.85 (d, 1H, *J* = 7.8 Hz), 7.33 (t, 1H, *J* = 7.4 Hz), 7.17 (t, 1H, *J* = 6.6 Hz), 7.10 (d, 1H, *J* = 7.6 Hz), 6.64 (s, 2H), 4.36 (d, 1H, *J* = 7.1 Hz), 3.36–3.29 (m, 1H), 2.69 (dd, 1H, *J* = 14.0, 7.4 Hz), 2.56 (dd, 1H, *J* = 14.0, 8.3 Hz), 2.29 (s, 3H), 2.14 (s, 3H), 2.01 (s, 6H), 1.08 (d, 3H, *J* = 6.4 Hz); ^13^C{^1^H} NMR (CDCl_3_, 101 MHz) δ 137.9, 137.2, 136.6, 135.9, 132.6, 132.5, 131.2, 129.6, 129.3, 125.9, 49.7, 36.7, 21.8, 20.8, 20.2, 20.0; IR ν_max_ 2917, 1299, 1157 cm^–1^. HRMS(ESI): *m*/*z* calc. for C_19_H_24_NO_2_S [M – H]^−^: 330.1533; found: 330.1532.

##### 1-(2,4,6-Trimethylphenyl)-2-(*o*-nosylamino)-propane (**1f**)

Mesitylene (0.69 mL), *o*-nosylamide (404.4 mg); FCC–AcOEt/hexane (1:4). **1f** (336.8 mg, 93%); light brown oil. ^1^H NMR (CDCl_3_, 400 MHz) δ 7.98 (d, 1H, *J* = 7.4 Hz), 7.79 (d, 1H, *J* = 7.6 Hz), 7.68–7.61 (m, 2H), 6.62 (s, 2H), 5.31 (t, 1H, *J* = 3.4 Hz), 3.85–3.78 (m, 1H), 2.85 (dd, 1H, *J* = 14.2, 8.0 Hz), 2.72 (dd, 1H, *J* = 14.2, 7.5 Hz) 2.19 (s, 6H), 2.16 (s, 3H), 1.26 (d, 3H, *J* = 7.5 Hz); ^13^C{^1^H} NMR (CDCl_3_, 101 MHz) δ 136.5, 136.4, 135.7, 134.8, 132.8, 131.1, 130.4, 129.2, 125.44, 125.43, 51.2, 36.6, 22.4, 20.7, 20.3. The characterization of product **1f** is consistent with that reported in the literature.^5^

##### 1-(2,4,6-Trimethylphenyl)-2-(3-methoxybenzenesulfonamido)-propane (**1g**)

Mesitylene (0.69 mL); 3-methoxybenzenesulfonamide (374.4 mg); FCC–AcOEt/hexane (2:3), R_f_: 0.37. **1g** (215.2 mg, 62%); yellow oil. ^1^H NMR (CDCl_3_, 400 MHz) δ 7.31–7.23 (m, 2H), 7.19 (s, 1H), 6.99 (d, 1H, *J* = 7.3 Hz), 6.67 (s, 2H), 4.81 (d, 1H, *J* = 6.9 Hz), 3.75 (s, 3H), 3.46–3.38 (m, 1H), 2.78 (dd, 1H, *J* = 13.9, 7.0 Hz), 2.59 (dd, 1H, *J* = 13.9, 8.3 Hz), 2.16 (s, 3H), 2.09 (s, 6H), 1.08 (d, 3H, *J* = 6.5 Hz); ^13^C{^1^H} NMR (CDCl_3_, 101 MHz) δ 159.8, 141.6, 136.5, 135.8, 131.3, 129.9, 129.3, 119.2, 118.9, 111.4, 55.5, 50.0, 36.8, 21.6, 20.8, 20.3; IR ν_max_ 2968, 1309, 1155 cm^–1^. HRMS(ESI): *m*/*z* calc. for C_19_H_24_NO_3_S [M – H]^−^: 346.1482; found: 346.1468.

##### 1-(2,4,6-Trimethylphenyl)-2-(benzenesulfonamido)-propane (**1h**)

Mesitylene (0.69 mL); benzenesulfonamide (314.4 mg); FCC–AcOEt/hexane (1.5:8.5), R_f_: 0.36. **1h** (263.2 mg, 83%); colorless oil. ^1^H NMR (CDCl_3_, 400 MHz) δ 7.68 (d, 2H, *J* = 7.5 Hz), 7.52 (t, 1H, *J* = 7.3 Hz), 7.40 (t, 2H, *J* = 7.7 Hz), 6.74 (s, 2H), 4.42 (d, 1H, *J* = 6.8 Hz), 3.51–3.44 (m, 1H), 2.79 (dd, 1H, *J* = 13.9, 7.3 Hz), 2.64 (dd, 1H, *J* = 13.9, 7.9 Hz), 2.23 (s, 3H), 2.14 (s, 6H), 1.16 (d, 3H, *J* = 6.5 Hz); ^13^C{^1^H} NMR (CDCl_3_, 101 MHz) δ 140.3, 136.5, 135.9, 132.3, 131.2, 129.3, 128.8, 126.9, 49.9, 36.8, 21.8, 20.8, 20.2; IR ν_max_ 2936, 1326, 1158 cm^–1^. HRMS(ESI): *m*/*z* calc. for C_18_H_22_NO_2_S [M – H]^−^ HRMS(ESI): 316.1377; found: 316.1376.

##### 1-(2,3,5,6-Tetramethylphenyl)-2-tosylamino-propane (**3a**)

1,2,4,5-Tetramethylbenzene (671.1 mg), tosylamide (342.4 mg); FCC–AcOEt/hexane (4:1). **3a** (286.5 mg, 83%); light brown oil. ^1^H NMR (CDCl_3_, 400 MHz) δ 7.49 (d, 2H, *J* = 8.3 Hz), 7.15 (d, 2H, *J* = 8.2 Hz), 6.82 (s, 1H), 4.51 (d, 1H, *J* = 6.7 Hz), 3.45–3.38 (m, 1H), 2.90 (dd, 1H, *J* = 14.3, 7.8 Hz), 2.77 (dd, 1H, *J* = 14.3, 7.5 Hz), 2.41 (s, 3H), 2.16 (s, 6H), 2.04 (s, 6H), 1.19 (d, 3H, *J* = 6.5 Hz); ^13^C{^1^H} NMR (CDCl_3_, 101 MHz) δ 142.9, 137.2, 134.1, 133.9, 132.5, 130.2, 129.3, 126.9, 50.3, 37.2, 21.9, 21.5, 20.7, 16.1. The characterization of product **3a** is consistent with that reported in the literature.^5^

##### 1-(2,3,5,6-Tetramethylphenyl)-2-(benzenesulfonamido)-propane (**3b**)

1,2,4,5-Tetramethylbenzene (671.1 mg), benzenesulfonamide (314.4 mg); FCC–DCM/MeOH (9.9:0.1), R_f_: 0.35. **3b** (281.5 mg, 85%); colorless oil. ^1^H NMR (CDCl_3_, 400 MHz) δ 7.60 (d, 2H, *J* = 8.0 Hz), 7.49 (t, 1H, *J* = 7.4 Hz), 7.37 (t, 2H, *J* = 7.9 Hz), 6.82 (s, 1H), 4.44 (d, 1H, *J* = 6.7 Hz), 3.45–3.38 (m, 1H), 2.90 (dd, 1H, *J* = 14.3, 7.8 Hz), 2.77 (dd, 1H, *J* = 14.3, 7.5 Hz), 2.16 (s, 6H), 2.03 (s, 6H), 1.19 (d, 3H, *J* = 6.5 Hz); ^13^C{^1^H} NMR (CDCl_3_, 101 MHz) δ 140.1, 134.0, 133.9, 132.5, 132.2, 130.4, 128.8, 126.9, 50.3, 37.2, 21.9, 20.7, 16.1; IR ν_max_ 2920, 1379, 1135 cm^–1^. HRMS(ESI): *m*/*z* calc. for C_19_H_25_NO_2_S [M – H]^−^: 330.1533; found: 330.1531.

##### 1-(2,3,5,6-Tetramethylphenyl)-2-(4-chlorobenzenesulfonamido)-propane (**3c**)

1,2,4,5-Tetramethylbenzene (671.1 mg), 4-chlorobenzenesulfonamide (383.3 mg); FCC–AcOEt/hexane (1:4), R_f_: 0.34. **3c** (259.2 mg, 71%); light brown oil. ^1^H NMR (CDCl_3_, 400 MHz) δ 7.41 (d, 2H, *J* = 8.5 Hz), 7.25 (d, 2H, *J* = 8.6 Hz), 6.82 (s, 1H), 4.32 (d, 1H, *J* = 7.2 Hz), 3.46–3.39 (m, 1H), 2.86 (dd, 1H, *J* = 14.5, 8.9 Hz), 2.75 (dd, 1H, *J* = 14.5, 6.2 Hz), 2.15 (s, 6H), 2.02 (s, 6H), 1.29 (d, 3H, *J* = 6.4 Hz); ^13^C{^1^H} NMR (CDCl_3_, 101 MHz) δ 138.6, 138.5, 134.1, 133.8, 132.3, 130.3, 128.8, 128.1, 50.7, 36.9, 22.7, 20.6, 16.1; IR ν_max_ 2917, 1381, 1135 cm^–1^. HRMS(ESI): *m*/*z* calc. for C_19_H_23_ClNO_2_S [M – H]^−^: 364.1144; found: 364.1129.

##### 1-(2,3,5,6-Tetramethylphenyl)-2-(*p*-nosylamino)-propane (**3d**)

1,2,4,5-Tetramethylbenzene (671.1 mg), 4-nitrobenzenesulfonamide (404.4 mg); FCC–AcOEt/hexane (1:4), R_f_: 0.27. **3d** (346.1 mg, 92%); light brown oil. ^1^H NMR (CDCl_3_, 400 MHz) δ 8.08 (d, 2H, *J* = 8.7 Hz), 7.59 (d, 2H, *J* = 8.7 Hz), 6.73 (s, 1H), 4.53 (d, 1H, *J* = 7.8 Hz), 3.58–3.49 (m, 1H), 2.85 (dd, 1H, *J* = 14.5, 9.6 Hz), 2.74 (dd, 1H, *J* = 14.8, 5.5 Hz), 2.09 (s, 6H), 2.02 (s, 6H), 1.37 (d, 3H, *J* = 6.4 Hz); ^13^C{^1^H} NMR (CDCl_3_, 101 MHz) δ 149.5, 145.8, 134.1, 133.9, 132.2, 130.3, 127.5, 123.6, 51.4, 36.9, 23.3, 20.5, 16.1; IR ν_max_ 2920, 1345, 1160 cm^–1^. HRMS(ESI): *m*/*z* calc. for C_19_H_23_N_2_O_4_S [M – H]^−^: 375.1384; found: 375.1368.

##### 1-(2,3,5,6-Tetramethylphenyl)-2-(2-methylbenzenesulfonamido)-propane (**3e**)

1,2,4,5-Tetramethylbenzene (671.1 mg), 2-methylbenzenesulfonamide (342.4 mg); FCC–AcOEt/hexane (1:4), R_f_: 0.34. **3e** (255.4 mg, 74%); yellow oil. ^1^H NMR (CDCl_3_, 400 MHz) δ 7.90 (d, 1H, *J* = 7.8 Hz), 7.40 (t, 1H, *J* = 9.0 Hz), 7.25–7.23 (m, 1H), 7.14 (d, 1H, *J* = 7.4 Hz), 6.82 (s, 1H), 4.36 (d, 1H, *J* = 6.2 Hz), 3.39–3.22 (m, 1H), 2.88 (dd, 1H, *J* = 14.3, 8.2 Hz), 2.76 (dd, 1H, *J* = 14.3, 7.4 Hz), 2.26 (s, 3H), 2.15 (s, 6H), 1.97 (s, 6H), 1.24 (d, 3H, *J* = 6.5 Hz); ^13^C{^1^H} NMR (CDCl_3_, 101 MHz) δ 137.5, 137.1, 134.1, 133.8, 132.6, 132.5, 132.4, 130.4, 129.7, 125.8, 50.0, 37.1, 22.1, 20.6, 19.8, 16.0; IR ν_max_ 2918, 1315, 1125 cm^–1^. HRMS(ESI): *m*/*z* calc. for C_20_H_26_NO_2_S [M – H]^−^: 344.1690; found: 344.1686.

##### 1-(2,3,5,6-Tetramethylphenyl)-2-(*o*-nosylamino)-propane (**3f**)

1,2,4,5-Tetramethylbenzene (671.1 mg), 2-nitrobenzenesulfonamide (404.4 mg); FCC–AcOEt/hexane (4:1), R_f_: 0.37. **3f** (327.2 mg, 87%); light brown oil. ^1^H NMR (CDCl_3_, 400 MHz) δ 7.95–7.92 (m, 1H), 7.79–7.76 (m, 1H), 7.64–7.62 (m, 2H), 6.66 (s, 1H), 5.33 (d, 1H, *J* = 6.4 Hz), 3.78–3.71 (m, 1H), 2.97 (dd, 1H, *J* = 14.6, 8.6 Hz), 2.84 (dd, 1H, *J* = 14.5, 6.9 Hz), 2.08 (s, 6H), 2.07 (s, 6H), 1.31 (d, 3H, *J* = 6.5 Hz); ^13^C{^1^H} NMR (CDCl_3_, 101 MHz) δ 146.9, 134.6, 133.9, 133.8, 132.9, 132.6, 132.5, 130.34, 130.32, 125.5, 51.9, 36.9, 22.8, 20.6, 16.2; IR ν_max_ 2920, 1344, 1163 cm^–1^. HRMS(ESI): *m*/*z* calc. for C_19_H_23_N_2_O_4_S [M – H]^−^: 375.1384; found: 375.1375.

##### 1-(2,3,4,5,6-Pentamethylphenyl)-2-tosylamino-propane (**4a**)

1,2,3,4,5-Pentamethylbenzene (741.2 mg), tosylamide (342.4 mg); FCC–DCM, R_f_: 0.41. **4a** (305.3 mg, 85%); white solid, mp 169–170 °C. ^1^H NMR (CDCl_3_, 400 MHz) δ 7.46 (d, 2H, *J* = 8.2 Hz), 7.12 (d, 2H, *J* = 8.0 Hz), 4.29 (d, 1H, *J* = 6.4 Hz), 3.41–3.34 (m, 1H), 2.91 (dd, 1H, *J* = 14.5, 7.9 Hz), 2.78 (dd, 1H, *J* = 14.5, 7.3 Hz), 2.40 (s, 3H), 2.22 (s, 3H), 2.14 (s, 6H), 2.08 (s, 6H), 1.19 (d, 3H, *J* = 6.4 Hz); ^13^C{^1^H} NMR (CDCl_3_, 101 MHz) δ 142.8, 137.1, 133.3, 132.8, 132.1, 131.2, 129.2, 126.9, 50.5, 37.5, 21.9, 21.5, 17.1, 16.9, 16.8; IR ν_max_ 2919, 1319, 1135 cm^–1^. HRMS(ESI): *m*/*z* calc. for C_21_H_28_NO_2_S [M – H]^−^: 358.1846; found: 358.1835.

##### 1-(2,3,4,5,6-Pentamethylphenyl)-2-(*p*-nosylamino)-propane (**4b**)

1,2,3,4,5-Pentamethylbenzene (741.2 mg), 4-nitrobenzenesulfonamide (404.4 mg); FCC–AcOEt/hexane (2:3), R_f_: 0.35. **4b** (343.3 mg, 88%); orange solid; mp 183–185 °C. ^1^H NMR (CDCl_3_, 400 MHz) δ 8.03 (d, 2H, *J* = 8.7 Hz), 7.57 (d, 2H, *J* = 8.7 Hz), 4.46 (d, 1H, *J* = 8.2 Hz), 3.55–3.48 (m, 1H), 2.86 (dd, 1H, *J* = 14.8, 9.8 Hz), 2.76 (dd, 1H, *J* = 14.9, 5.2 Hz), 2.15 (s, 3H), 2.07 (s, 12H), 1.38 (d, 3H, *J* = 6.4 Hz); ^13^C{^1^H} NMR (CDCl_3_, 101 MHz) δ 149.2, 145.8, 133.8, 132.9, 131.7, 131.1, 127.6, 123.4, 51.7, 37.1, 23.4, 17.1, 16.8, 16.7; IR ν_max_ 2920, 1349, 1165 cm^–1^. HRMS(ESI): *m*/*z* calc. for C_20_H_25_N_2_O_4_S [M – H]^−^: 389.1541; found: 389.1530.

##### 1-(2,3,4,5,6-Pentamethylphenyl)-2-(2-methylbenzenesulfonamido)-propane (**4c**)

1,2,3,4,5-Pentamethylbenzene (741.2 mg), 2-methylbenzenesulfonamide (342.4 mg); FCC–AcOEt/hexane (2:3), R_f_: 0.35. **4c** (276.6 mg, 77%); brown solid; mp 115–117 °C. ^1^H NMR (CDCl_3_, 400 MHz) δ 7.92 (d, 1H, *J* = 7.8 Hz), 7.42 (t, 1H, *J* = 7.4 Hz), 7.26 (t, 1H, *J* = 7.6 Hz), 7.12 (d, 1H, *J* = 7.5 Hz), 4.50 (d, 1H, *J* = 6.4 Hz), 3.42–3.32 (m, 1H), 2.92 (dd, 1H, *J* = 14.5, 8.2 Hz), 2.81 (dd, 1H, *J* = 14.5, 7.3 Hz), 2.28 (s, 3H), 2.23 (s, 3H), 2.15 (s, 6H), 2.06 (s, 6H), 1.27 (d, 3H, *J* = 6.4 Hz); ^13^C{^1^H} NMR (CDCl_3_, 101 MHz) δ 137.6, 137.1, 133.4, 132.8, 132.4, 132.2, 132.1, 131.2, 129.7, 125.7, 50.4, 37.4, 22.2, 19.8, 17.0, 16.94, 16.91; IR ν_max_ 2932, 1347, 1126 cm^–1^. HRMS(ESI): *m*/*z* calc. for C_21_H_28_NO_2_S [M – H]^−^: 358.1846; found: 358.1835.

##### 1-(2,3,4,5,6-Pentamethylphenyl)-2-(*o*-nosylamino)-propane (**4d**)

1,2,3,4,5-Pentamethylbenzene (741.2 mg), 2-nitrobenzenesulfonamide (404.4 mg); FCC–AcOEt/hexane (2:3), R_f_: 0.33. **4d** (347.5 mg, 89%); yellow solid; mp 130–132 °C. ^1^H NMR (CDCl_3_, 400 MHz) δ 7.92 (d, 1H, *J* = 9.2 Hz), 7.74 (d, 1H, *J* = 9.4 Hz), 7.65–7.57 (m, 2H), 5.35 (d, 1H, *J* = 6.3 Hz), 3.77–3.70 (m, 1H), 2.98 (dd, 1H, *J* = 14.8, 8.9 Hz), 2.86 (dd, 1H, *J* = 14.8, 6.6 Hz), 2.13 (s, 6H), 2.11 (s, 3H), 2.04 (s, 6H), 1.33 (d, 3H, *J* = 6.5 Hz); ^13^C{^1^H} NMR (CDCl_3_, 101 MHz) δ 146.7, 134.6, 133.1, 132.7, 132.6, 132.5, 132.1, 131.1, 130.4, 125.3, 52.0, 37.1, 22.9, 17.2, 16.9, 16.8; IR ν _max_ 2922, 1346, 1128 cm^–1^. HRMS(ESI): *m*/*z* calc. for C_20_H_25_N_2_O_4_S [M – H]^−^: 389.1541; found: 389.1530.

##### 1-(2,5-Dimethylphenyl)-2-tosylamino-propane (**5a**)

*p*-Xylene (0.62 mL); tosylamide (342.4 mg); FCC–AcOEt/hexane (2:3). **5a** (231.5 mg, 73%); light brown oil. ^1^H NMR (CDCl_3_, 400 MHz) δ 7.59 (d, 2H, *J* = 8.2 Hz), 7.20 (d, 2H, *J* = 8.1 Hz), 6.94–6.89 (m, 2H), 6.76 (s, 1H), 4.72 (d, 1H, *J* = 6.7 Hz), 3.48–3.41 (m, 1H), 2.73 (dd, 1H, *J* = 13.7, 6.9 Hz), 2.59 (dd, 1H, *J* = 13.7, 7.4 Hz), 2.41 (s, 3H), 2.24 (s, 3H), 2.09 (s, 3H), 1.15 (d, 3H, *J* = 6.4 Hz); ^13^C{^1^H} NMR (CDCl_3_, 101 MHz) δ 143.0, 137.5, 135.4, 135.3, 133.2, 130.9, 130.5, 129.5, 127.5, 126.9, 50.1, 41.1, 21.7, 21.5, 20.9, 18.8. The characterization of product **5a** is consistent with that reported in the literature.^5^

##### 1-(2,5-Dimethylphenyl)-2-(*p*-nosylamino)-propane (**5b**)

*p*-Xylene (0.62 mL); 4-nitrobenzenesulfonamide (404.4 mg); FCC–AcOEt/hexane (1:2), R_f_: 031. **5b** (261.1 mg, 75%); light brown oil. ^1^H NMR (CDCl_3_, 400 MHz) δ 8.13 (d, 2H, *J* = 8.8 Hz), 7.69 (d, 2H, *J* = 8.8 Hz), 6.89–6.84 (m, 2H), 6.69 (s, 1H), 4.49 (d, 1H, *J* = 7.4 Hz), 3.58–3.48 (m, 1H), 2.72 (dd, 1H, *J* = 14.0, 5.5 Hz), 2.58 (dd, 1H, *J* = 14.0, 8.9 Hz), 2.19 (s, 3H), 2.09 (s, 3H), 1.31 (d, 3H, *J* = 6.4 Hz); ^13^C{^1^H} NMR (CDCl_3_, 101 MHz) δ 149.6, 149.1, 146.1, 135.6, 135.1, 130.8, 130.6, 127.8 (2CH), 123.9, 51.1, 40.9, 23.0, 20.8, 18.8; IR ν_max_ 2921, 1377, 1161 cm^–1^. HRMS(ESI): *m*/*z* calc. for C_17_H_19_N_2_O_4_S [M – H]^−^: 347.1071; found: 347.1055.

##### 1-(2,5-Dimethylphenyl)-2-(4-chlorobenzenesulfonamido)-propane (**5c**)

*p*-Xylene (0.62 mL); 4-chlorobenzenesulfonamide (383.3 mg); FCC–DCM/MeOH (9.9:0.1), R_f_: 0.43. **5c** (229.2 mg, 68%); light brown oil. ^1^H NMR (CDCl_3_, 400 MHz) δ 7.54 (d, 2H, *J* = 8.5 Hz), 7.32 (d, 2H, *J* = 8.6 Hz), 6.92 (s, 2H), 6.73 (s, 1H), 4.68 (d, 1H, *J* = 6.9 Hz), 3.49–3.42 (m, 1H), 2.64 (d, 2H, *J* = 7.2 Hz), 2.23 (s, 3H), 2.10 (s, 3H), 1.23 (d, 3H, *J* = 6.4 Hz); ^13^C{^1^H} NMR (CDCl_3_, 101 MHz) δ 138.9, 138.7, 135.5, 135.3, 132.9, 130.8, 130.6, 129.1, 128.2, 127.6, 50.5, 41.0, 22.3, 20.8, 18.8; IR ν_max_ 2924, 1322, 1159 cm^–1^. HRMS(ESI): *m*/*z* calc. for C_17_H_19_ClNO_2_S [M – H]^−^: 336.0831; found: 336.0828.

##### 1-(2,4,6-Trimethoxyphenyl)-2-tosylamino-propane (**6**)

1,3,5-Trimethoxybenzene (890.9 mg); tosylamide (342.4 mg); FCC–AcOEt/hexane (2:3), R_f_: 0.34. **6** (295.7 mg, 78%); colorless oil. ^1^H NMR (CDCl_3_, 400 MHz) δ 7.34 (d, 2H, *J* = 8.1 Hz), 7.00 (d, 2H, *J* = 8.0 Hz), 5.93 (s, 2H), 5.09 (d, 1H, *J* = 5.4 Hz), 3.80 (s, 3H), 3.69 (s, 6H), 3.32–3.26 (m, 1H), 2.61–2.49 (m, 2H), 2.37 (s, 3H), 1.30 (d, 3H, *J* = 6.4 Hz); ^13^C{^1^H} NMR (CDCl_3_, 101 MHz) δ 160.0, 158.4, 141.9, 137.1, 128.9, 126.6, 106.6, 90.4, 55.5, 55.2, 50.8, 29.4, 23.5, 21.4; IR ν_max_ 2920, 1379, 1207, 1160 cm^–1^. HRMS(ESI): *m*/*z* calc. for C_19_H_24_NO_5_S [M – H]^−^: 378.1381; found: 378.1359.

##### 1-(2,5-Dimethoxyphenyl)-2-tosylamino-propane (**7**)

1,4-Dimethoxybenzene (690.8 mg); tosylamide (342.4 mg); FCC–AcOEt/hexane (2:3), R_f_: 0.32. **7** (251.4 mg, 72%); colorless oil. ^1^H NMR (CDCl_3_, 400 MHz) δ 7.43 (d, 2H, *J* = 8.1 Hz), 7.08 (d, 2H, *J* = 8.9 Hz), 6.68 (s, 2H), 6.41 (s, 1H), 5.06 (d, 1H, *J* = 5.4 Hz), 3.74 (s, 3H), 3.69 (s, 3H), 3.45–3.36 (m, 1H), 2.73 (dd, 1H, *J* = 13.6, 9.0 Hz), 2.49 (dd, 1H, *J* = 13.6, 4.9 Hz), 2.37 (s, 3H), 1.25 (d, 3H, *J* = 6.6 Hz); ^13^C{^1^H} NMR (CDCl_3_, 101 MHz) δ 153.7, 151.3, 142.5, 137.1, 129.2, 127.0, 126.8, 116.7, 112.4, 111.5, 55.9, 55.5, 51.2, 37.5, 22.8, 21.4; IR ν_max_ 2929, 1223, 1155 cm^–1^. HRMS(ESI): *m*/*z* calc. for C_18_H_22_NO_4_S [M – H]^−^: 348.1275; found: 348.1273.

##### 1-(5-Methylanisole)-2-tosylamino-propane (**8**)

4-Methylanisole (0.63 mL); tosylamide (342.4 mg); FCC–DCM, R_f_: 0.31. **8** (219.9 mg, 66%); yellow oil. ^1^H NMR (CDCl_3_, 400 MHz) δ 7.42 (d, 2H, *J* = 8.2 Hz), 7.08 (d, 2H, *J* = 8.1 Hz), 6.94 (d, 1H, *J* = 8.3 Hz), 6.66–6.63 (m, 2H), 4.99 (d, 1H, *J* = 5.3 Hz), 3.74 (s, 3H), 3.45–3.38 (m, 1H), 2.69 (dd, 1H, *J* = 13.6, 8.9 Hz), 2.51 (dd, 1H, *J* = 13.6, 4.9 Hz), 2.38 (s, 3H), 2.18 (s, 3H), 1.25 (d, 3H, *J* = 6.6 Hz); ^13^C{^1^H} NMR (CDCl_3_, 101 MHz) δ 169.4, 142.4, 137.2, 131.8, 130.0, 129.2, 128.2, 126.8, 125.7, 110.4, 55.4, 51.2, 37.3, 27.1, 22.8, 21.4. The characterization of product **8** is consistent with that reported in the literature.^5^

##### 1-(5-Chloroanisole)-2-tosylamino-propane (**9**)

4-Chloroanisole (0.63 mL); tosylamide (342.4 mg); FCC–AcOEt/hexane (2:3), R_f_: 0.33. **9** (243.6 mg, 69%); light yellow oil. ^1^H NMR (CDCl_3_, 400 MHz) δ 7.44 (d, 2H, *J* = 8.0 Hz), 7.11 (d, 2H, *J* = 7.9 Hz), 7.09–7.06 (m, 1H), 6.80 (d, 1H, *J* = 2.2 Hz), 6.65 (d, 1H, *J* = 8.7 Hz), 4.79 (d, 1H, *J* = 5.9 Hz), 3.77 (s, 3H), 3.45–3.42 (m, 1H), 2.71 (dd, 1H, *J* = 13.5, 9.3 Hz), 2.49 (dd, 1H, *J* = 13.7, 5.0 Hz), 2.39 (s, 3H), 1.26 (d, 3H, *J* = 5.9 Hz); ^13^C NMR (CDCl_3_, 101 MHz) δ 155.7, 144.3, 142.8, 130.7, 129.3, 127.9, 127.5, 126.7, 125.7, 111.6, 55.7, 51.1, 37.1, 22.9, 21.5; IR ν_max_ 2918, 1326, 1157 cm^–1^. HRMS(ESI): *m*/*z* calc. for C_17_H_19_ClNO_3_S [M – H]^−^: 352.0780; found: 352.0778.

##### 1-(5-Bromoanisole)-2-tosylamino-propane (**10**)

4-Bromoanisole (0.63 mL); tosylamide (342.4 mg); FCC–AcOEt/hexane (2:3). **10** (250.1 mg, 63%); yellow oil. ^1^H NMR (CDCl_3_, 400 MHz) δ 7.43 (d, 2H, *J* = 8.2 Hz), 7.23–7.20 (m, 1H), 7.11 (d, 2H, *J* = 8.1 Hz), 6.95 (d, 1H, *J* = 2.3 Hz), 6.59 (d, 1H, *J* = 8.7 Hz), 4.79 (d, 1H, *J* = 6.2 Hz), 3.77 (s, 3H), 3.47–3.39 (m, 1H), 2.71 (dd, 1H, *J* = 13.6, 10.7 Hz), 2.49 (dd, 1H, *J* = 13.6, 4.9 Hz), 2.40 (s, 3H), 1.26 (d, 3H, *J* = 3.6 Hz); ^13^C{^1^H} NMR (CDCl_3_, 101 MHz) δ 156.2, 142.8, 136.9, 133.5, 130.5, 129.4, 128.4, 126.7, 113.1, 112.1, 55.6, 51.2, 37.1, 23.0, 21.5. The characterization of product **10** is consistent with that reported in the literature.^5^

##### 1-(5-Bromoanisole)-2-tosylamino-propane (**11**)

3-Bromoanisole (0.63 mL); tosylamide (342.4 mg); FCC–AcOEt/hexane (1:4), R_f_: 0.29. **11** (281.9 mg, 71%); light brown oil. ^1^H NMR (CDCl_3_, 400 MHz) δ 7.54 (d, 2H, *J* = 8.3 Hz), 7.15 (d, 2H, *J* = 8.3 Hz), 6.93 (d, 2H, *J* = 7.8 Hz), 6.67 (dd, 1H, *J* = 8.4, 2.6 Hz), 4.39 (d, 1H, *J* = 6.2 Hz), 3.77 (s, 3H), 3.63–3.55 (m, 1H), 2.73 (d, 2H, *J* = 7.2 Hz), 2.39 (s, 3H), 1.21 (d, 3H, *J* = 6.5 Hz); ^13^C{^1^H} NMR (CDCl_3_, 101 MHz) δ 158.9, 142.8, 137.4, 131.6, 129.4, 128.9, 126.9, 124.7, 118.1, 113.5, 55.4, 50.5, 42.3, 22.3, 21.4; IR ν_max_ 2918, 1378, 1199 cm^–1^. HRMS(ESI): *m*/*z* calc. for C_17_H_19_BrNO_3_S [M – H]^−^ HRMS(ESI): 396.0275; found: 396.0261.

##### 1-(2-Trimethoxyphenyl)- 2-tosylamino-propane and 1-(4-trimethoxyphenyl)-2-tosylamino-propane (**12a** + **12b**)

Anisole (0.53 mL); tosylamide (342.4 mg); FCC–DCM/MeOH (9.5:0.5). **12a** + **12b** (236.2 mg, 74%, isomeric ratio after purification: 1.3/1); light yellow oil. ^1^H NMR (CDCl_3_, 400 MHz) compound **12a** δ 7.45 (d, 2H, *J* = 8.2 Hz), 7.16 (t, 1H, *J* = 7.6 Hz), 7.09 (d, 2H, *J* = 8.0 Hz), 6.89 (d, 1H, *J* = 7.4 Hz), 6.79 (t, 1H, *J* = 7.3 Hz), 6.72 (d, 1H, *J* = 7.6 Hz), 4.83 (d, 1H, *J* = 5.7 Hz), 3.67 (s, 3H), 3.45–3.34 (m, 1H), 2.71 (dd, 1H, *J* = 13.6, 8.6 Hz), 2.63–2.65 (m, 1H), 2.29 (s, 3H),1.14 (d, 3H, *J* = 6.4 Hz); compound **12b** δ 7.61 (d, 2H, *J* = 8.2 Hz), 7.22 (d, 2H, *J* = 7.9 Hz), 6.92 (d, 2H, *J* = 8.4 Hz), 6.75 (d, 1H, *J* = 8.4 Hz), 4.20 (d, 1H, *J* = 7.0 Hz), 3.71 (s, 3H), 3.45–3.34 (m, 1H), 2.63–2.56 (m, 2H), 2.34 (s, 3H), 1.01 (d, 3H, *J* = 6.5 Hz). The characterization of product **12a** is consistent with that reported in the literature.^19^ The characterization of product **12b** is consistent with that reported in the literature.^20^

##### 1-(3-Thienyl)-2-tosylamino-propane (**13b**)

Thiophene (0.40 mL); tosylamide (342.4 mg); FCC–DCM/MeOH (9.5:0.5). **13b** (115.1 mg, 39%); light yellow oil. ^1^H NMR (CDCl_3_, 400 MHz) δ 7.66 (d, 2H, *J* = 7.9 Hz), 7.26 (d, 2H, *J* = 8.0 Hz), 7.19 (dd, 1H, *J* = 4.9, 3.0 Hz), 6.88 (d, 1H, *J* = 2.1 Hz), 6.76 (d, 1H, *J* = 4.6 Hz), 4.25 (d, 1H, *J* = 6.4 Hz), 3.57–3.50 (m, 1H), 2.71 (d, 2H, *J* = 5.1 Hz), 2.42 (s, 3H), 1.09 (d, 3H, *J* = 6.5 Hz). The characterization of product **13b** is consistent with that reported in the literature.^21^

#### General Procedure for the Synthesis of Arylated/Hydroaminated Products with Different Substituted Alcohols

In a sealed tube, after 15 min, the crotyl alcohol or 1-buten-3-ol (1.0 mmol, 72 mg), hydrocarbons (5.0 mmol), and sulfonamide (2.0 mmol) were added to a solution of Cu(OTf)_2_ (10 mol %, 36.2 mg) and xantphos (20 mol %, 11.6 mg) in chlorobenzene (0.25 M). The resulted solution was magnetically stirred and heated at 75 °C in an oil bath for 24 h. The reaction mixture was washed with brine (3 × 5 mL) and the organic layer was extracted with AcOEt (2 × 5 mL), dried over MgSO_4_, and filtered. The solvent was evaporated under reduced pressure. The residue was purified by FCC. Starting from the aromatic source and appropriate sulfonamide, yield, physical, spectroscopic, and analytical data of compounds **14a**-**b**, **15a**-**b** and **16**-**17** are as follows.

##### 1-(2,4,6-Trimethylphenyl)-2-(benzenesulfonamido)-butane and 1-(2,4,6-trimethylphenyl)-3-(benzenesulfonamido)-butane (**14a** + **14b**)

Mesitylene (0.69 mL); benzenesulfonamide (314.4 mg); FCC–MeOH/DCM (1:50), R_f_: 0.37. **14a** + **14b** (218.6 mg, 66%, isomeric ratio after purification: 1/1.4); colorless oil. ^1^H NMR (CDCl_3_, 400 MHz) compound **14a** δ 7.63 (d, 2H, *J* = 8.8 Hz), 7.52–7.48 (m, 2H), 7.36 (t, 1H, *J* = 7.8 Hz), 6.70 (s, 2H), 4.33 (d, 1H, *J* = 7.7 Hz), 3.39–3.33 (m, 1H), 2.78 (dd, 1H, *J* = 14.1, 7.8 Hz), 2.68 (dd, 1H, *J* = 14.2, 7.6 Hz), 2.22 (s, 3H), 2.15 (s, 6H), 1.66–1.38 (m, 2H), 0.84 (t, 3H, *J* = 7.3 Hz); compound **14b** δ 7.92 (d, 2H, *J* = 9.0 Hz), 7.52–7.48 (m, 2H), 7.36 (t, 1H, *J* = 7.8 Hz), 6.80 (s, 2H), 4.38 (d, 1H, *J* = 8.7 Hz), 3.50–3.43 (m, 1H), 2.58–2.49 (m, 2H), 2.23 (s, 3H), 2.18 (s, 6H), 1.66–1.39 (m, 2H), 1.14 (d, 3H, J = 6.6 Hz); ^13^C{^1^H} NMR (CDCl_3_, 101 MHz) δ 141.2, 140.5, 136.5, 135.7, 135.6, 135.2, 134.9, 132.6, 132.1, 131.4, 129.3, 129.1, 128.9, 128.7, 126.9, 126.8, 55.6, 50.7, 36.8, 34.8, 28.2, 25.5, 21.8, 20.77, 20.75, 20.3, 19.6, 10.0; IR ν _max_ 2965, 1322, 1158 cm^–1^. HRMS(ESI): *m*/*z* calc. for C_19_H_24_NO_2_S [M – H]^−^: 330.1533; found: 330.1531.

##### 1-(2,4,6-Trimethylphenyl)-2-(4-chlorobenzenesulfonamido)-butane and 1-(2,4,6-trimethylphenyl)-3-(4-chlorobenzenesulfonamido)-butane (**15a** + **15b**)

Mesitylene (0.69 mL); 4-chlorobenzenesulfonamide (383.3 mg); FCC–DCM, R_f_: 0.34. **15a** + **15b** (222.7 mg, 61%, isomeric ratio after purification: 1/1); colorless oil. ^1^H NMR (CDCl_3_, 400 MHz) compound **15a** δ 7.73 (d, 2H, *J* = 8.5 Hz), 7.44–7.44 (m, 2H), 6.90 (s, 2H), 4.31 (d, 1H, *J* = 7.8 Hz), 3.41–3.34 (m, 1H), 2.75–2.66 (m, 2H), 2.31 (s, 3H), 2.14 (s, 6H), 1.67–1.57 (m, 2H), 0.93 (t, 3H, *J* = 7.9 Hz); compound **15b** δ 7.84 (d, 2H, *J* = 8.4 Hz), 7.49 (d, 2H, *J* = 8.4 Hz), 6.81 (s, 2H), 4.37 (d, 1H, *J* = 8.3 Hz), 3.49–3.42 (m, 1H), 2.53–2.51 (m, 1H), 2.49–2.40 (m, 1H), 2.24 (s, 3H), 2.19 (s, 6H), 1.54–1.40 (m, 2H), 1.16 (d, 3H, *J* = 6.6 Hz); ^13^C{^1^H} NMR (CDCl_3_, 101 MHz) δ 143.7, 142.1, 140.1, 139.0, 136.3, 136.0, 135.6, 135.3, 134.8, 132.3, 131.3, 129.4, 128.9, 128.8, 128.0, 127.7, 55.9, 50.8, 36.8, 34.4, 29.1, 25.5, 22.8, 21.7, 20.8, 20.3, 19.5, 10.1; IR ν _max_ 2919, 1314, 1155 cm^–1^. HRMS(ESI): *m*/*z* calc. for C_19_H_23_ClNO_2_S [M – H]^−^: 364.1144; found: 364.1128.

##### 1-(2,4,6-Trimethylphenyl)-2-(*o*-nosylamino)-butane (**16**)

Mesitylene (0.69 mL); 4-nitrobenzenesulfonamide (404.4 mg); FCC–AcOEt/PE (1:4), R_f_: 0.33. **16** (236.9 mg, 63%) yellow oil. ^1^H NMR (CDCl_3_, 400 MHz) δ 7.82–7.76 (m, 2H), 7.64–7.54 (m, 2H), 6.54 (s, 2H), 5.26 (d, 1H, *J* = 7.9 Hz), 3.80–3.71 (m, 1H), 2.80 (dd, 1H, *J* = 14.4, 8.8 Hz), 2.75 (dd, 1H, *J* = 14.4, 6.9 Hz), 2.18 (s, 6H), 2.12 (s, 3H), 1.72–1.58 (m, 2H), 0.97 (t, 3H, *J* = 7.4 Hz); ^13^C{^1^H} NMR (CDCl_3_, 101 MHz) δ 144.4, 136.5, 135.5, 132.8, 132.4, 131.2, 129.9, 129.1 (2C), 125.3, 56.9, 34.8, 29.3, 20.7, 20.4, 10.3; IR ν _max_ 2916, 1356, 1164 cm^–1^. HRMS(ESI): *m*/*z* calc. for C_19_H_23_N_2_O_4_S [M – H]^−^: 375.1384; found: 375.1382.

##### 1-(2,4,6-Trimethylphenyl)-2-(*p*-nosylamino)-butane (**17**)

Mesitylene (0.69 mL); 4-nitrobenzenesulfonamide (404.4 mg); FCC–AcOEt/PE (1:4), R_f_: 0.33. **17** (255.8 mg, 68%); yellow oil. ^1^H NMR (CDCl_3_, 400 MHz) 8.08 (d, 2H, *J* = 8.4 Hz), 7.62 (d, 2H, *J* = 8.2 Hz), 6.59 (s, 2H), 4.35 (d, 1H, *J* = 7.8 Hz), 3.51–3.49 (m, 1H), 2.68 (d, 2H, *J* = 7.6 Hz), 2.18 (s, 3H), 2.12 (s, 6H), 1.73–1.66 (m, 2H), 1.00 (t, 3H, *J* = 7.5 Hz); ^13^C NMR (CDCl_3_, 101 MHz) δ 150.5, 146.2, 136.3, 136.2, 131.2, 129.2, 127.5, 123.7, 56.6, 34.2, 29.9, 20.6, 20.3, 10.1; IR ν _max_ 2918, 1347, 1155 cm^–1^. HRMS(ESI): *m*/*z* calc. for C_19_H_23_N_2_O_4_S [M – H]^−^: 375.1384; found: 375.1379.

## Data Availability

The data underlying this study are available in the published article and its online Supporting Materials.

## Supporting Information Available

^1^H and ^13^C NMR spectra of compounds **1a**–**h**, **3a**–**f**, **4a**–**d**, **5a**–**c**, **6**–**11**, **12a**–**b**, **13a**–**b**, **14a**–**b**, **15a**–**b**, **16** and **17** (PDF)
