# Supplementary material for: Copper(II)-Catalyzed Three-Component Arylation/Hydroamination Cascade from Allyl Alcohol: Access to 1-Aryl-2-sulfonylamino-propanes
Source: J Org Chem. 2023 Sep 25;88(19):13995–4003. doi: 10.1021/acs.joc.3c01536 (PMC10563128; doi:10.1021/acs.joc.3c01536)

Copper(II)-Catalyzed Three-Component Arylation/Hydroamination Cascade from Allyl Alcohol: Access to 1-Aryl-2-sulfonylamino-propanes

Camilla Loro, *[§] *Marta Papis,* [§] *Francesca Foschi,* [§] *Gianluigi Broggini,* [§] *Giovanni Poli,* [‡] *and Julie Oble**[‡]

[§] Dipartimento di Scienza e Alta Tecnologia, Università degli Studi dell'Insubria, Via Valleggio 9, 22100, Como, Italy; [‡] Sorbonne Université, Faculté des Sciences et Ingénierie, CNRS, Institut Parisien de Chimie Moléculaire, IPCM, 4 place Jussieu, 75005 Paris, France.

camilla.loro@uninsubria.it; julie.oble@sorbonne-universite.fr

Table of contents

^{1}H and ^{13}C NMR Spectra S-2

^{1}H NMR and ^{13}C NMR

1-(2,4,6-Trimethylphenyl)-2-tosylamino-propane (1a)

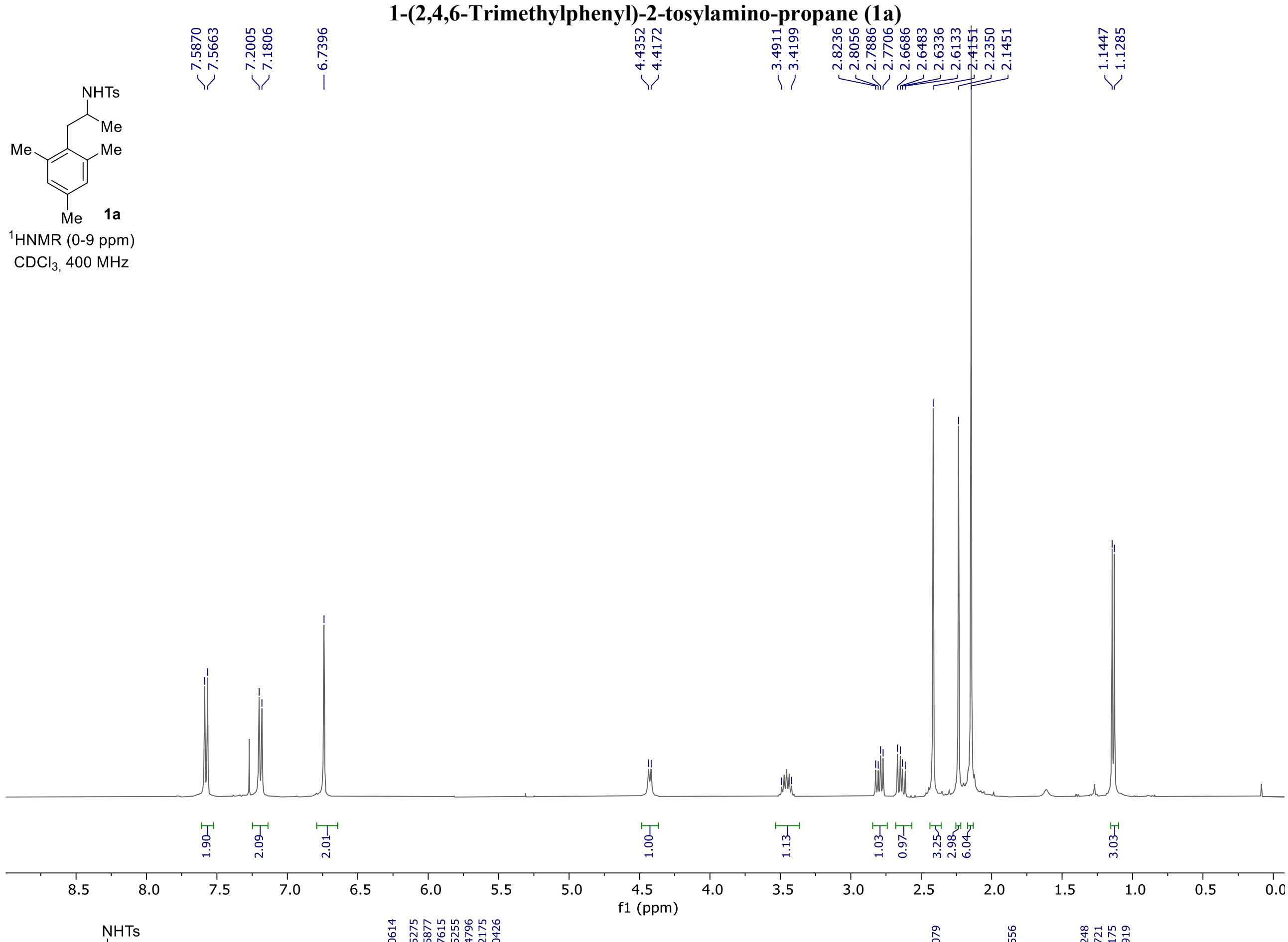

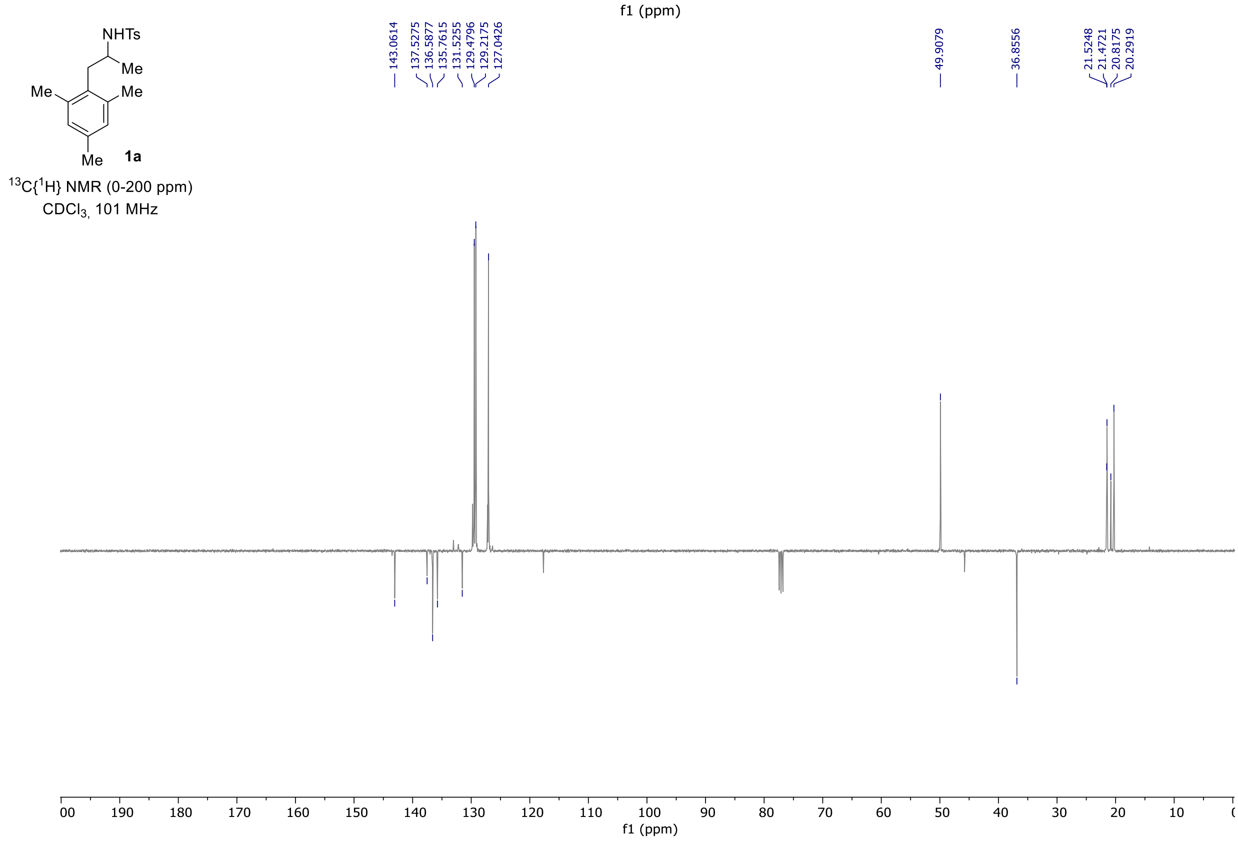

1-(2,4,6-Trimethylphenyl)-2-(*p*-nosylamino)-propane (1b)

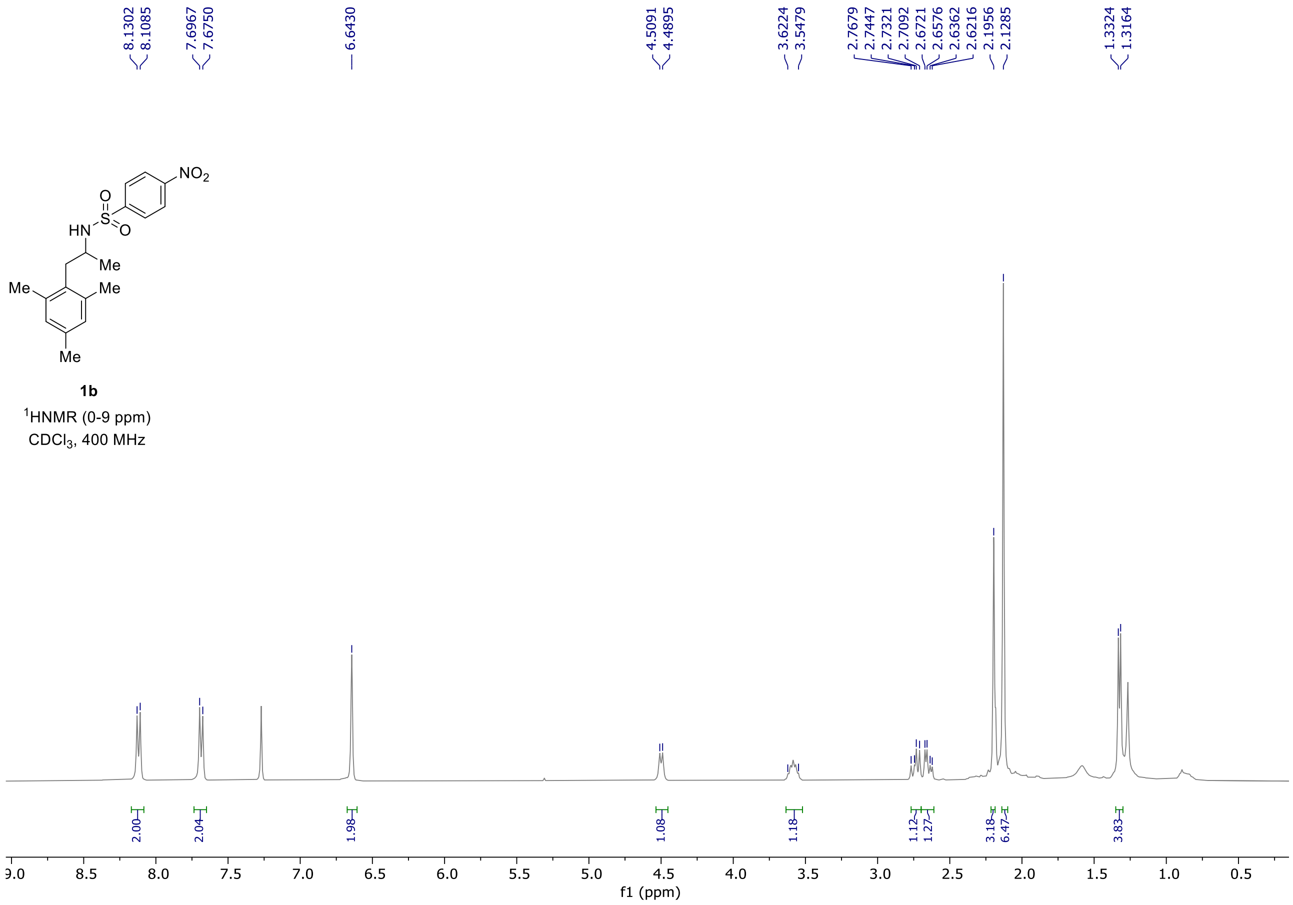

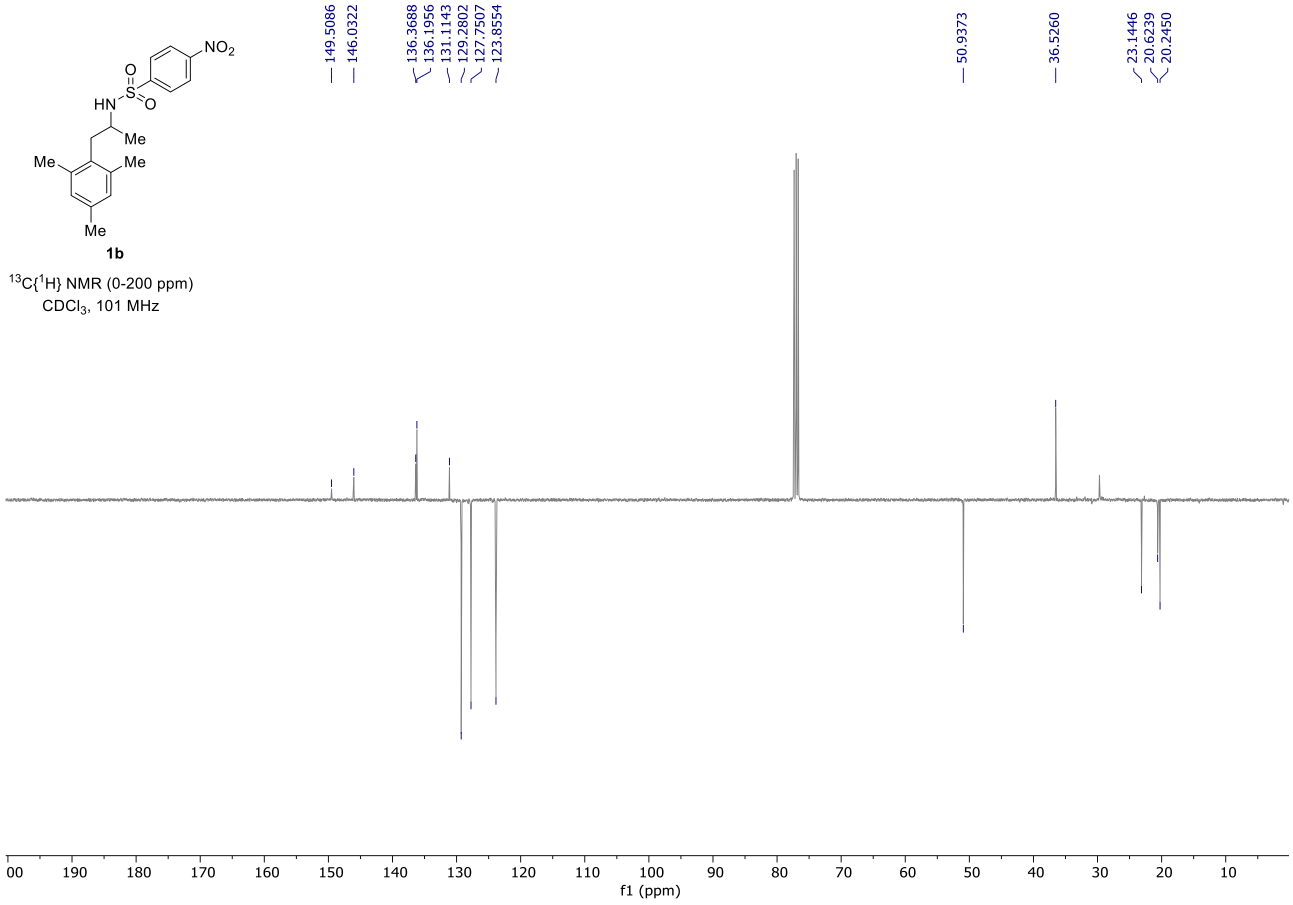

1-(2,4,6-Trimethylphenyl)-2-(4-chlorobenzenesulfonamido)-propane (1c)

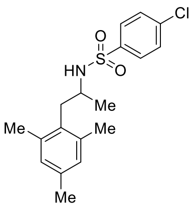

1c

^{1}HNMR (0-9 ppm)

$CDCl_3$, 400 MHz

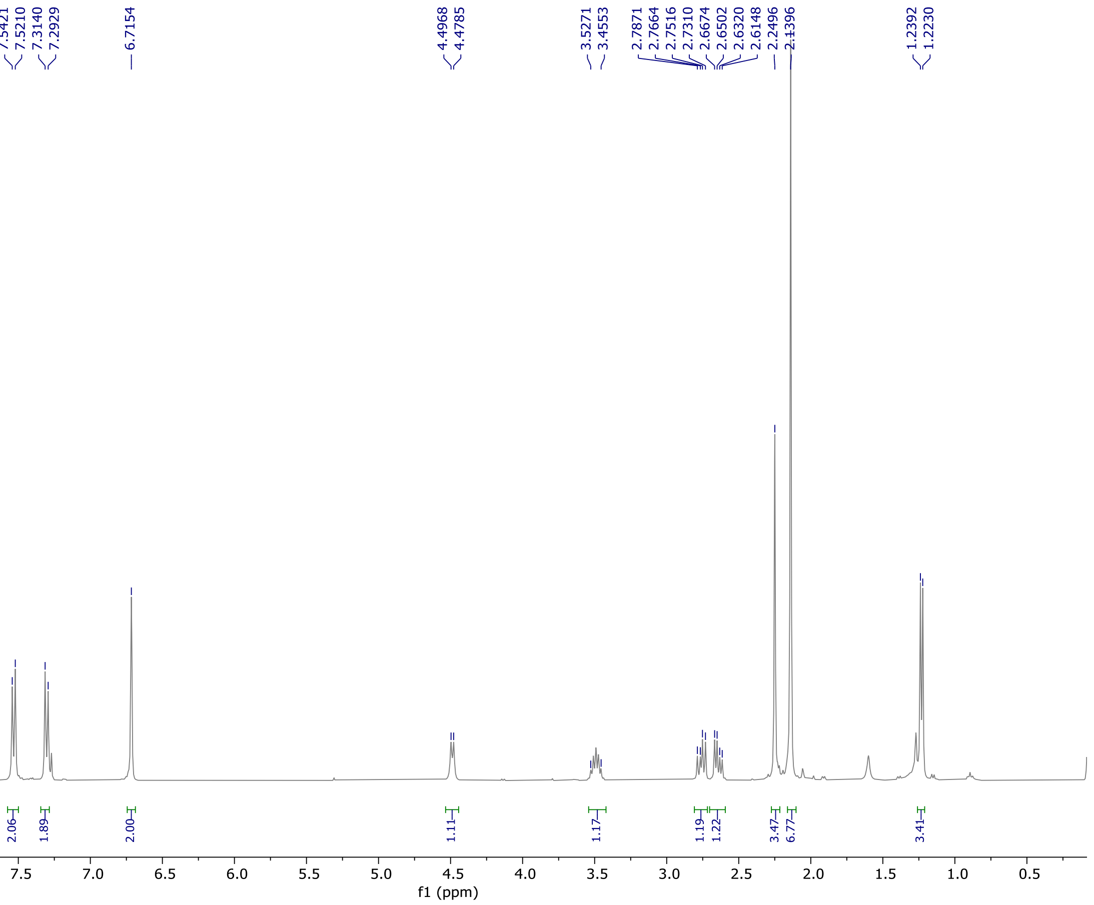

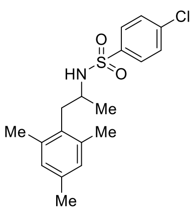

1c

$^{13}C\{^{1}H\}$ NMR (0-200 ppm)

$CDCl_3$, 101 MHz

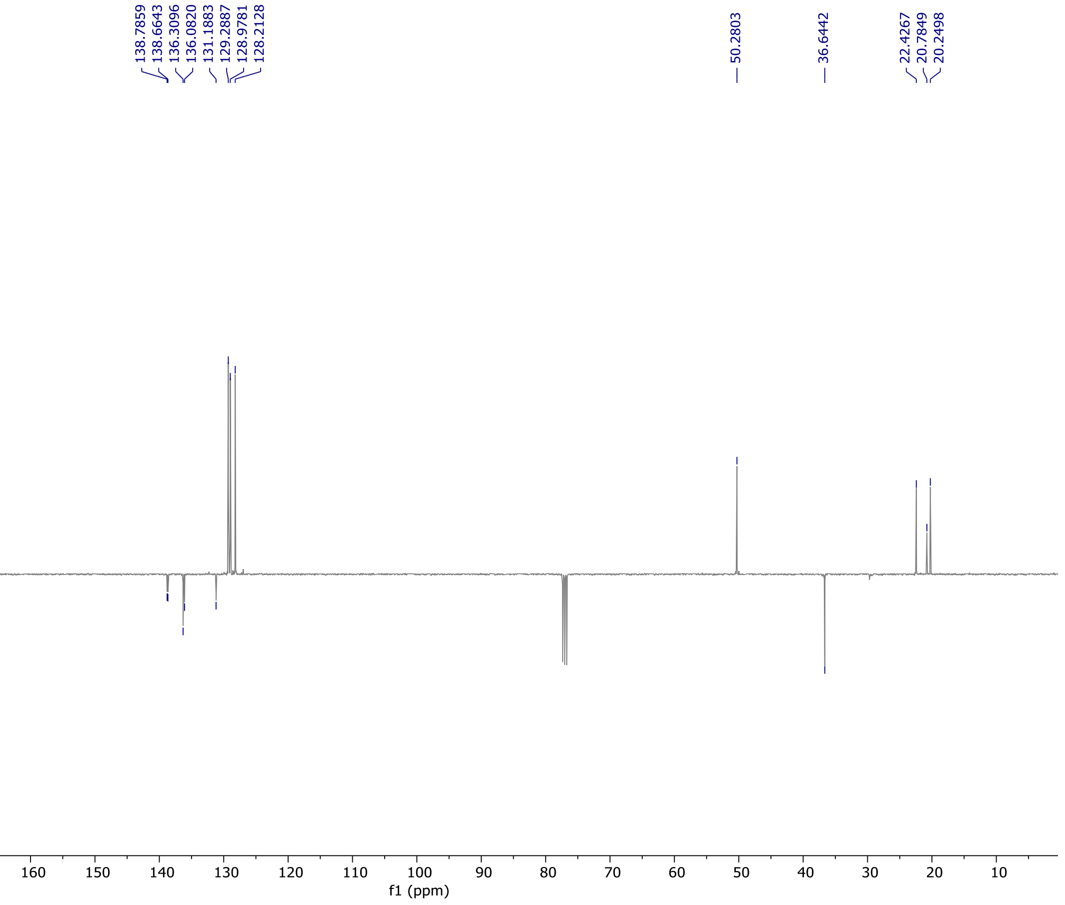

1-(2,4,6-Trimethylphenyl)-2-(4-trifluoromethylbenzenesulfonamido)-propane (1d)

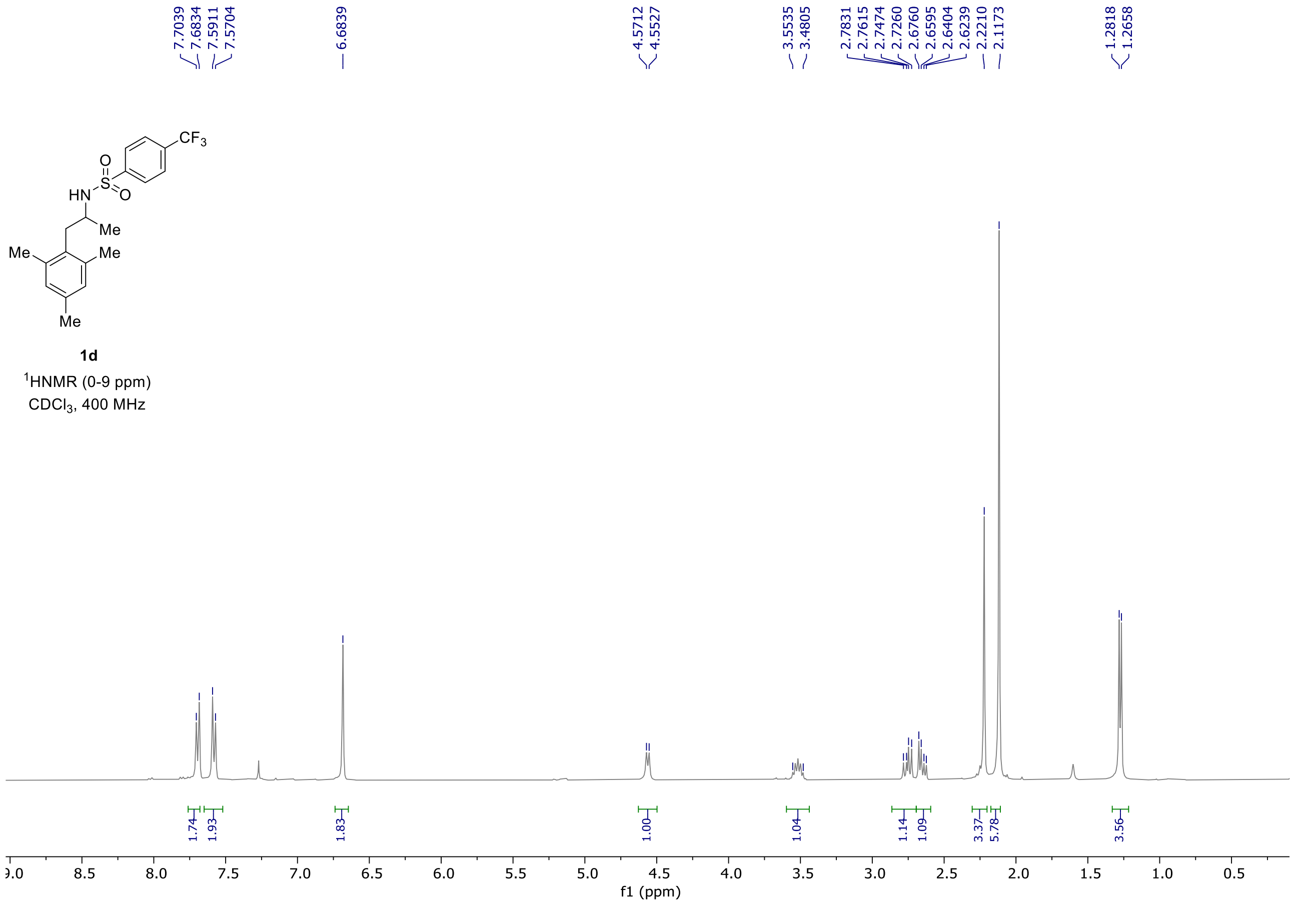

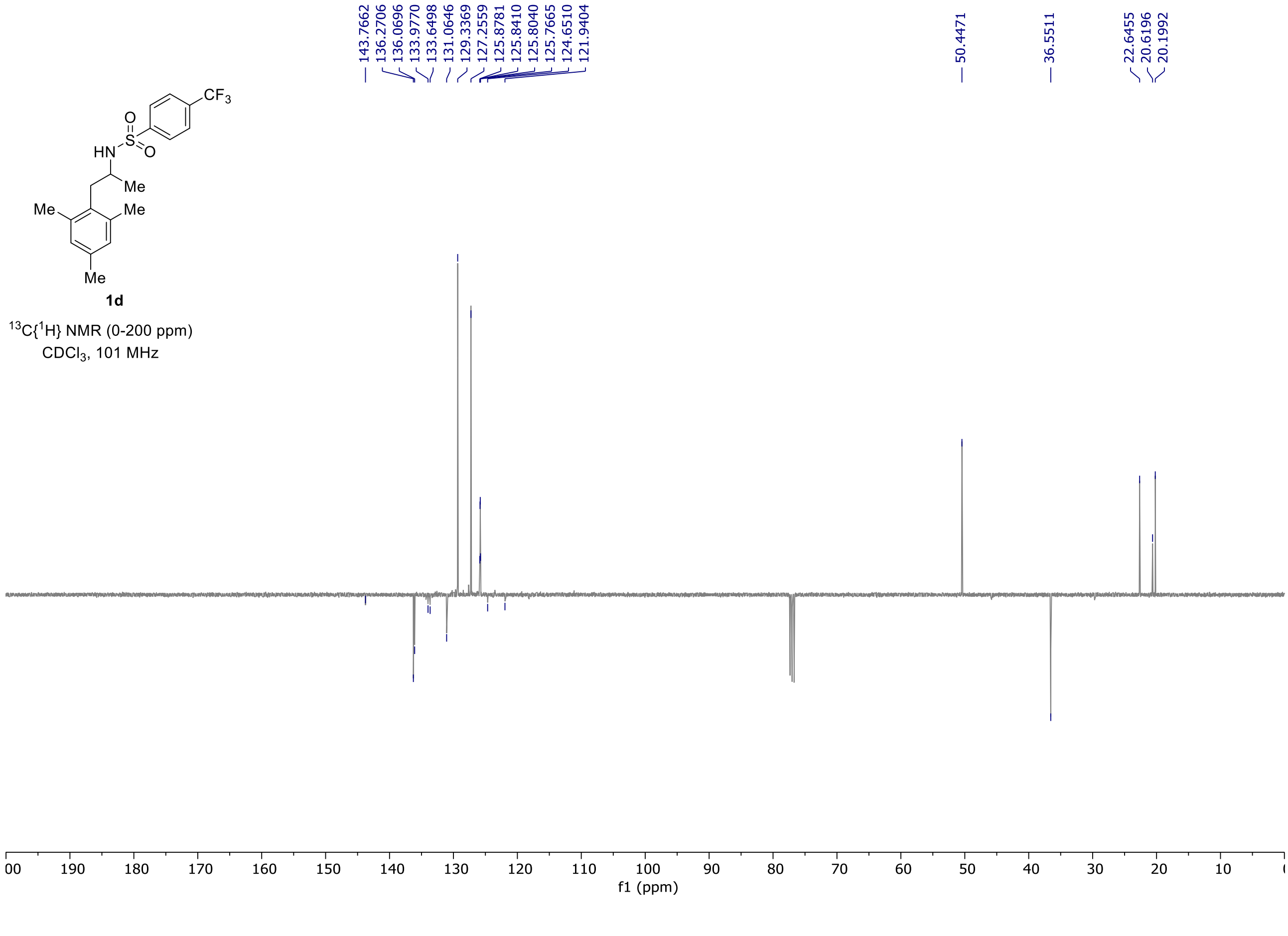

1-(2,4,6-Trimethylphenyl)-2-(2-methylbenzensulfonamindo)-propane (1e)

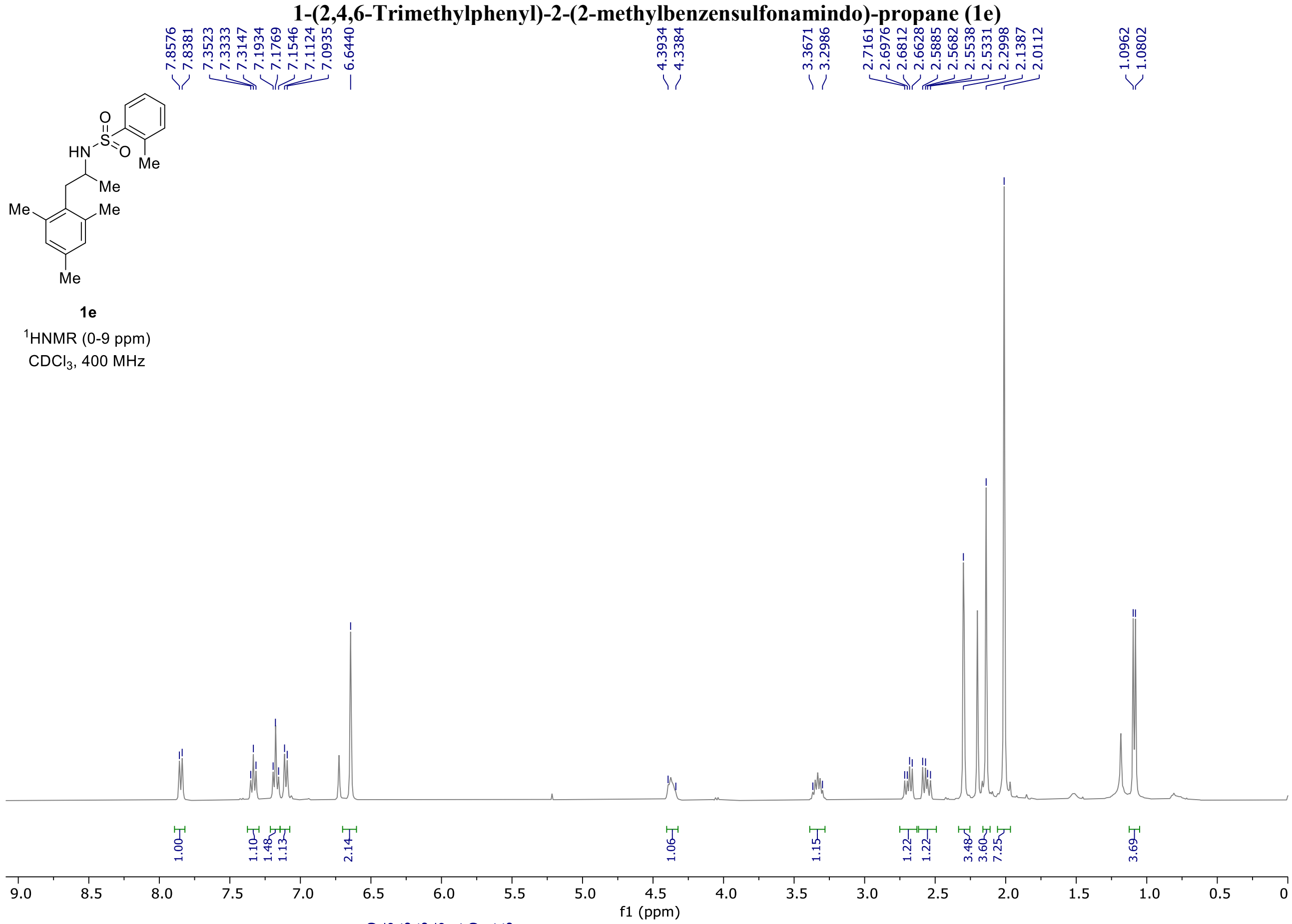

1e
^{1}HNMR (0-9 ppm)
$CDCl_3$, 400 MHz

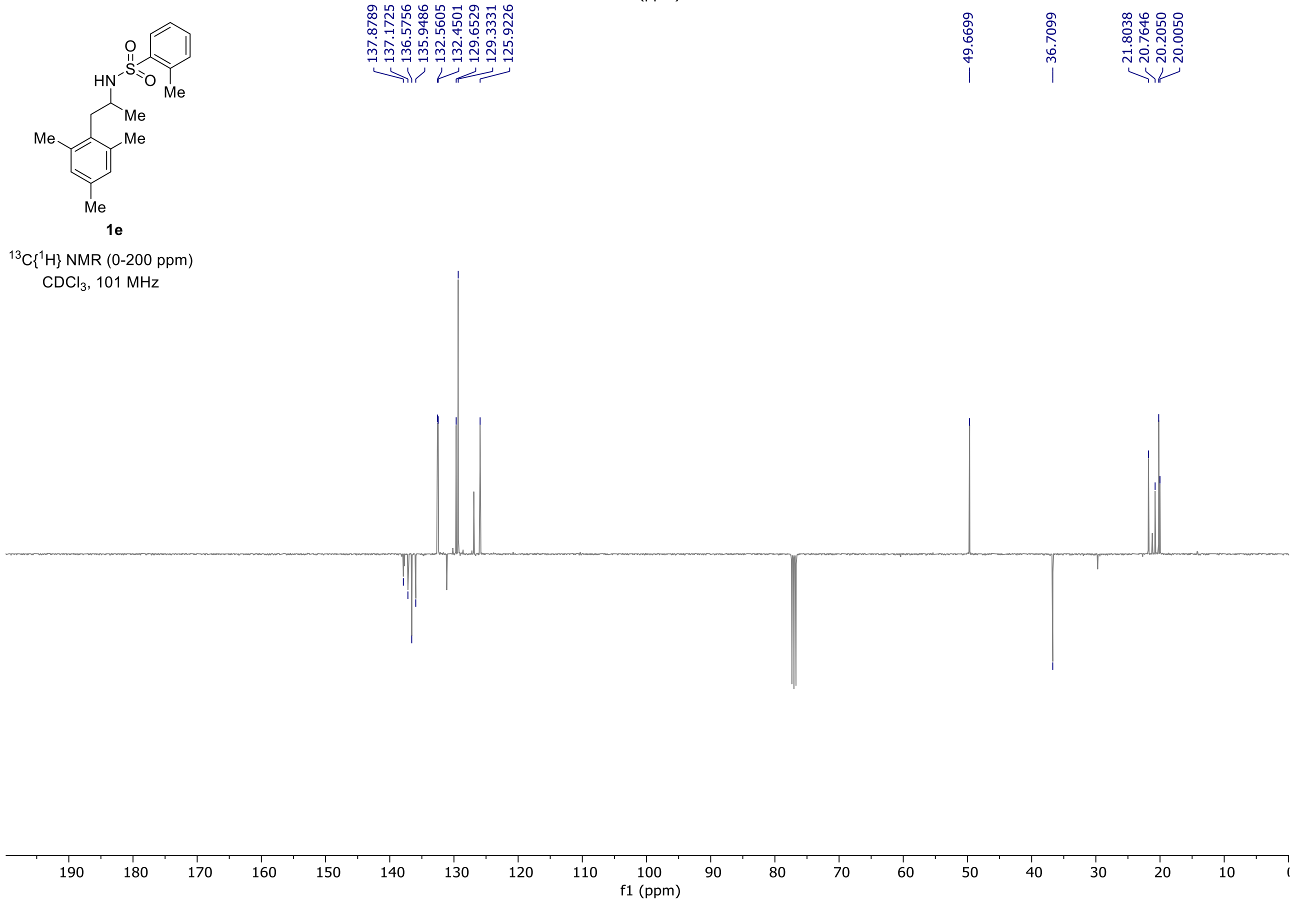

1e
$^{13}C\{^{1}H\}$ NMR (0-200 ppm)
$CDCl_3$, 101 MHz

1-Mesityl-2-(*o*-nosylamino)-propane (1f)

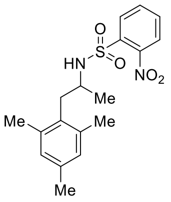

1f

^{1}HNMR (0-9 ppm)

$CDCl_3$, 400 MHz

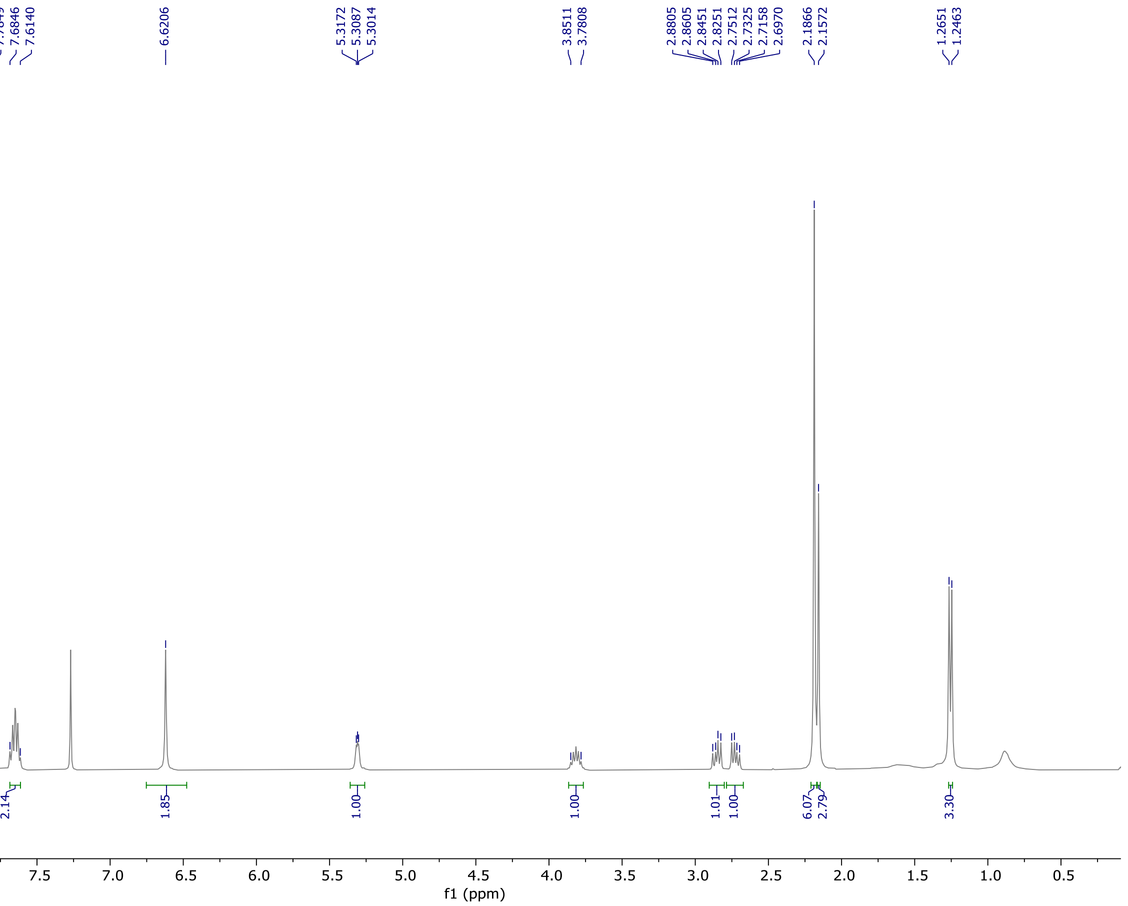

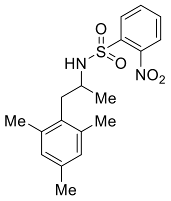

1f

$^{13}C\{^1H\}$ NMR (0-200 ppm)

$CDCl_3$, 101 MHz

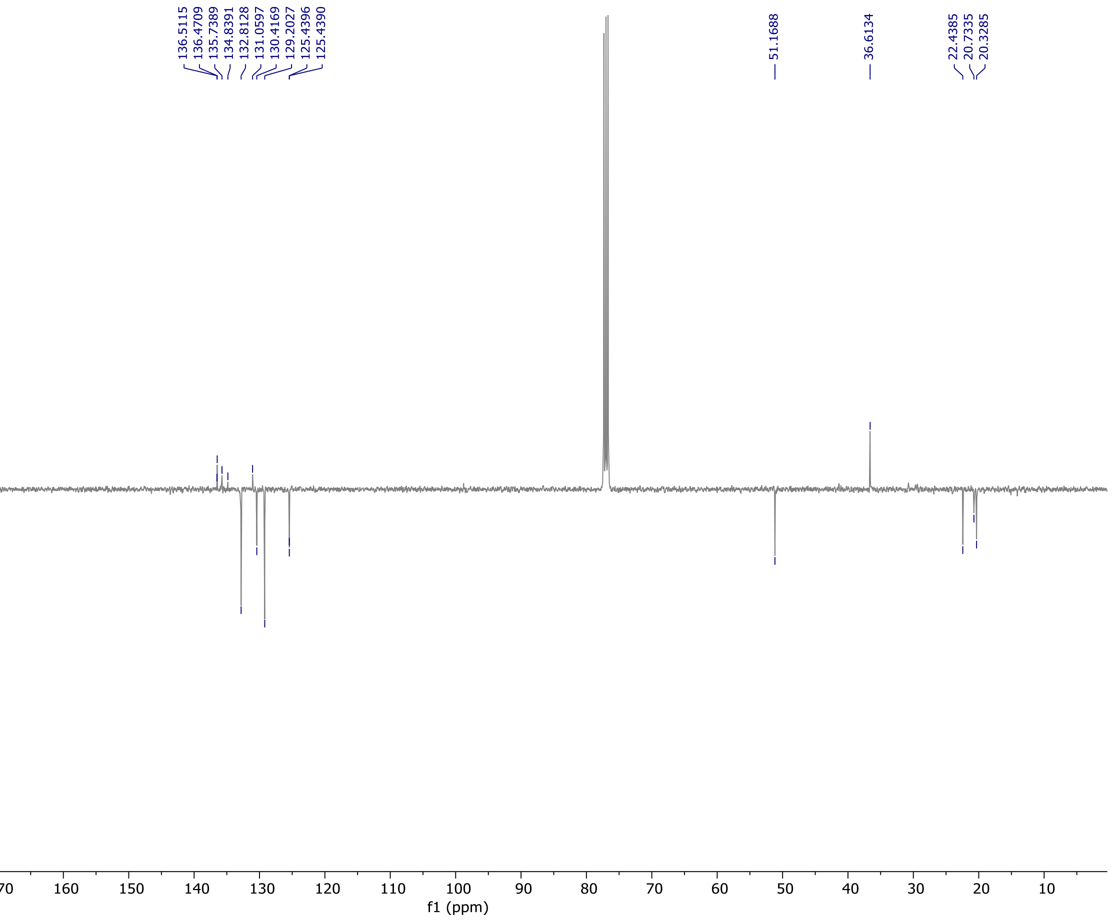

1-(2,4,6-Trimethylphenyl)-2-(2-methylbenzenesulfonamindo)-propane (1g)

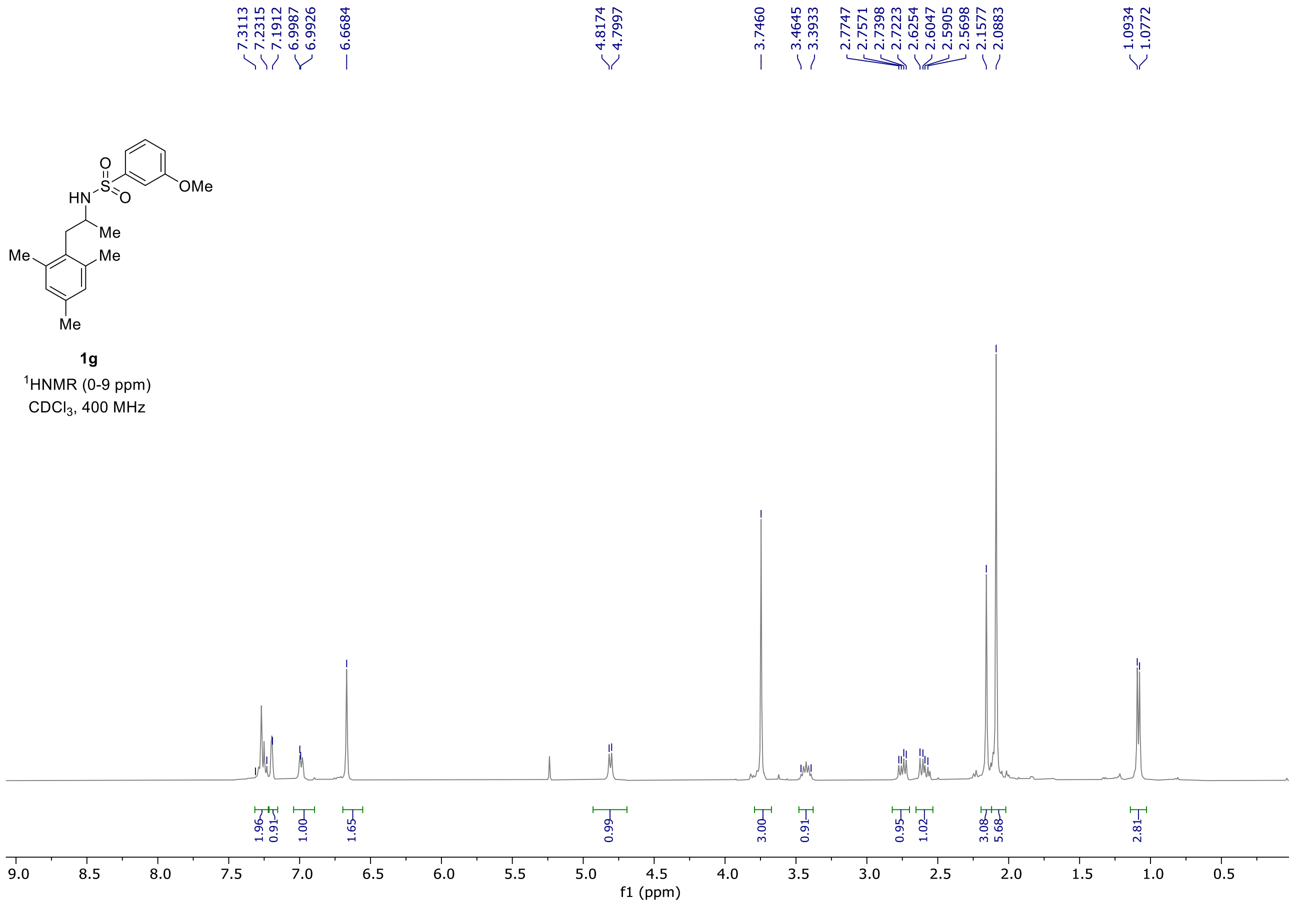

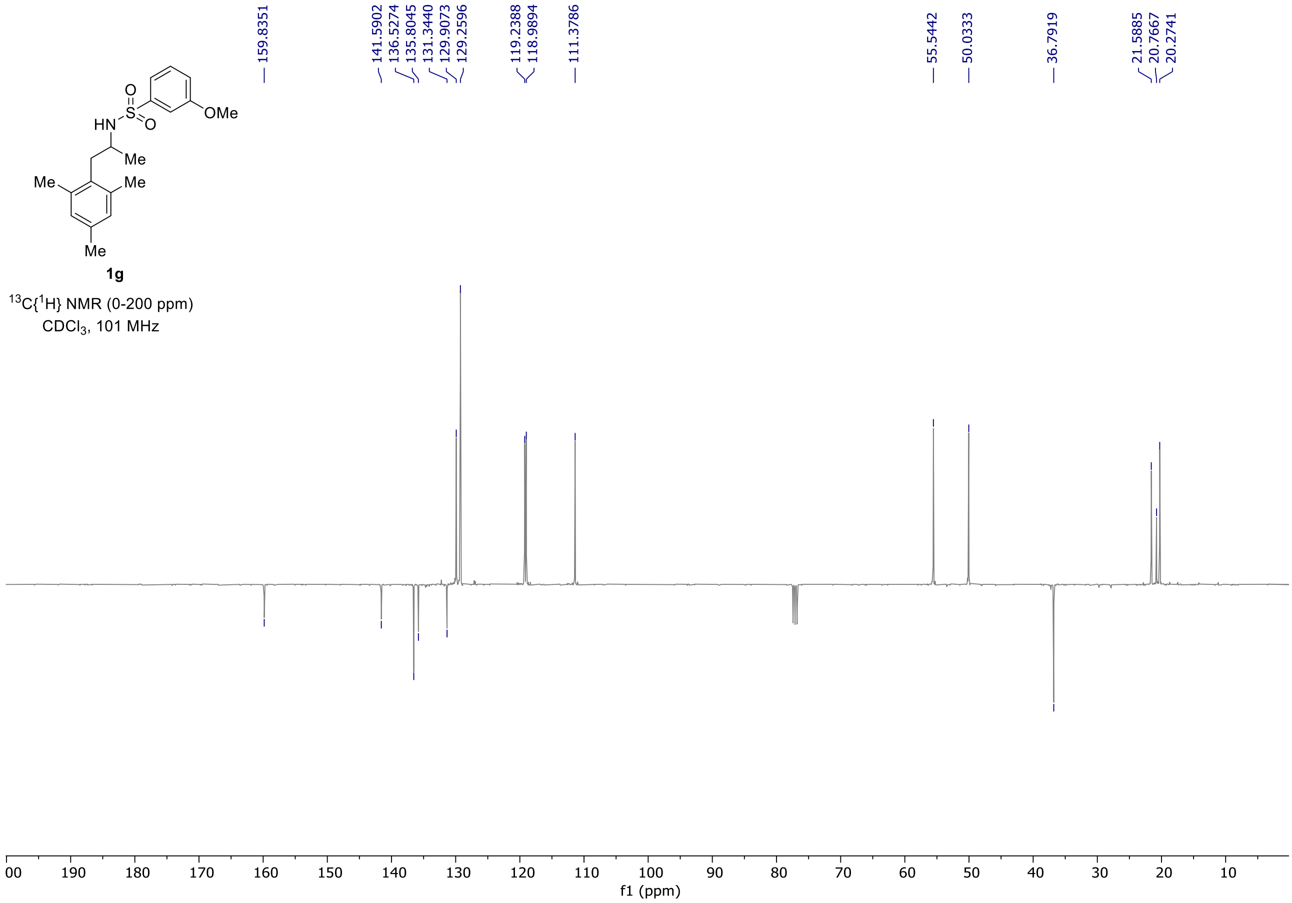

1-(2,4,6-Trimethylphenyl)-2- (benzenesulfonamindo)-propane (1h)

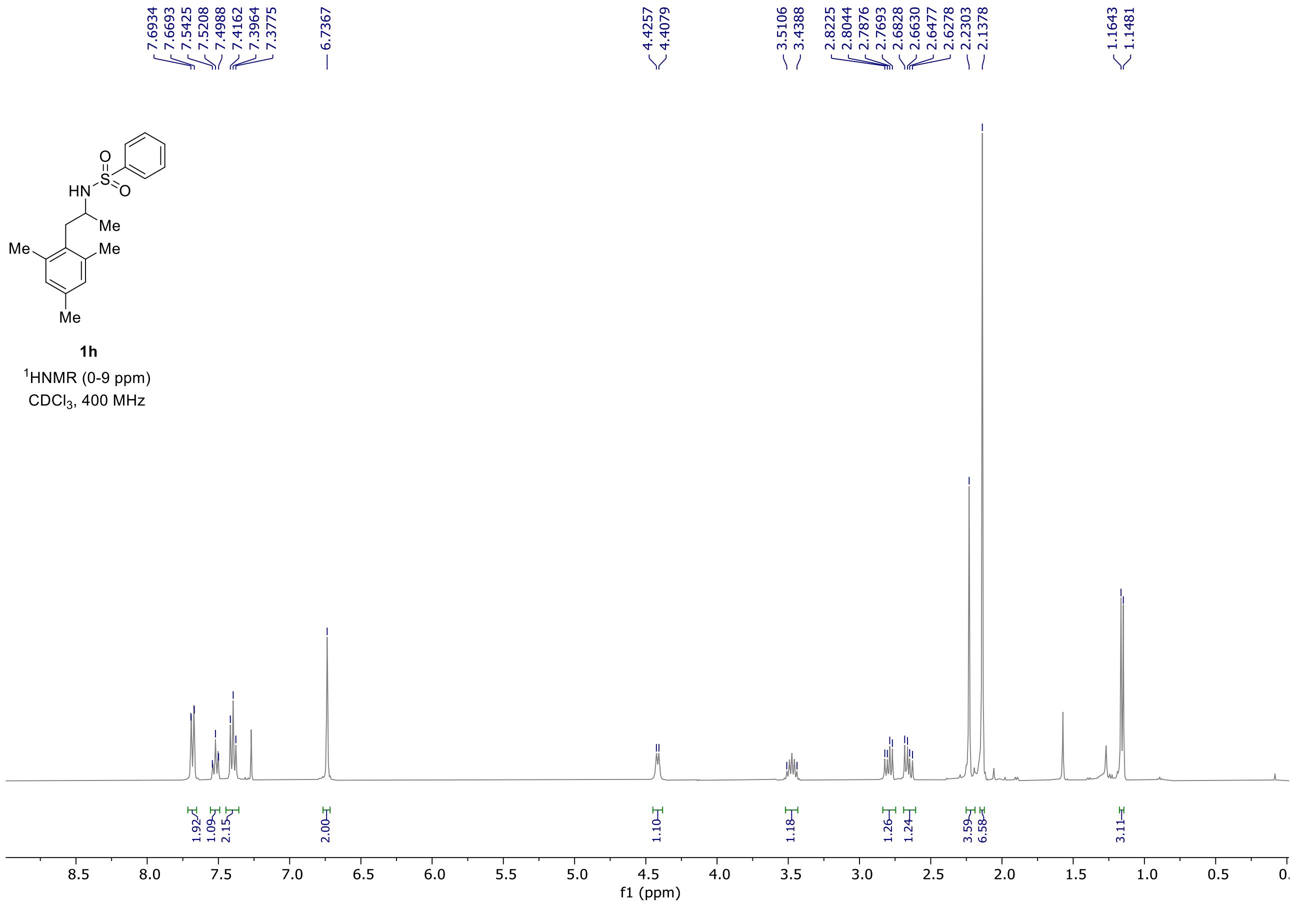

1h

^{1}HNMR (0-9 ppm)

$CDCl_3$, 400 MHz

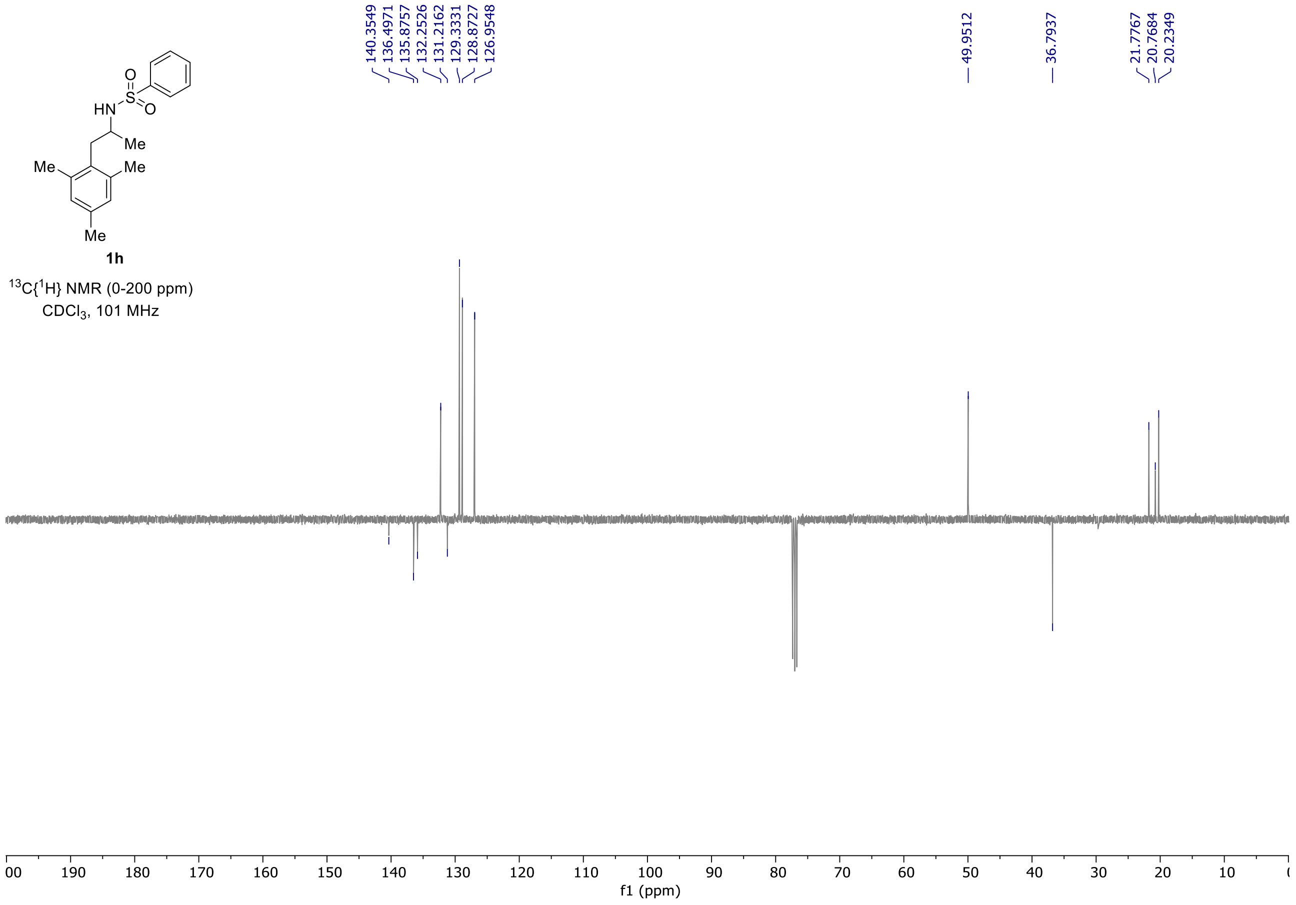

1h

$^{13}C\{^{1}H\}$ NMR (0-200 ppm)

$CDCl_3$, 101 MHz

1-(2,3,5,6-Tetramethylphenyl)-2-tosylamino-propane (3a)

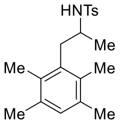

3a

^{1}HNMR (0-9 ppm)

$CDCl_3$, 400 MHz

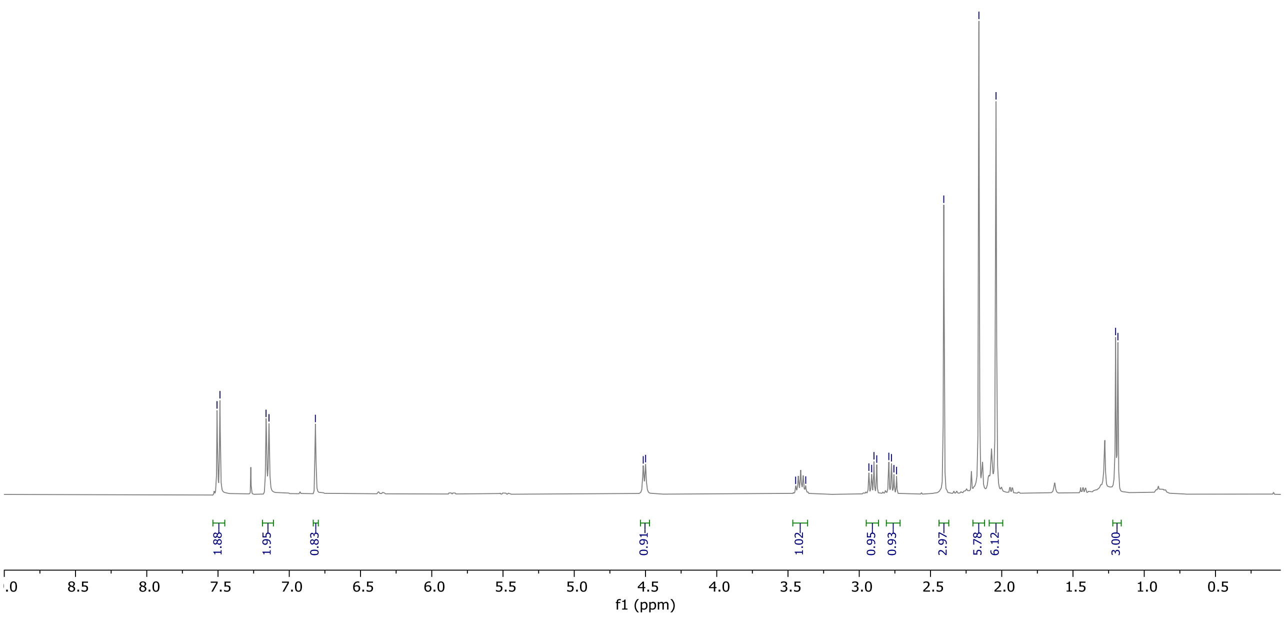

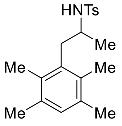

3a

$^{13}C\{^{1}H\}$ NMR (0-200 ppm)

$CDCl_3$, 101 MHz

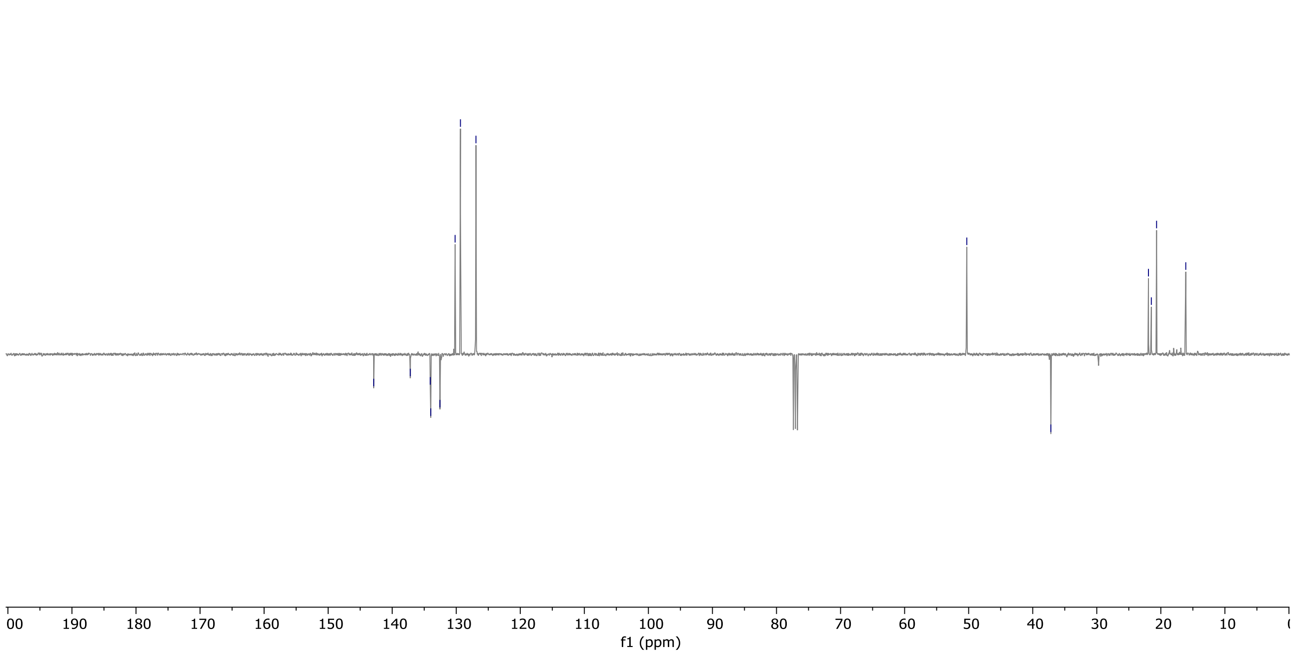

1-(2,3,5,6-Tetramethylphenyl)-2-(benzenesulfonamido)-propane (3b)

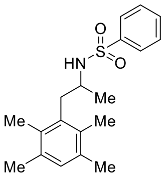

3b

^{1}HNMR (0-9 ppm)

$CDCl_3$, 400 MHz

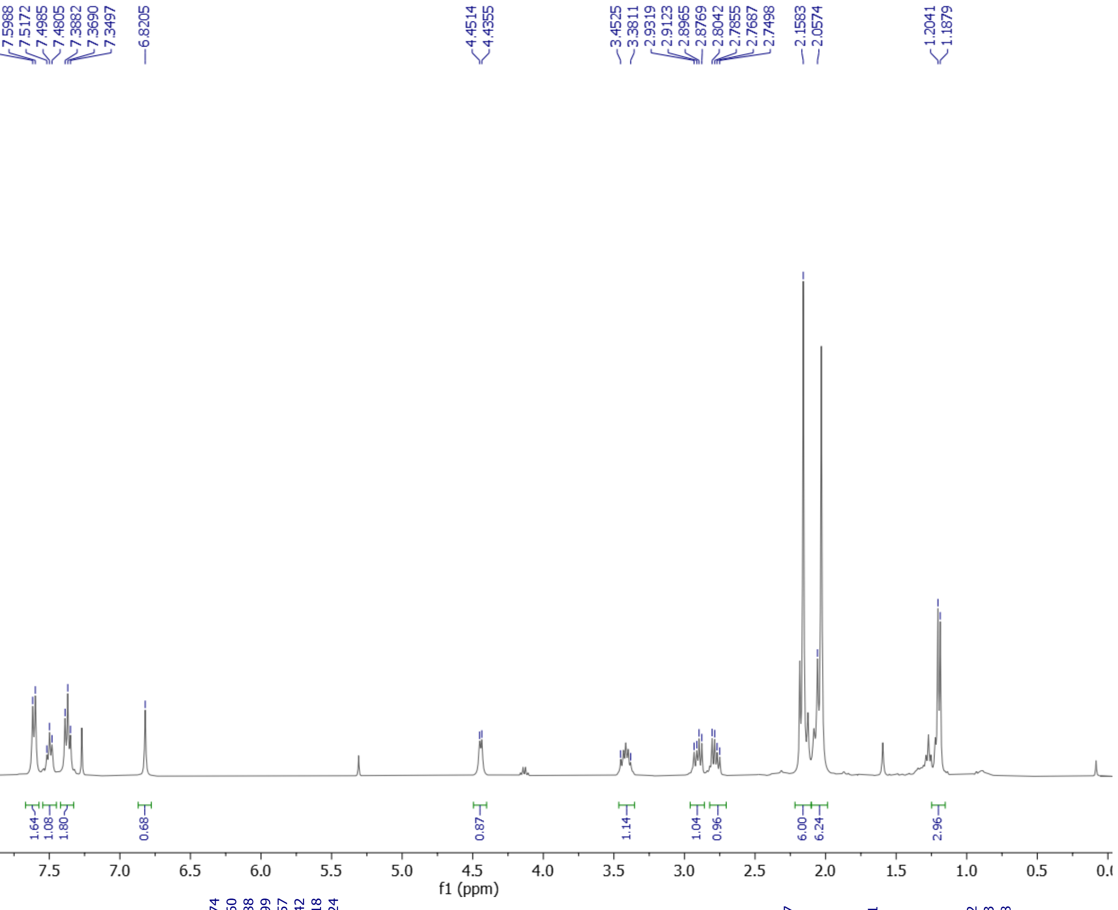

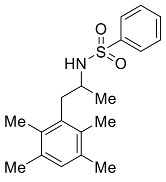

3b

^{13}C{^{1}H} NMR (0-200 ppm)

$CDCl_3$, 101 MHz

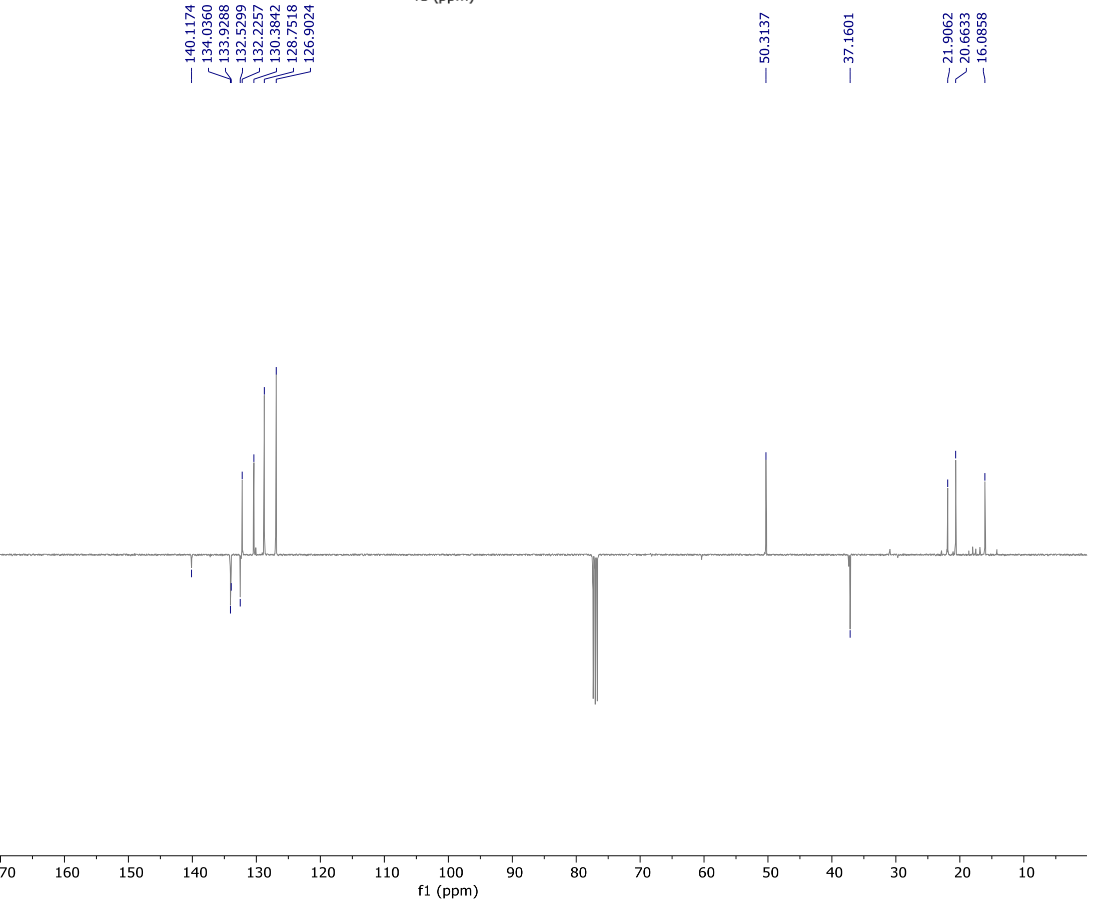

1-(2,3,5,6-Tetramethylphenyl)-2-(4-chlorobenzenesulfonamido)-propane (3c)

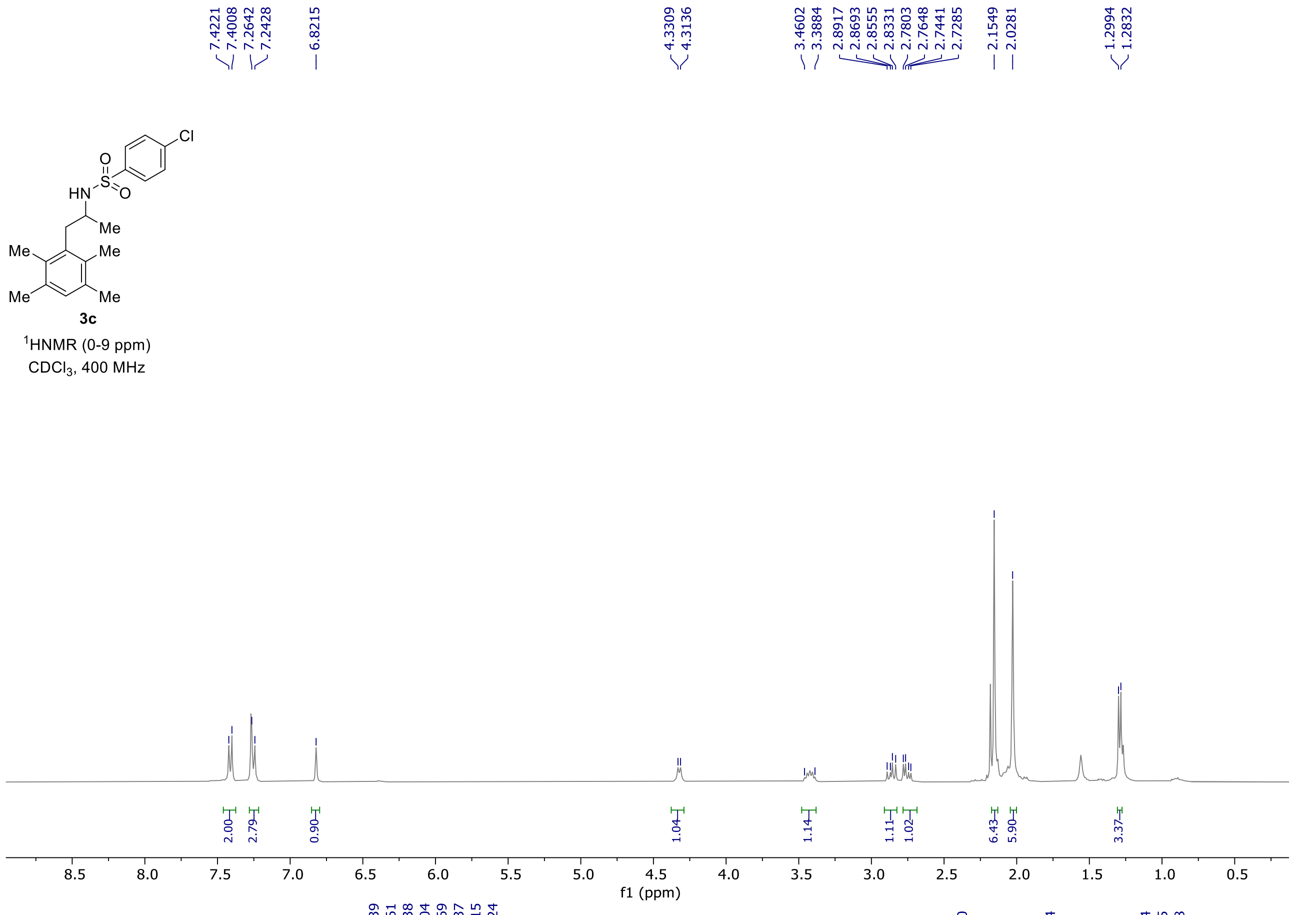

3c

^{1}HNMR (0-9 ppm)

$CDCl_3$, 400 MHz

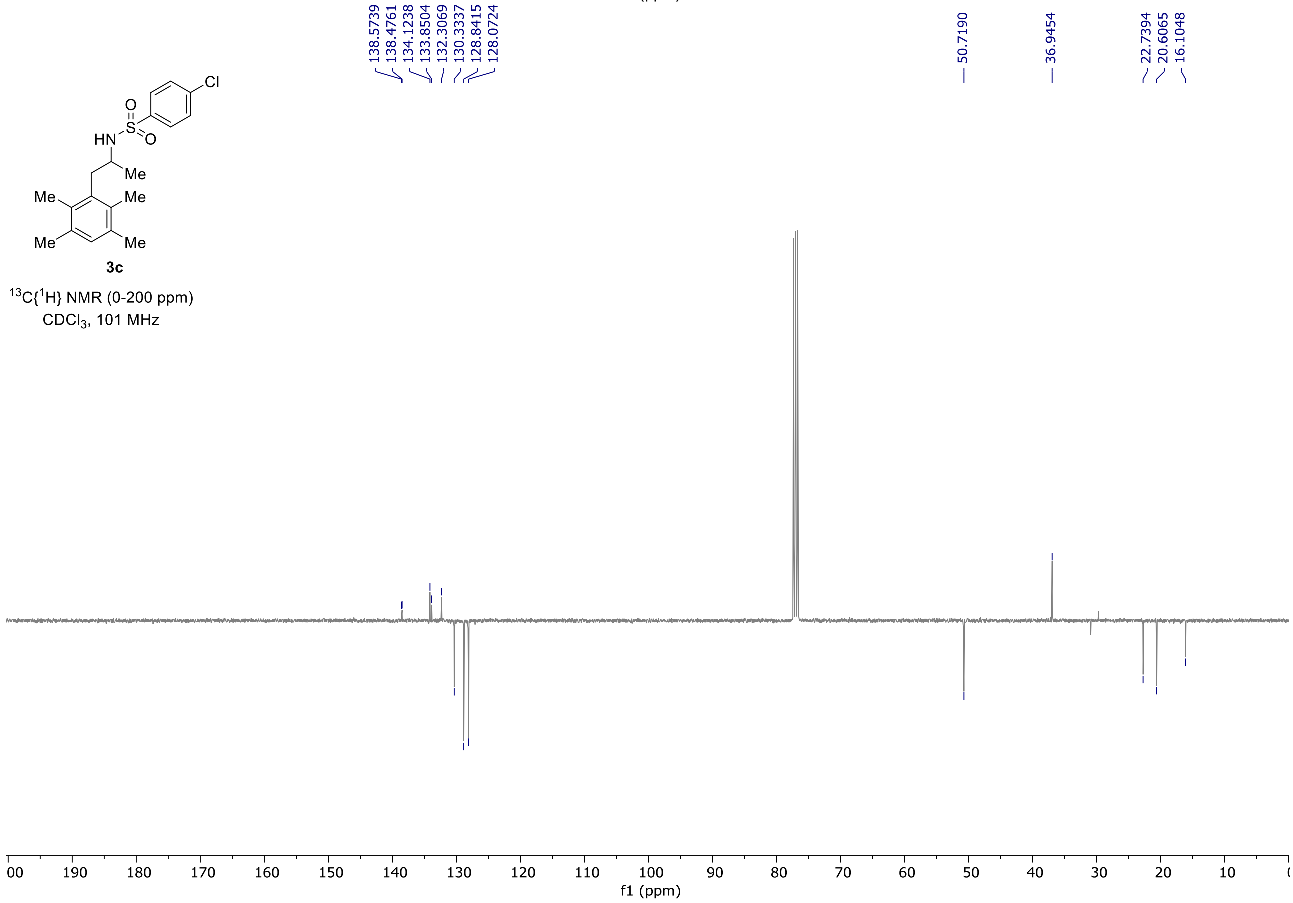

3c

$^{13}C\{^{1}H\}$ NMR (0-200 ppm)

$CDCl_3$, 101 MHz

1-(2,3,5,6-Tetramethylphenyl)-2-(*p*-nosylamino)-propane (3d)

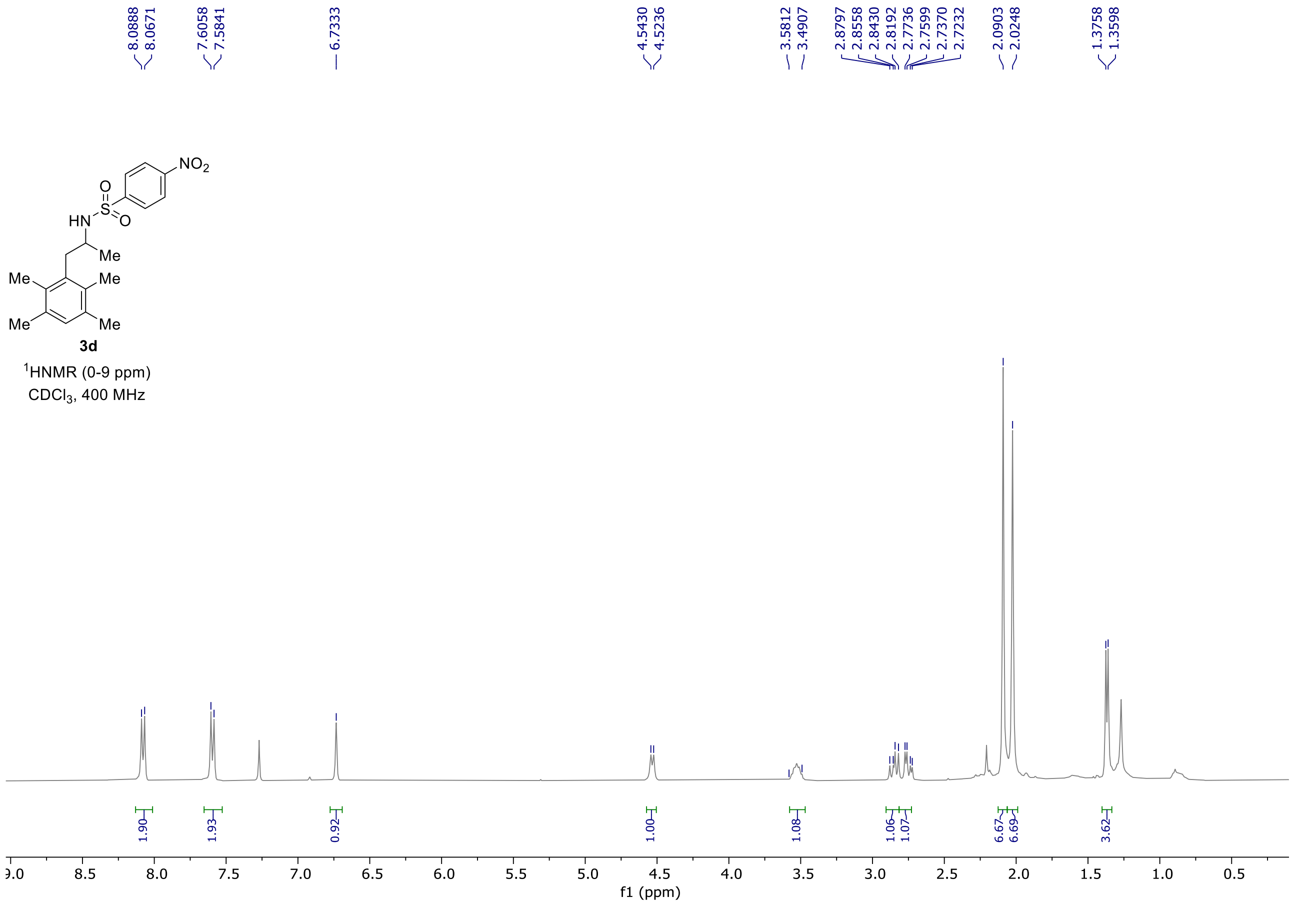

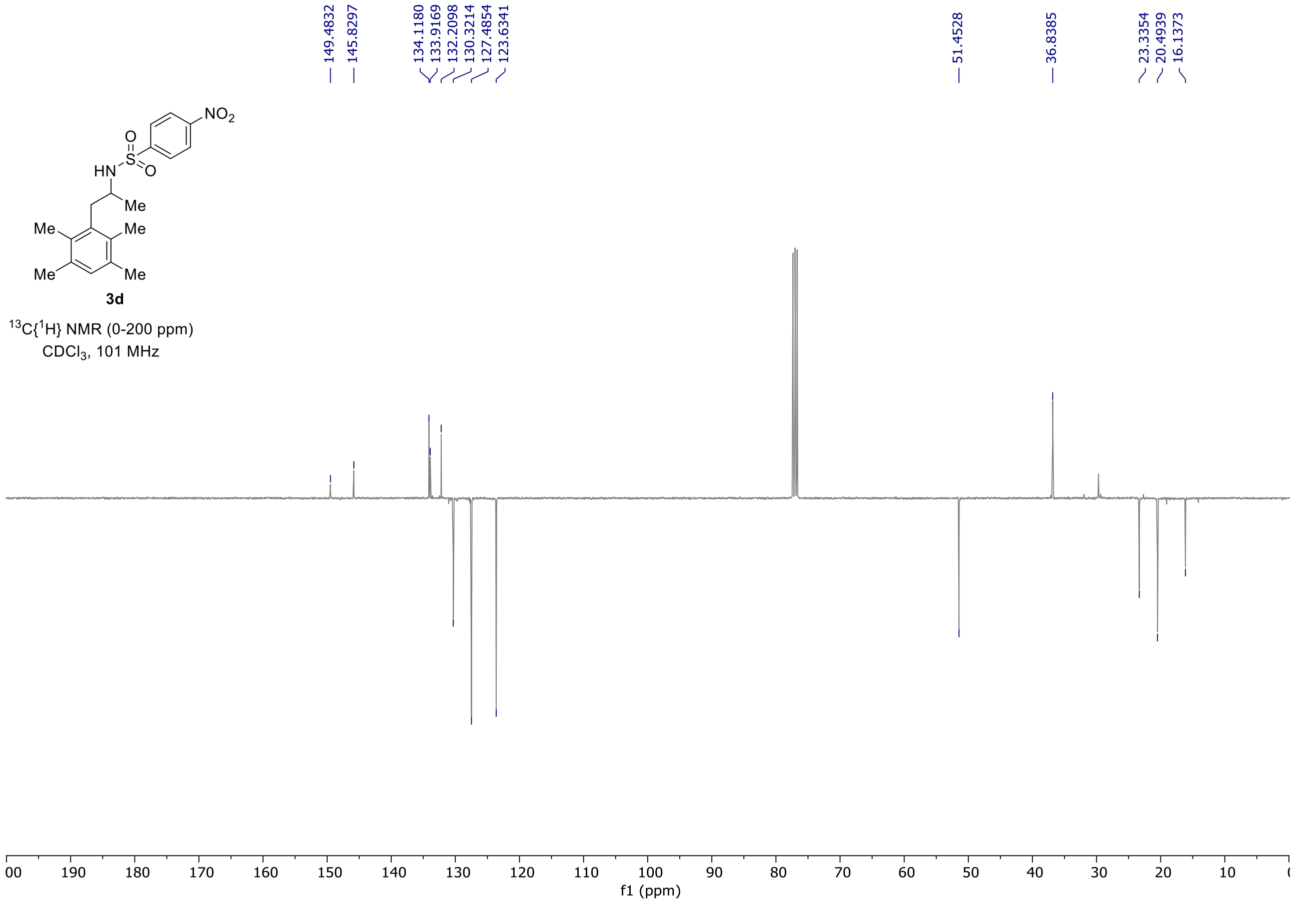

1-(2,3,5,6-Tetramethylphenyl)-2-(2-methylbenzenesulfonamido)-propane (3e)

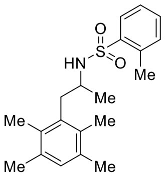

3e

^{1}HNMR (0-9 ppm)

$CDCl_3$, 400 MHz

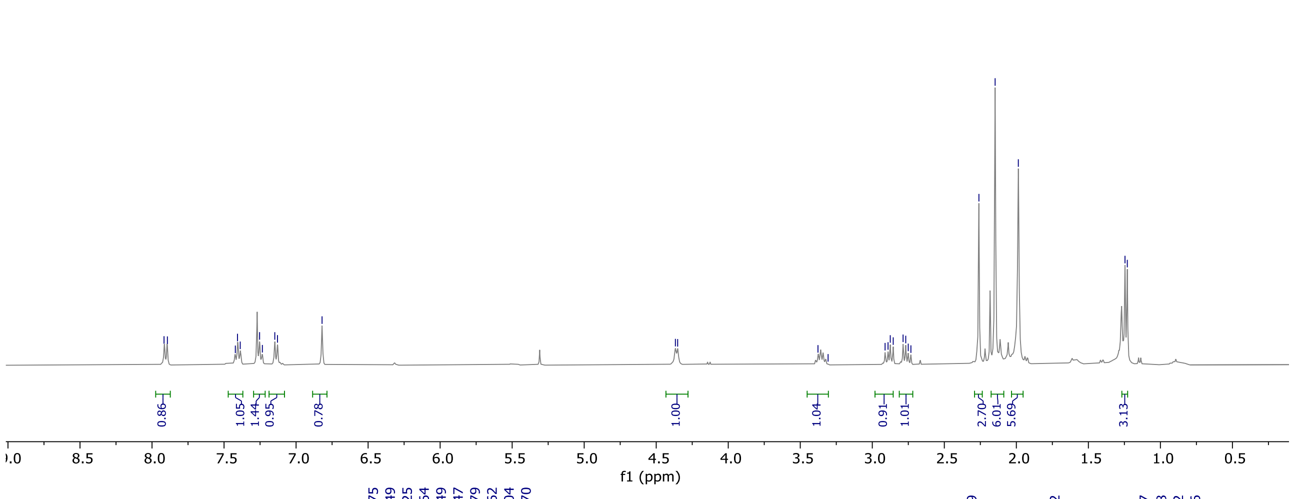

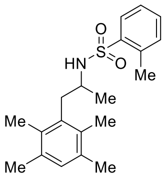

3e

$^{13}C\{^{1}H\}$ NMR (0-200 ppm)

$CDCl_3$, 101 MHz

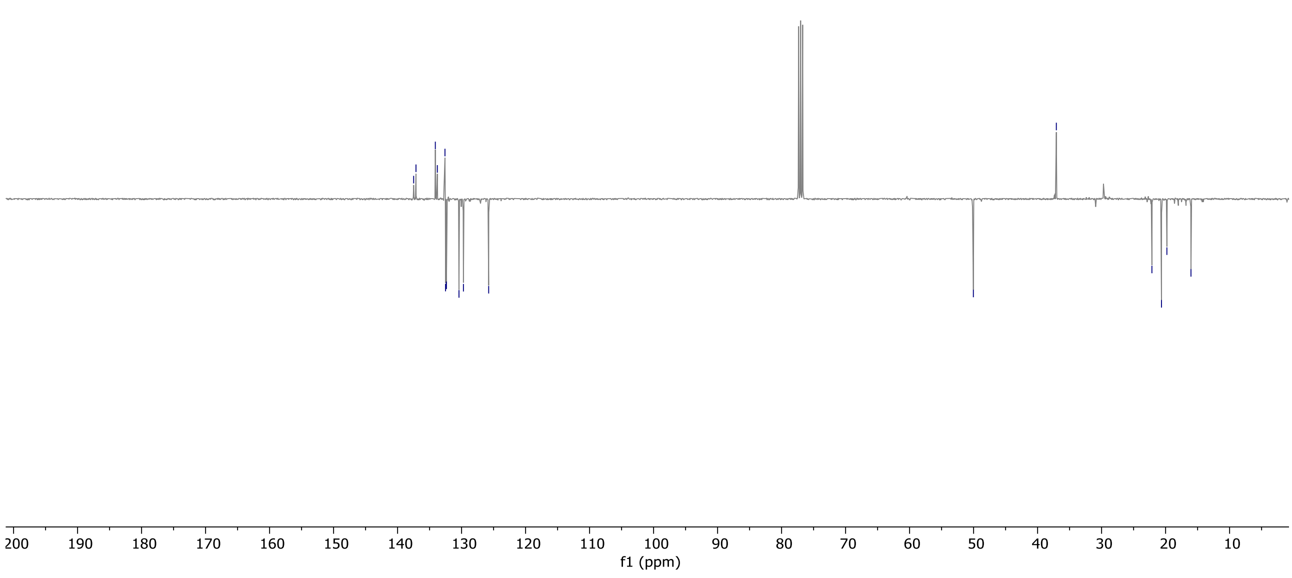

1-(2,3,5,6-Tetramethylphenyl)-2-(*o*-nosylamino)-propane (3f)

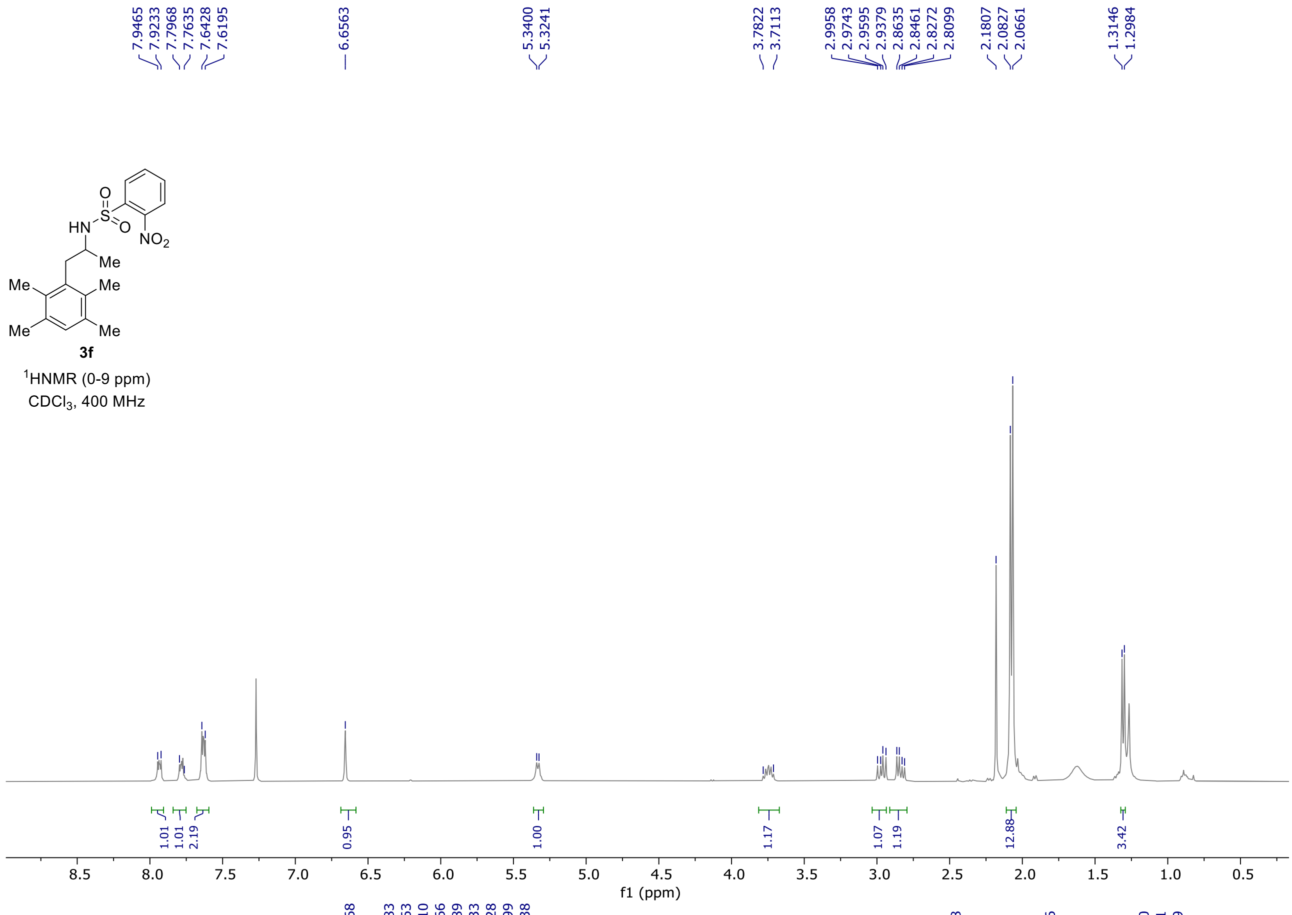

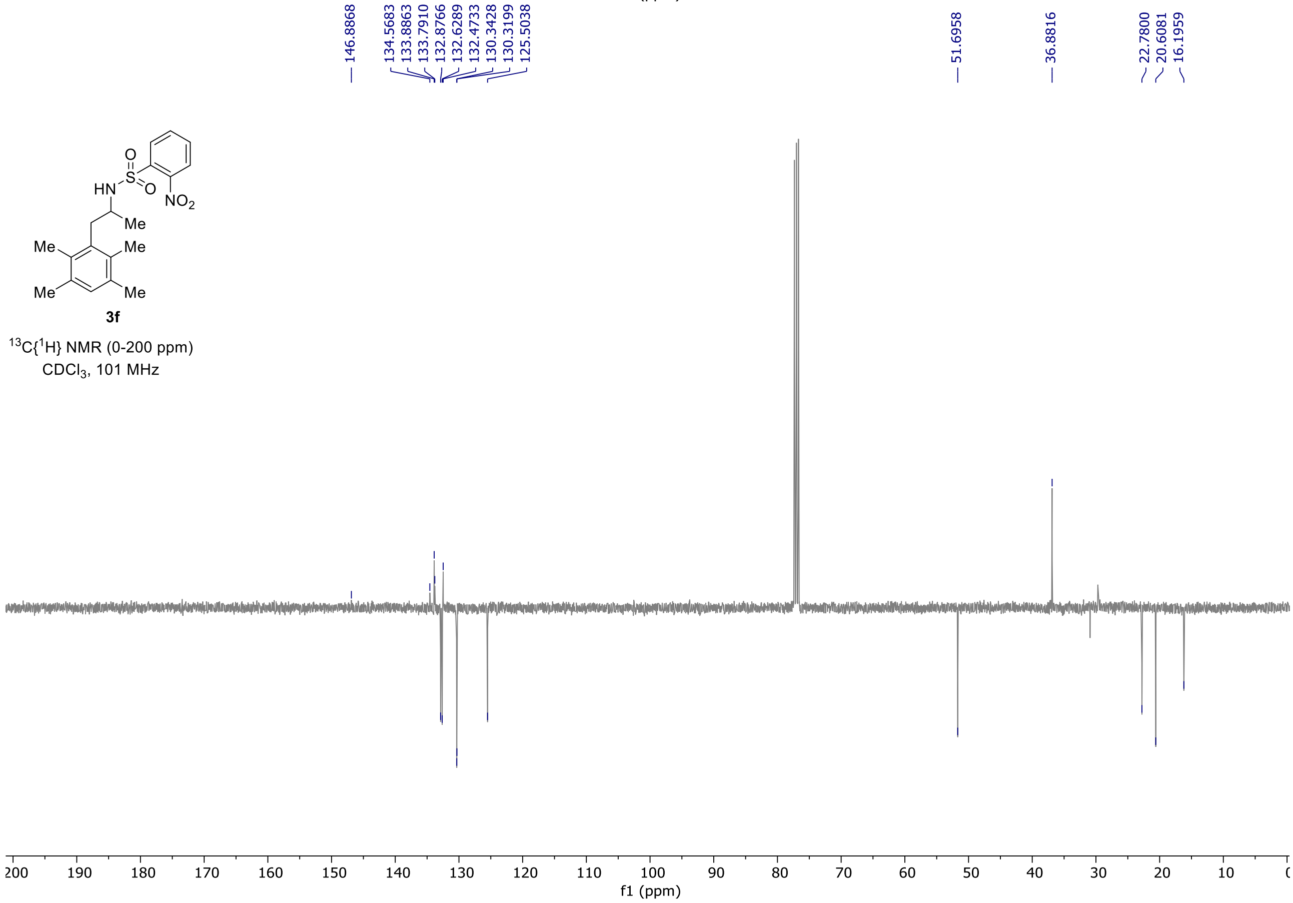

1-(2,3,4,5,6-Pentamethylphenyl)-2-tosylamino-propane (4a)

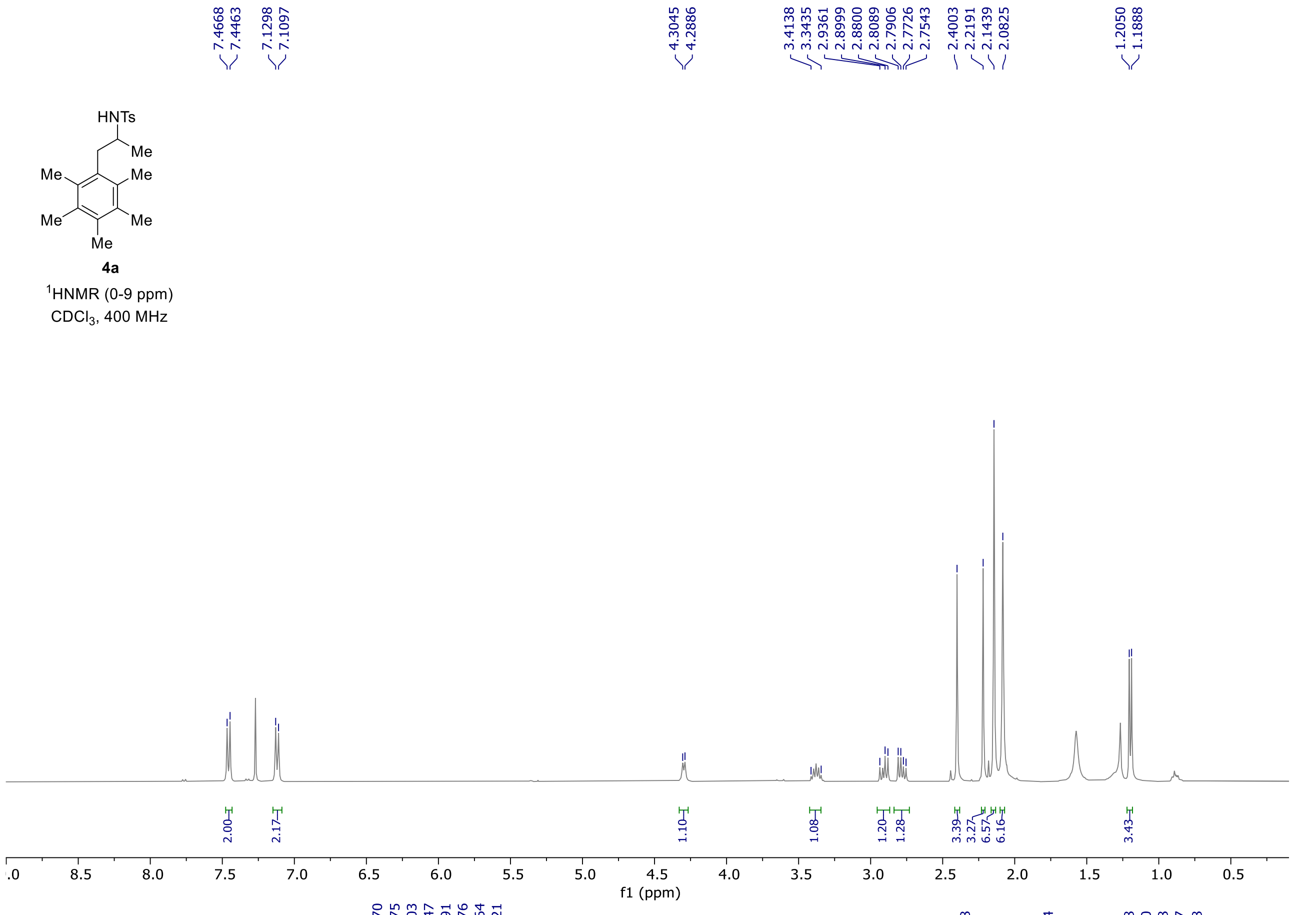

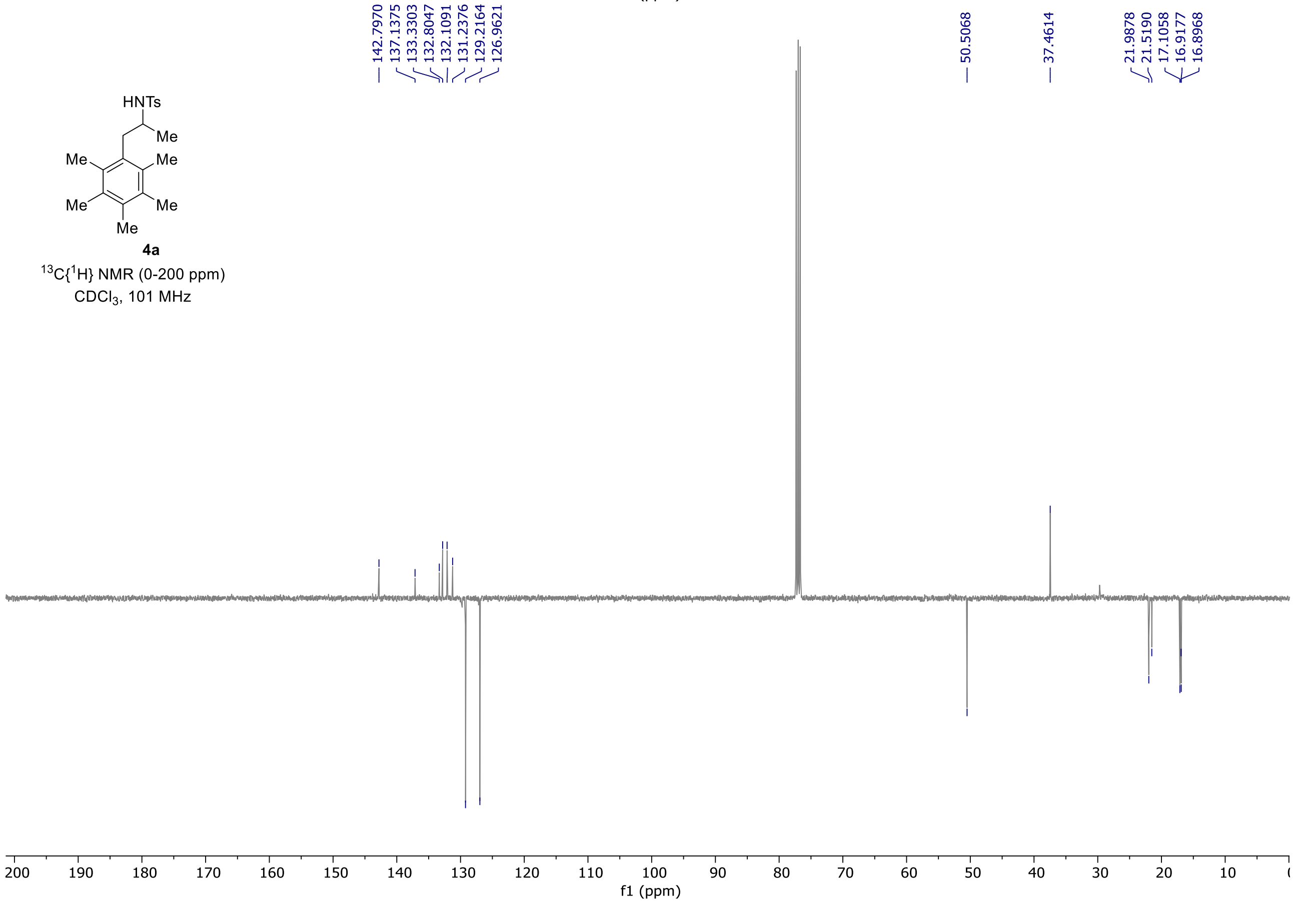

1-(2,3,4,5,6-Pentamethylphenyl)-2-(*p*-nosylamino)-propane (4b)

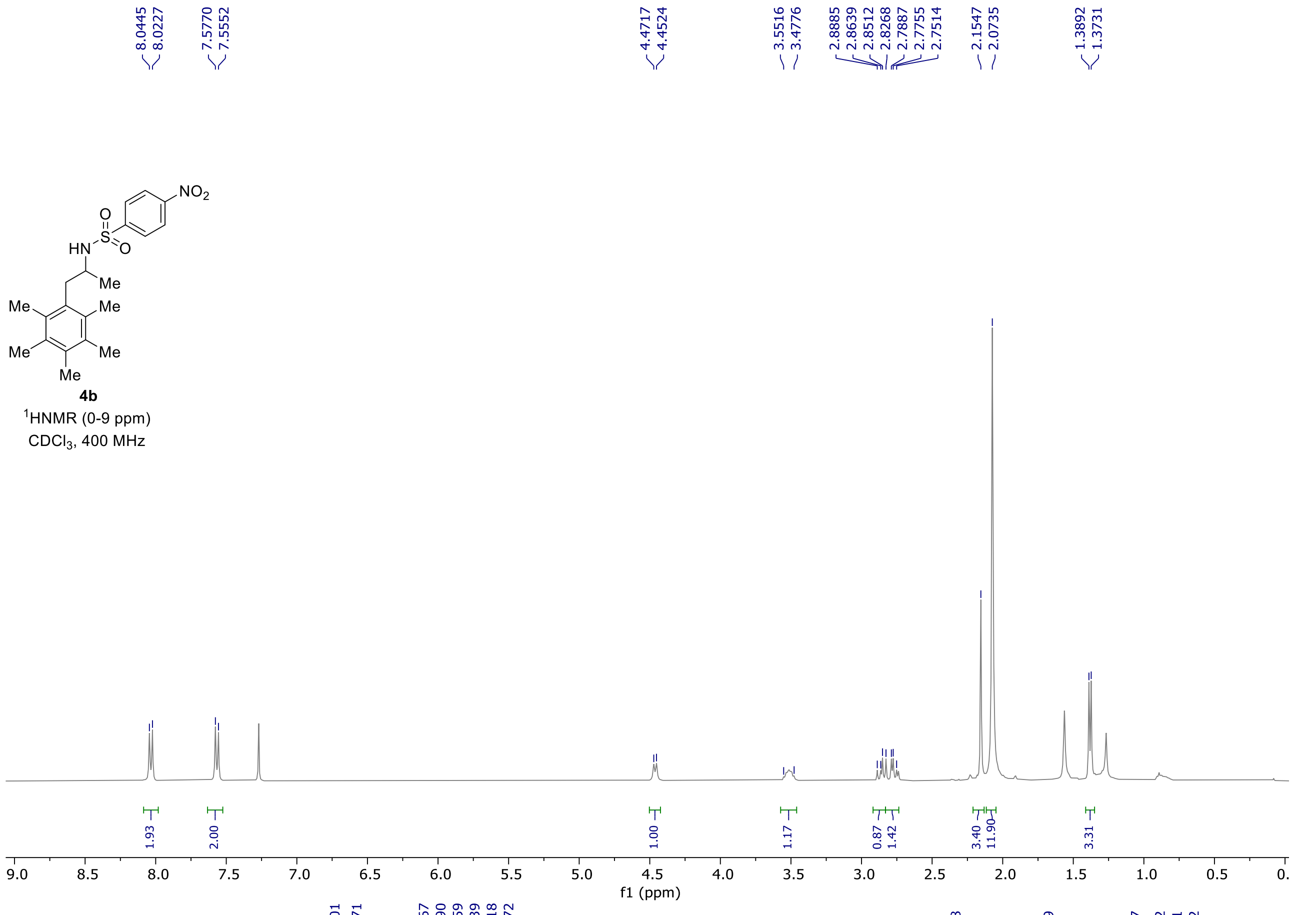

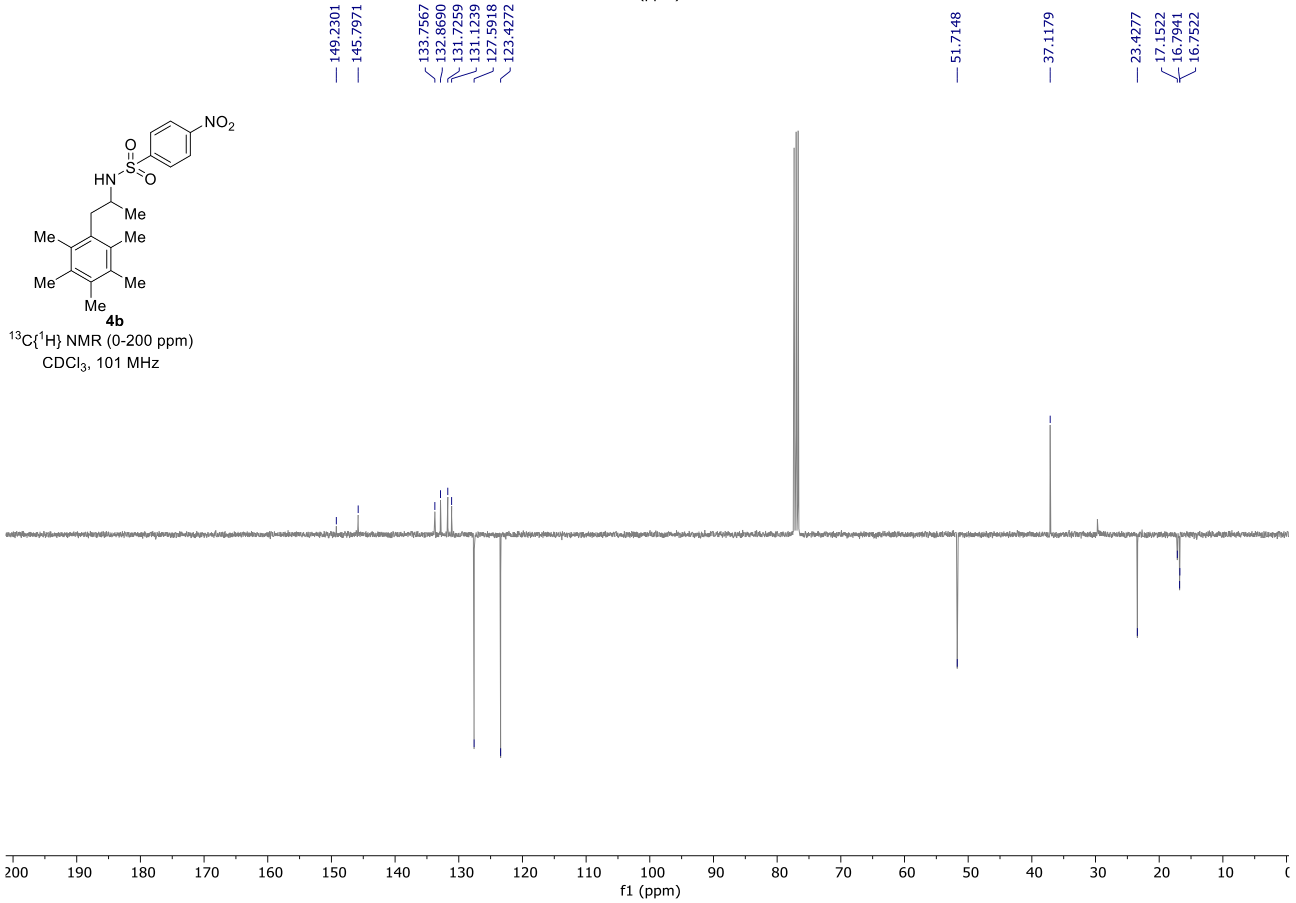

1-(2,3,4,5,6-Pentamethylphenyl)-2-(2-methylbenzenesulfonamido)-propane (4c)

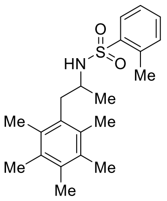

4c

^{1}HNMR (0-9 ppm)

$CDCl_3$, 400 MHz

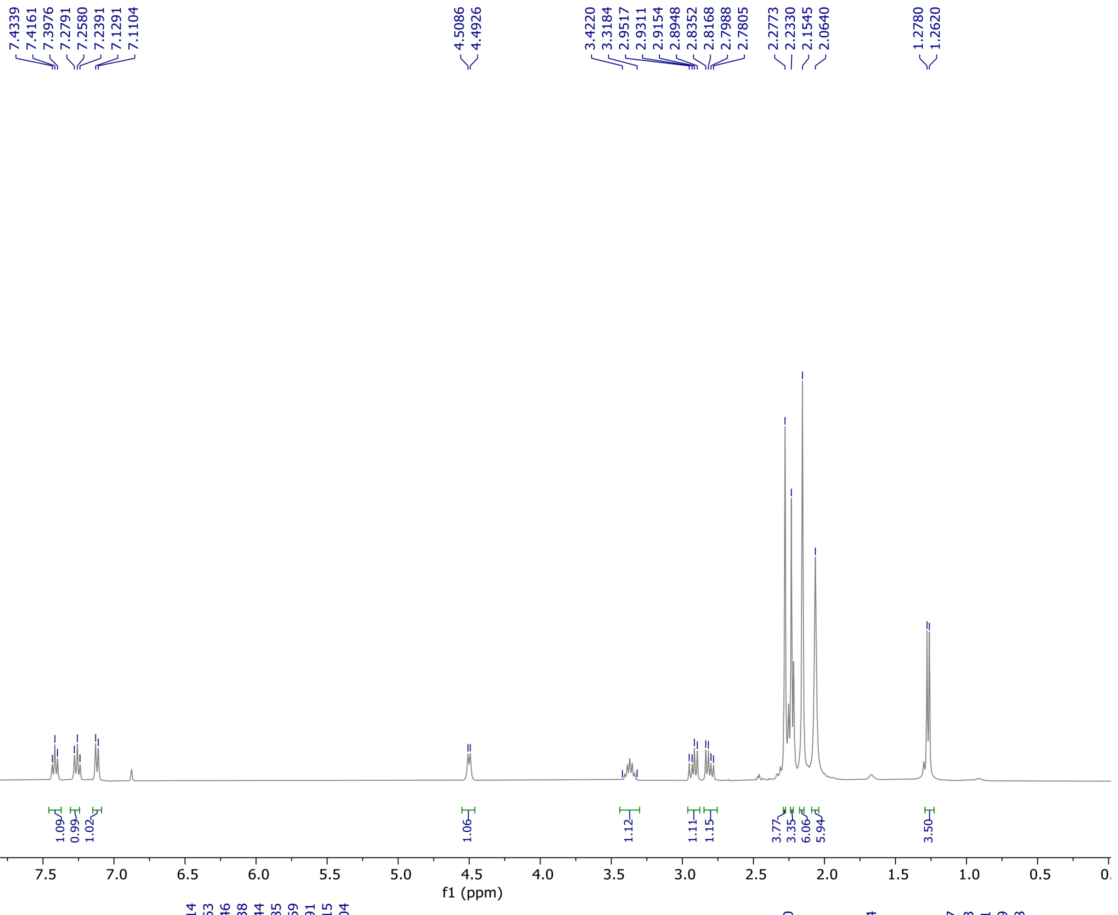

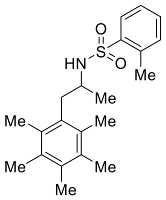

4c

$^{13}C\{^{1}H\}$ NMR (0-200 ppm)

$CDCl_3$, 101 MHz

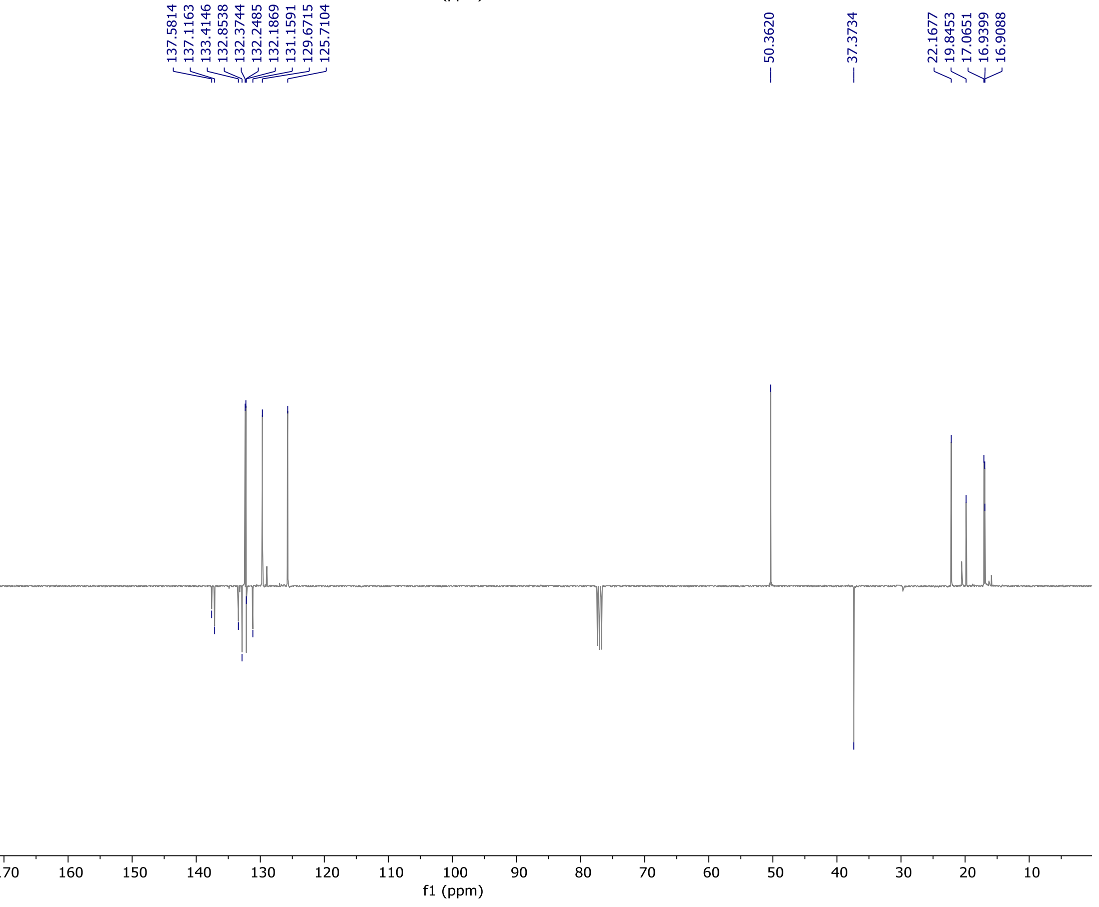

1-(2,3,4,5,6-Pentamethylphenyl)-2-(*o*-nosylamino)-propane (4d)

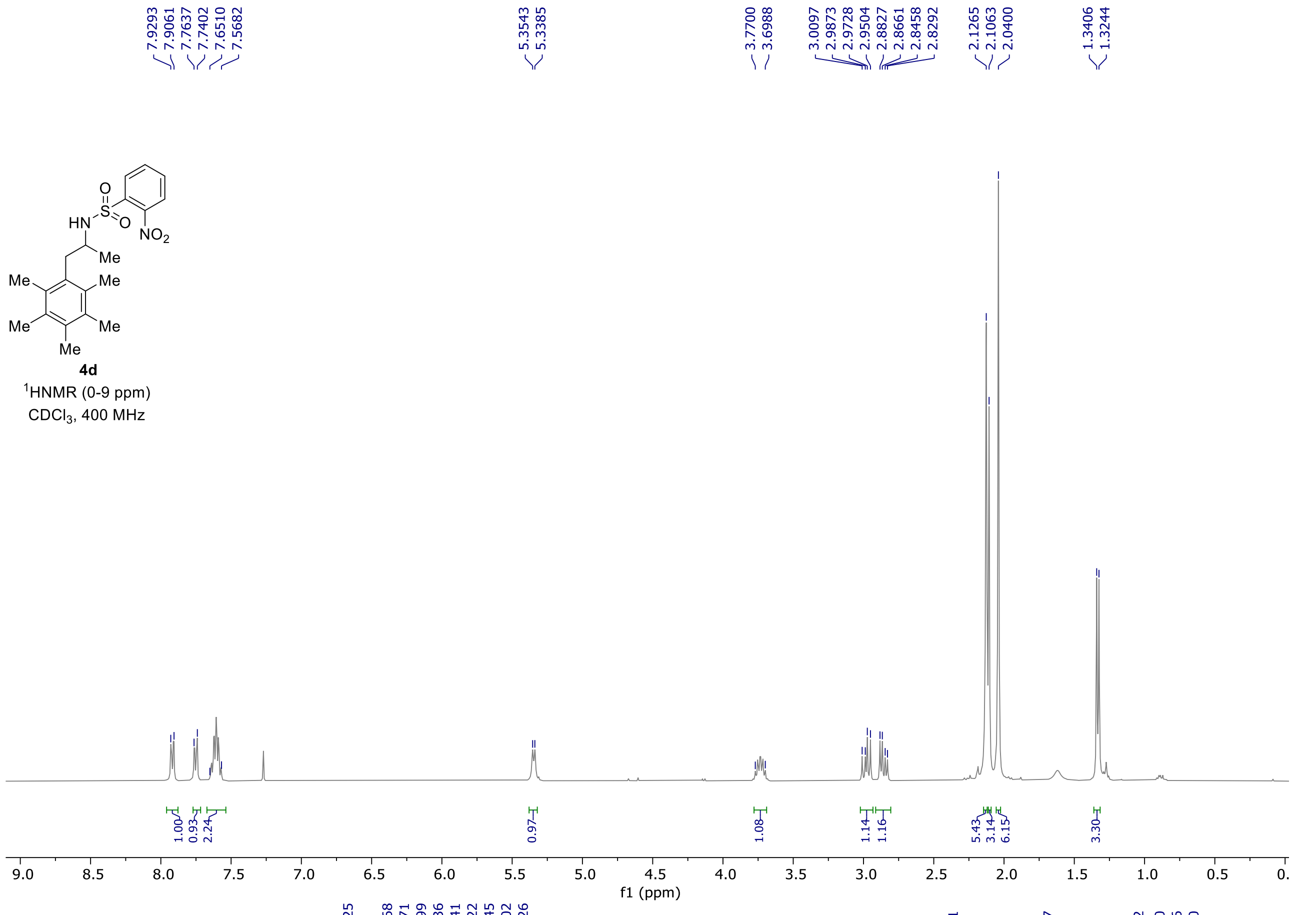

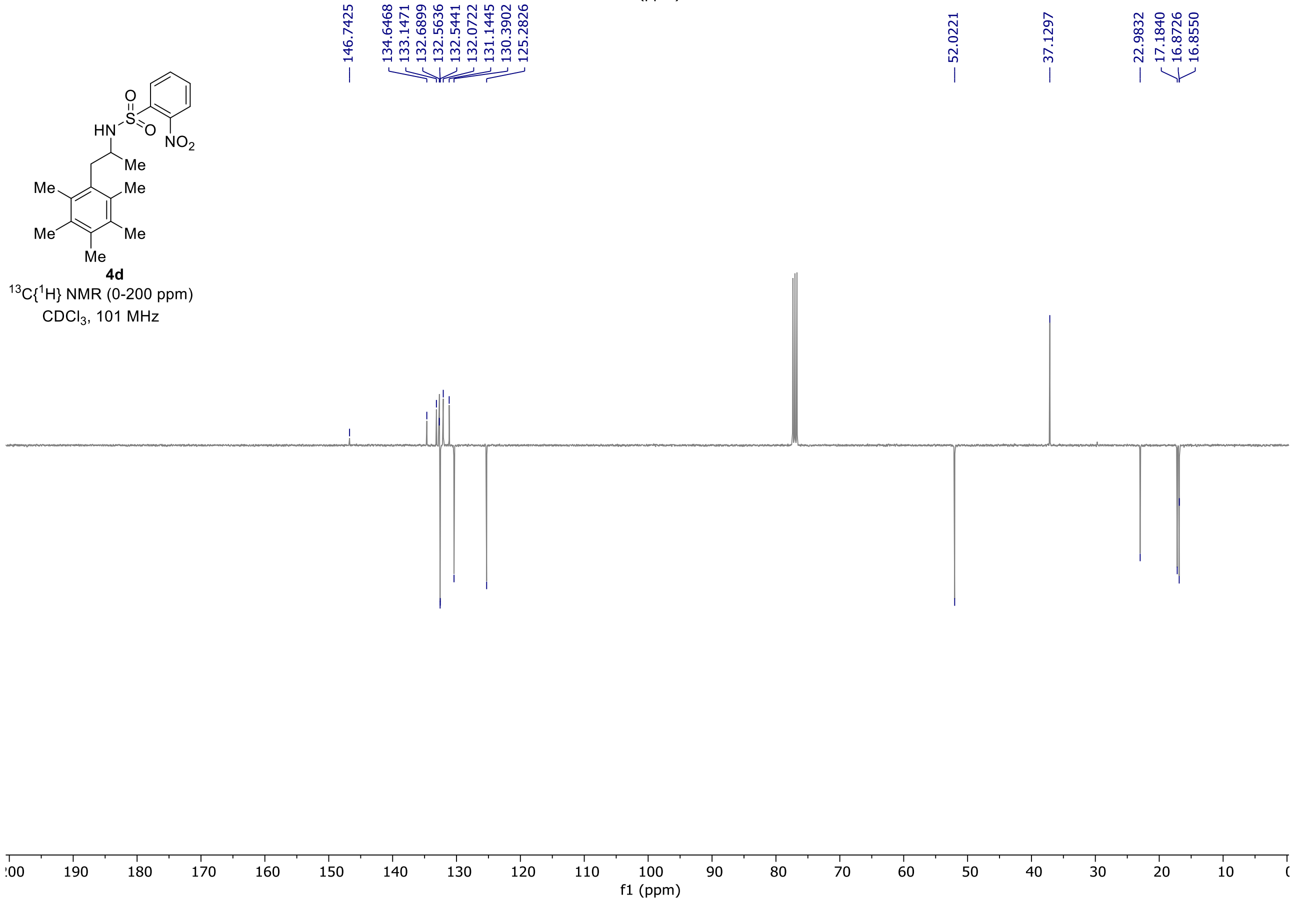

1-(2,5-Dimethylphenyl)-2-tosylamino-propane (5a)

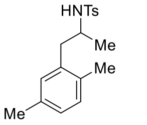

5a

^{1}HNMR (0-9 ppm)

$CDCl_3$, 400 MHz

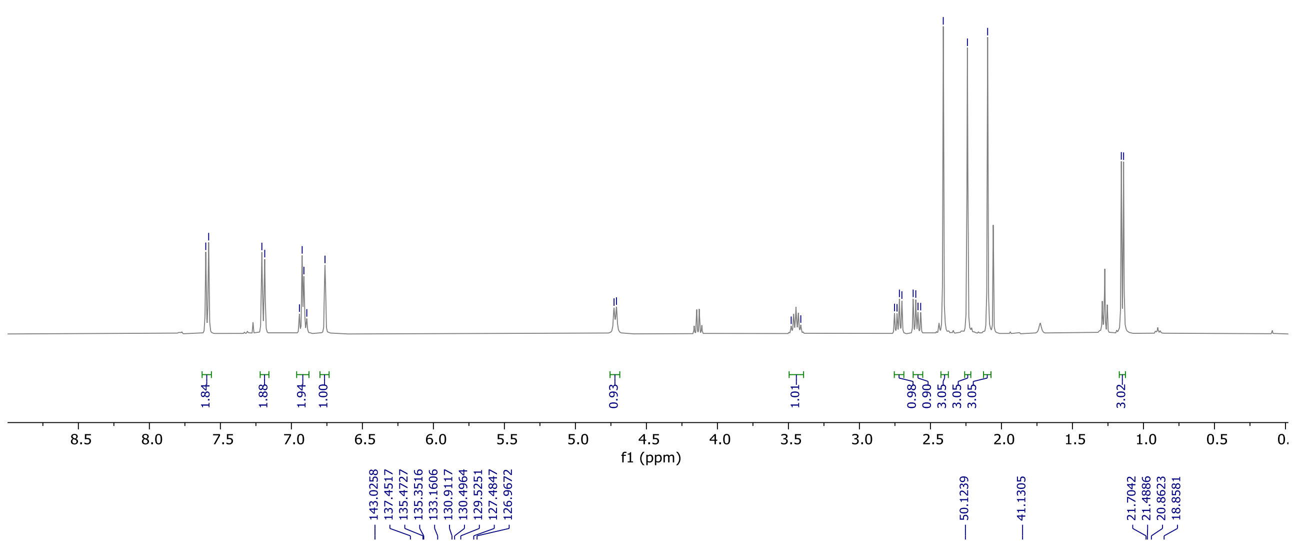

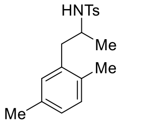

5a

$^{13}C\{^1H\}$ NMR (0-200 ppm)

$CDCl_3$, 101 MHz

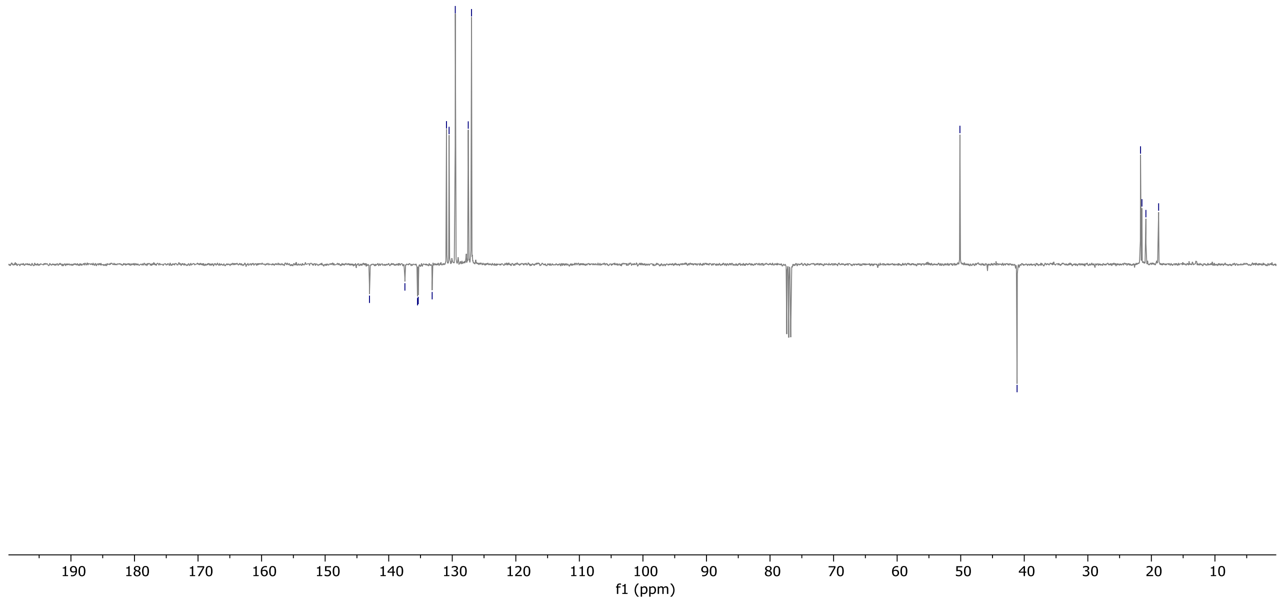

1-(2,5-Dimethylphenyl)-2-(*p*-nosylamino)-propane (5b)

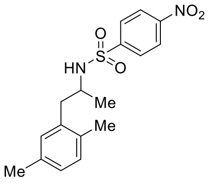

5b
^{1}HNMR (0-9 ppm)
$CDCl_3$, 400 MHz

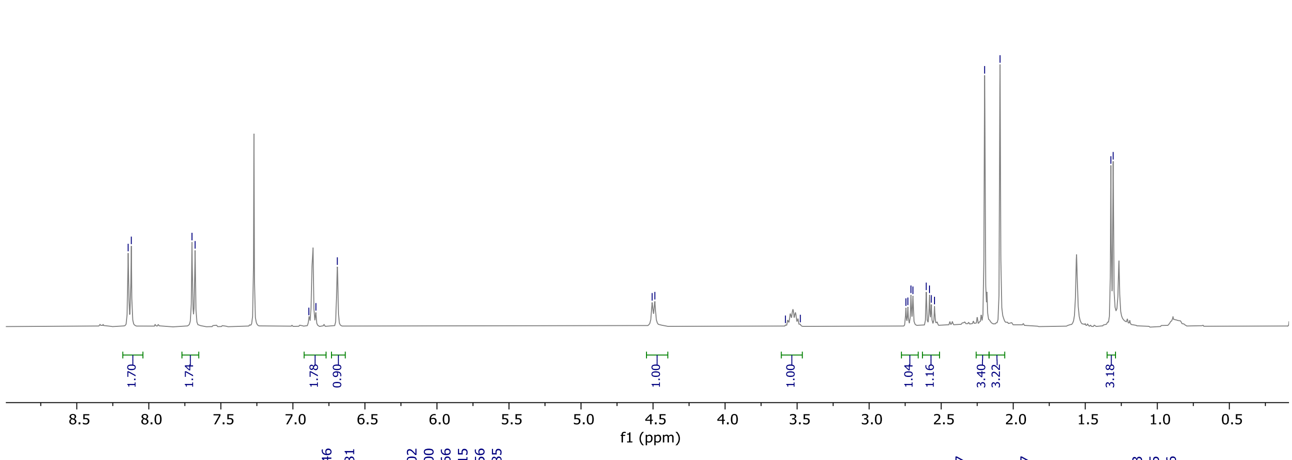

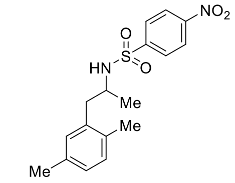

5b
$^{13}C\{^{1}H\}$ NMR (0-200 ppm)
$CDCl_3$, 101 MHz

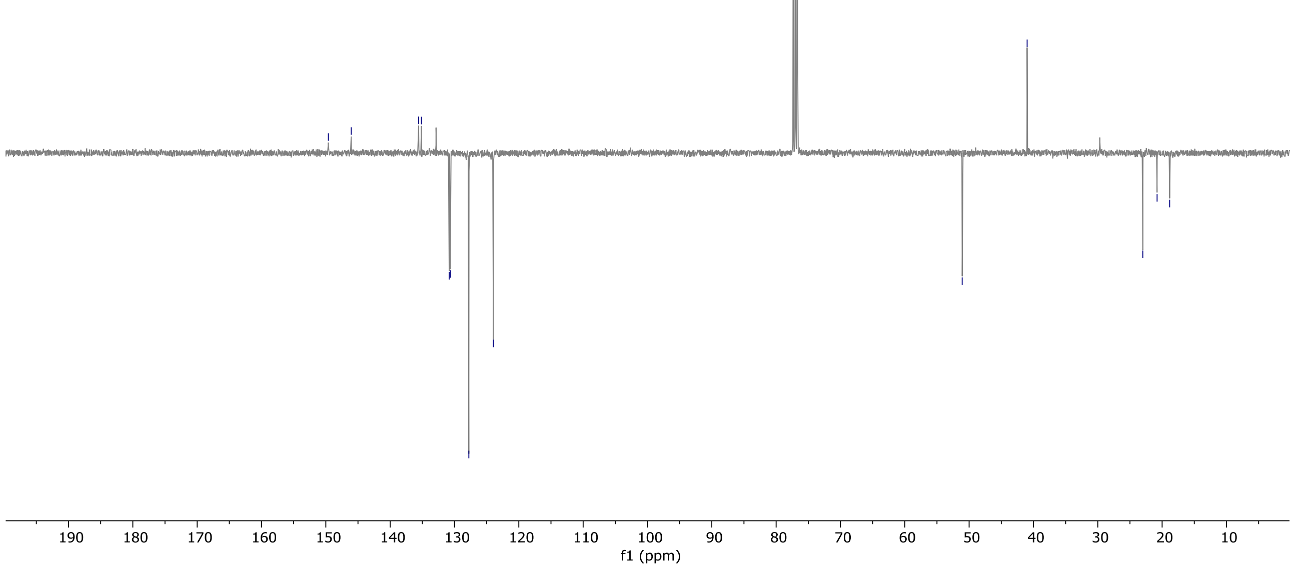

1-(2,5-Dimethylphenyl)-2-(4-chlorobenzenesulfonamido)-propane (5c)

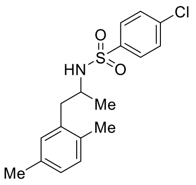

5c

^{1}HNMR (0-9 ppm)

$CDCl_3$, 400 MHz

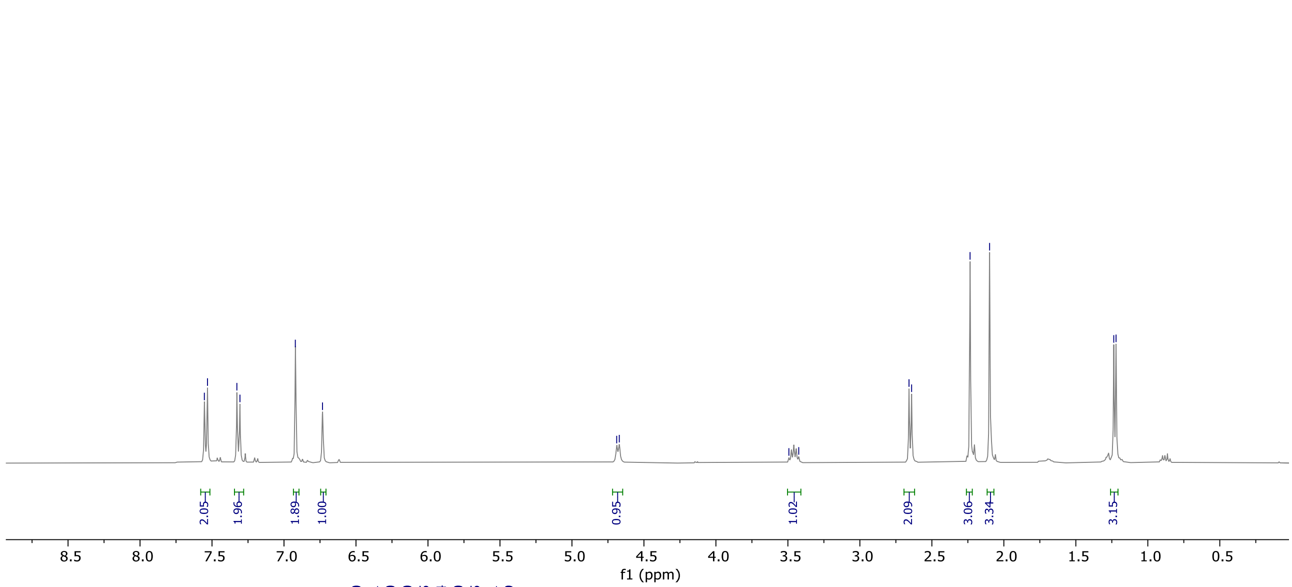

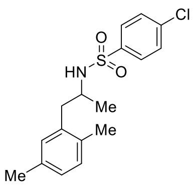

5c

$^{13}C\{^{1}H\}$ NMR (0-200 ppm)

$CDCl_3$, 101 MHz

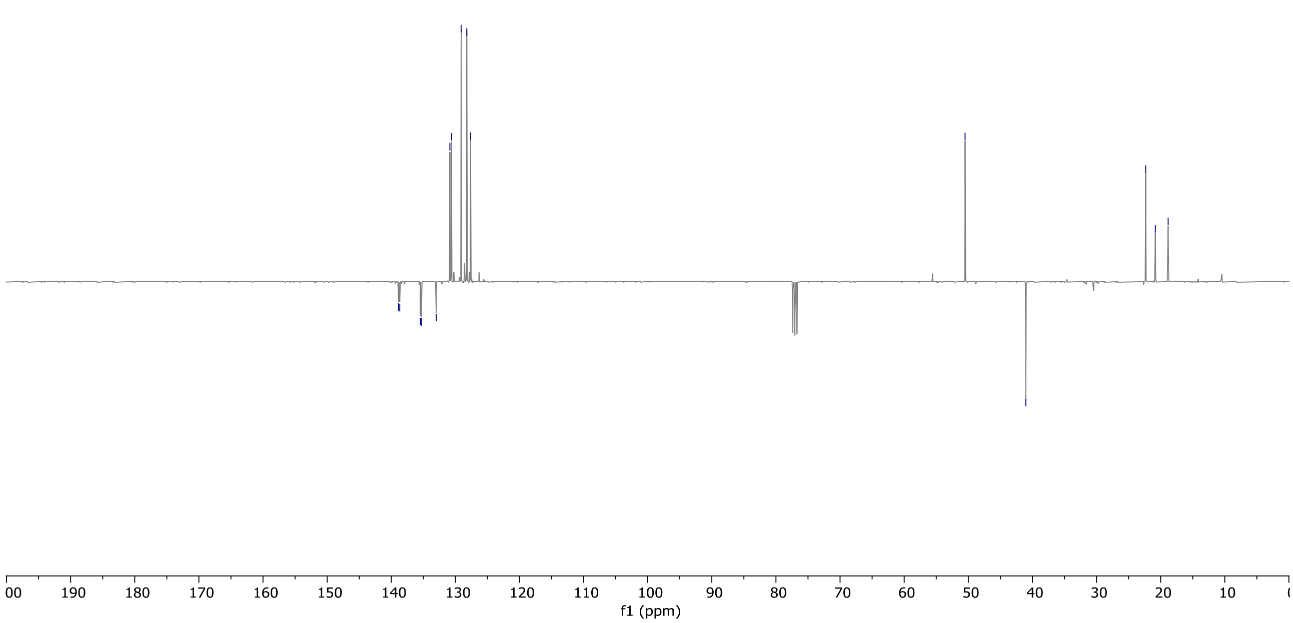

1-(2,4,6-Trimethoxyphenyl)-2-tosylamino-propane (6)

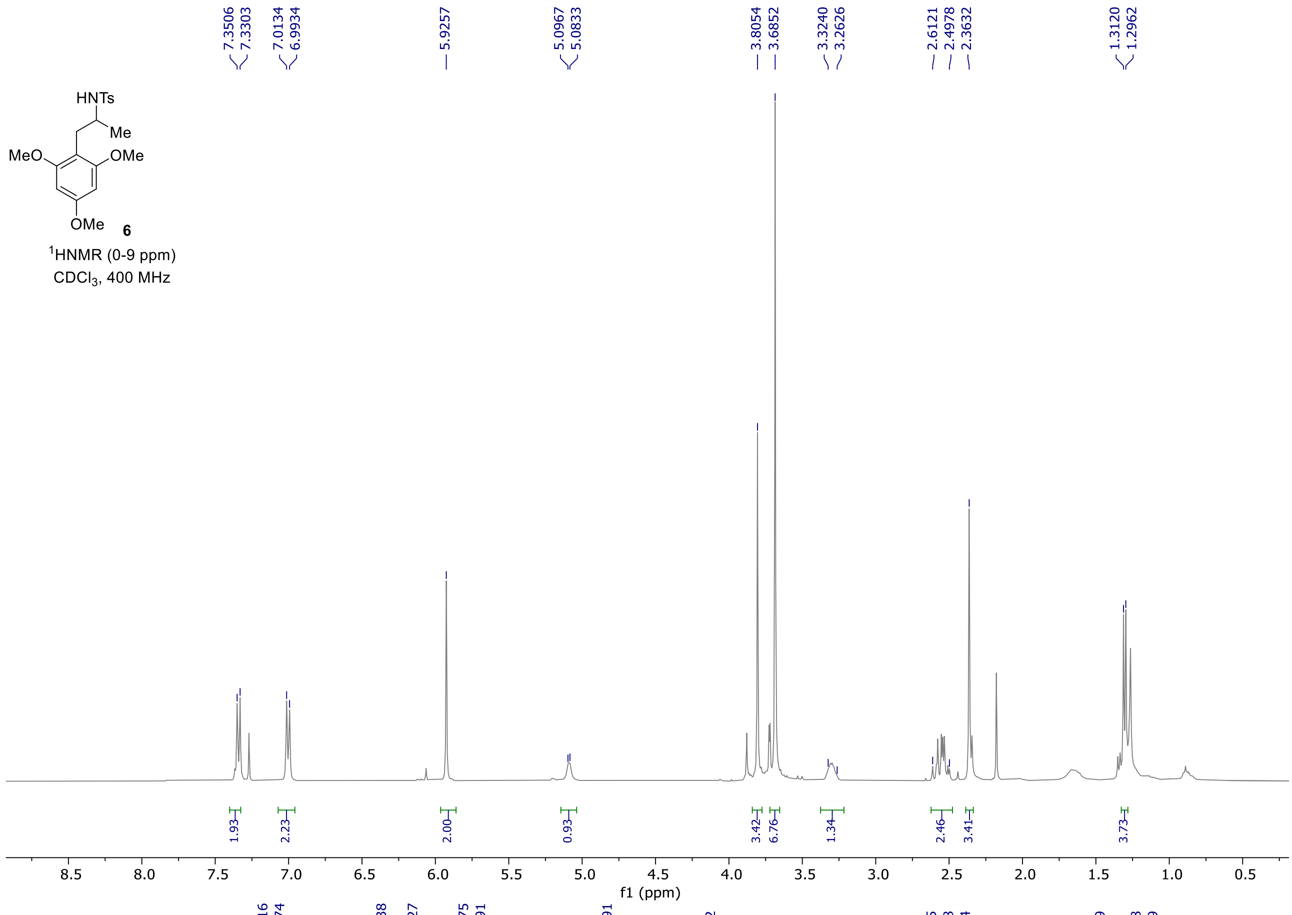

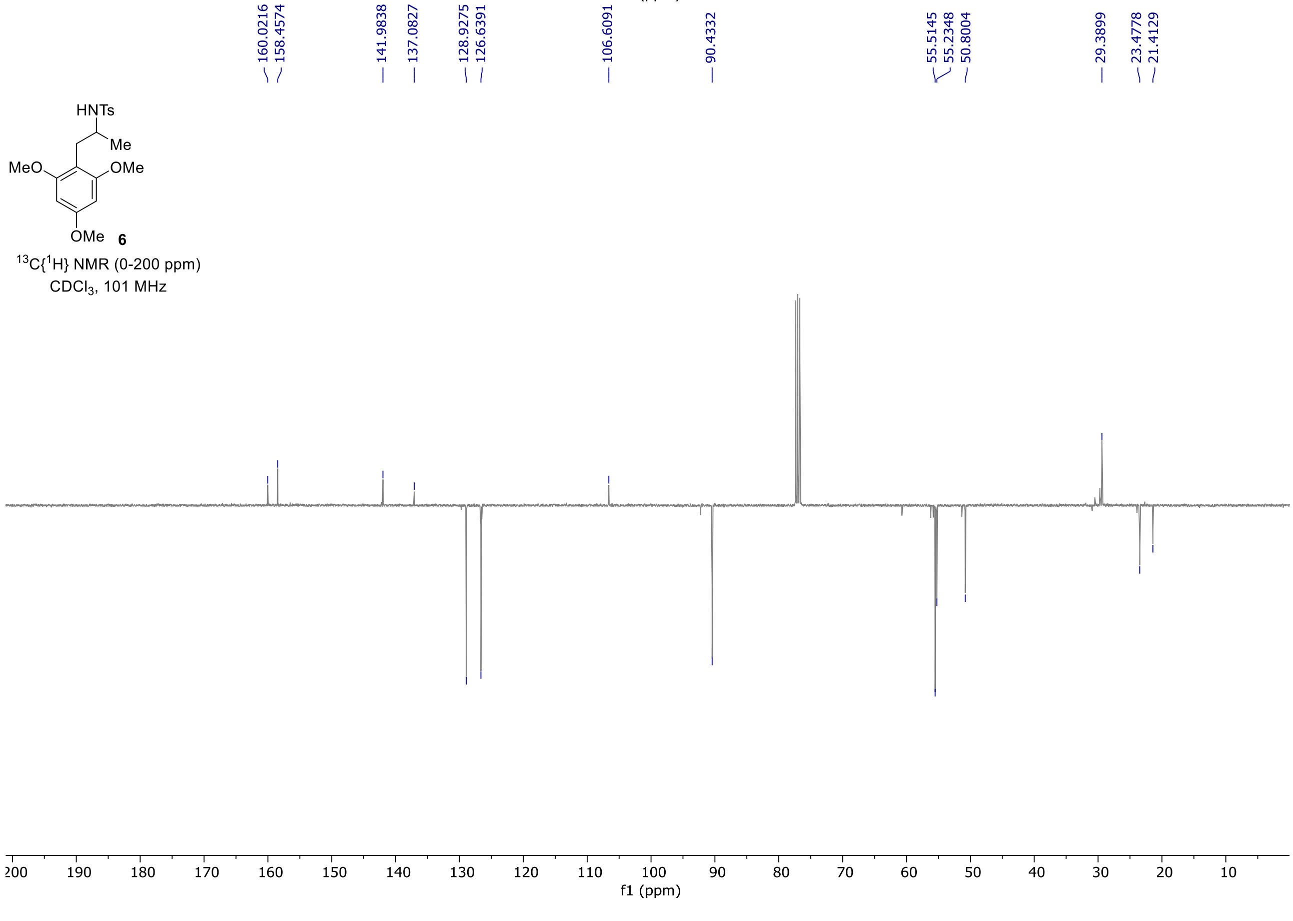

1-(2,5-Dimethoxyphenyl)-2-tosylamino-propane (7)

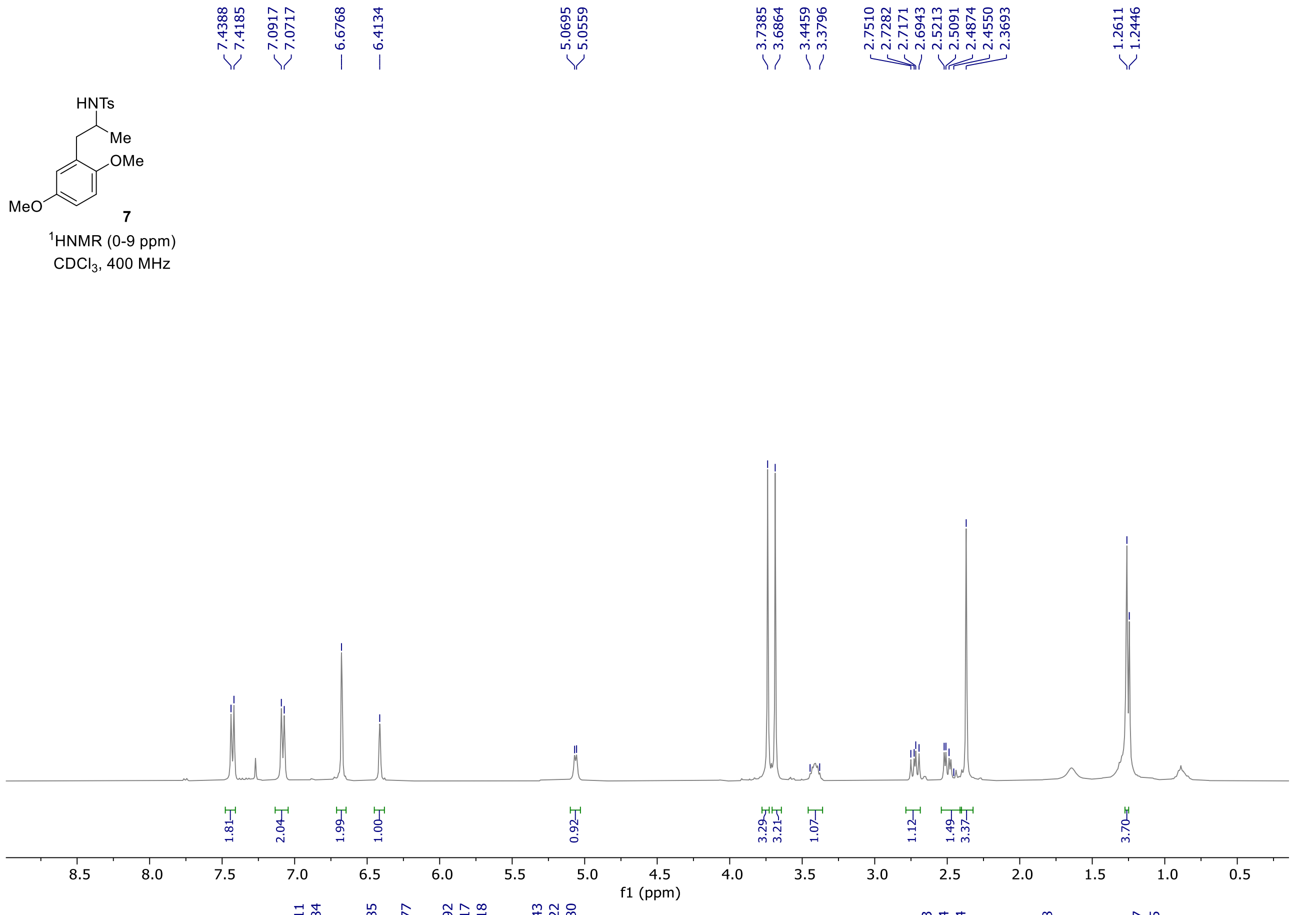

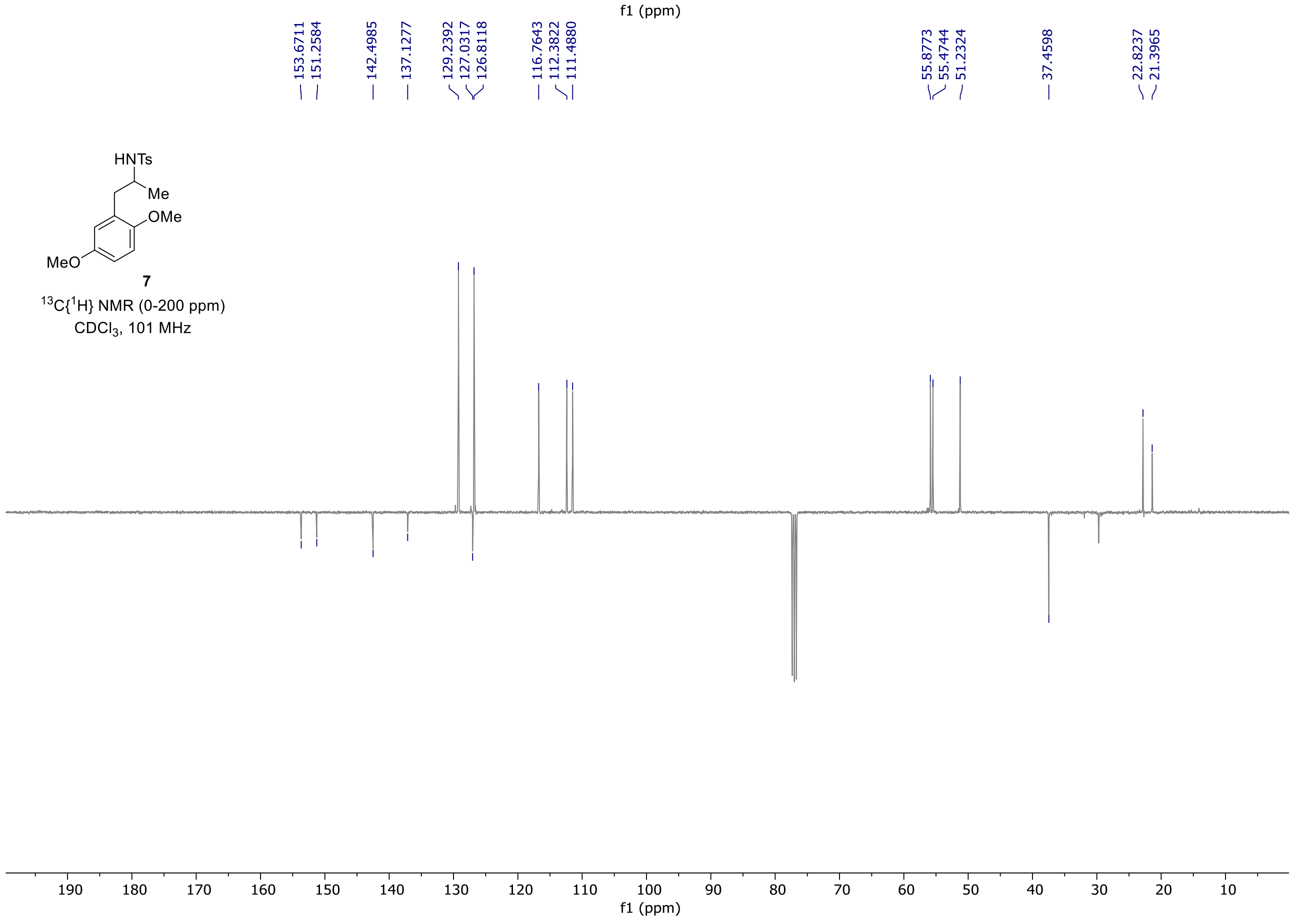

1-(5-Methylanisole)-2-tosylamino-propane (8)

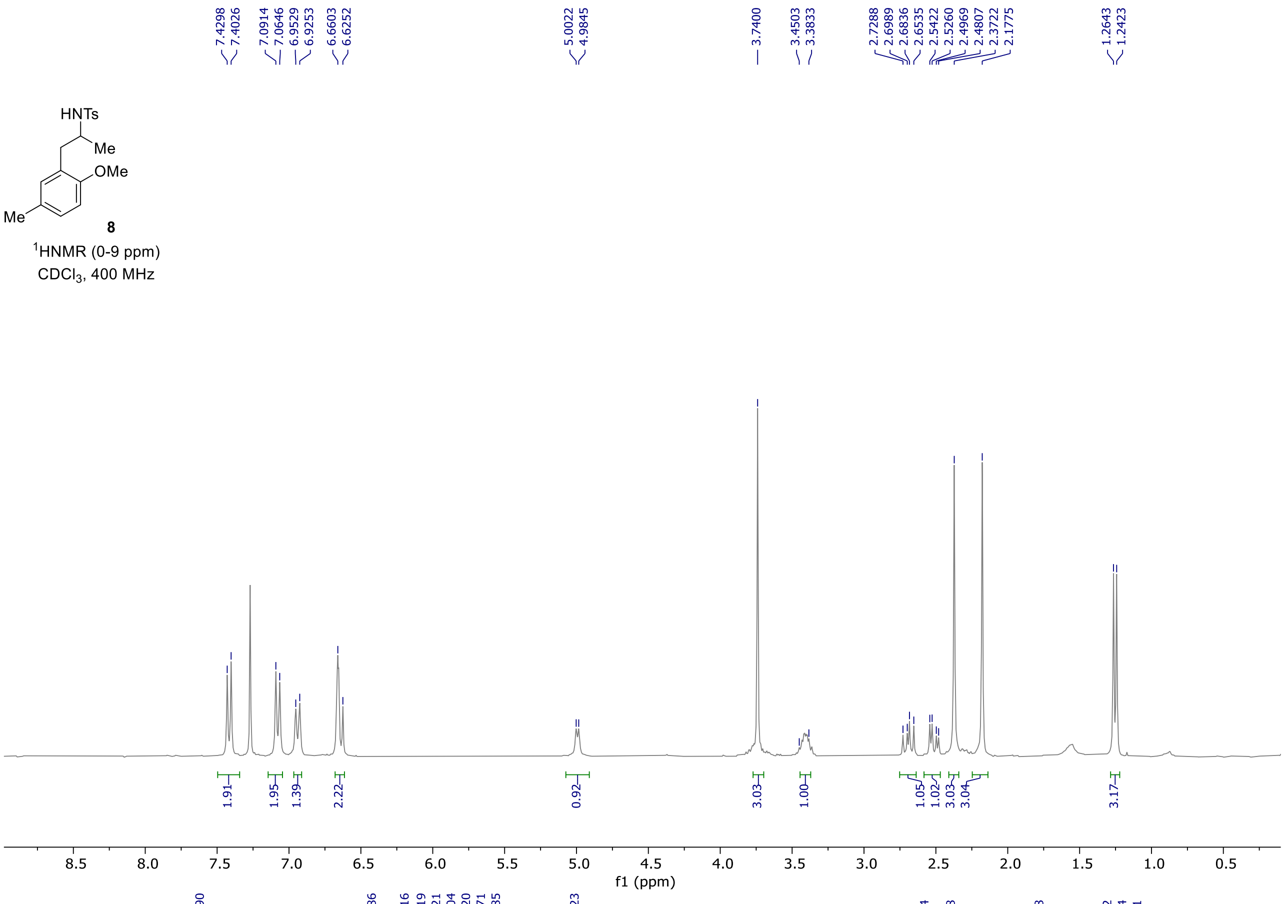

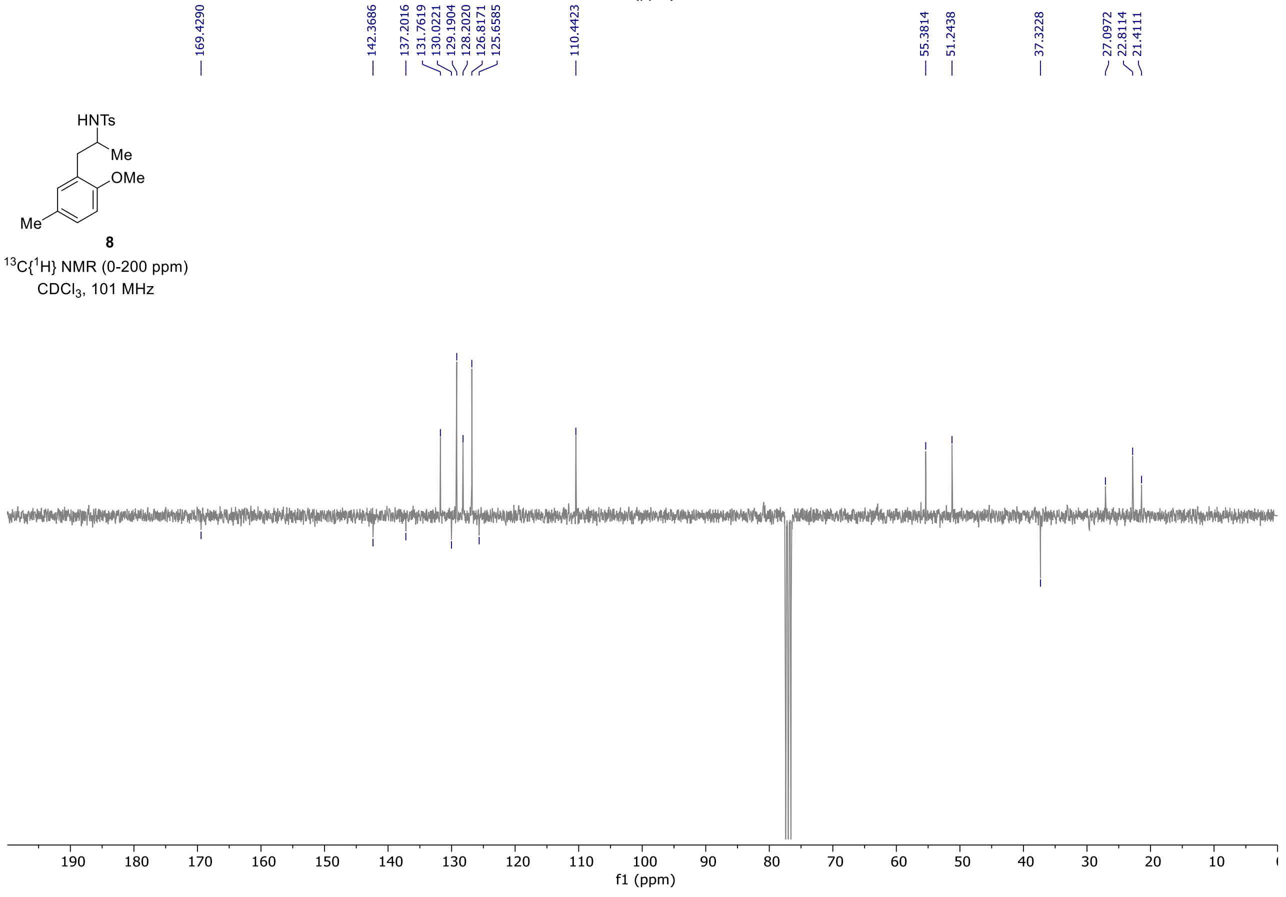

1-(5-Chloroanisole)-2-tosylamino-propane (9)

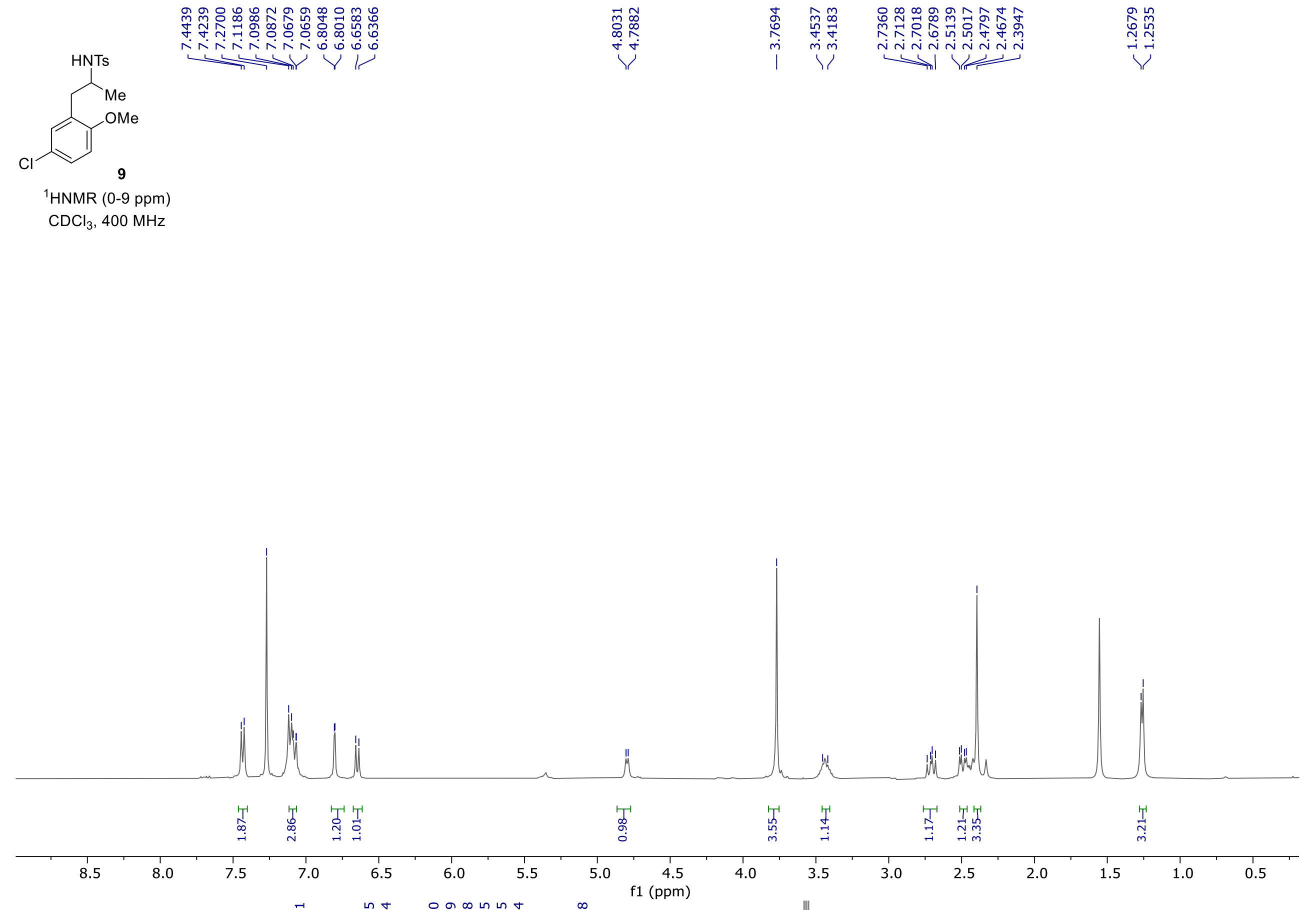

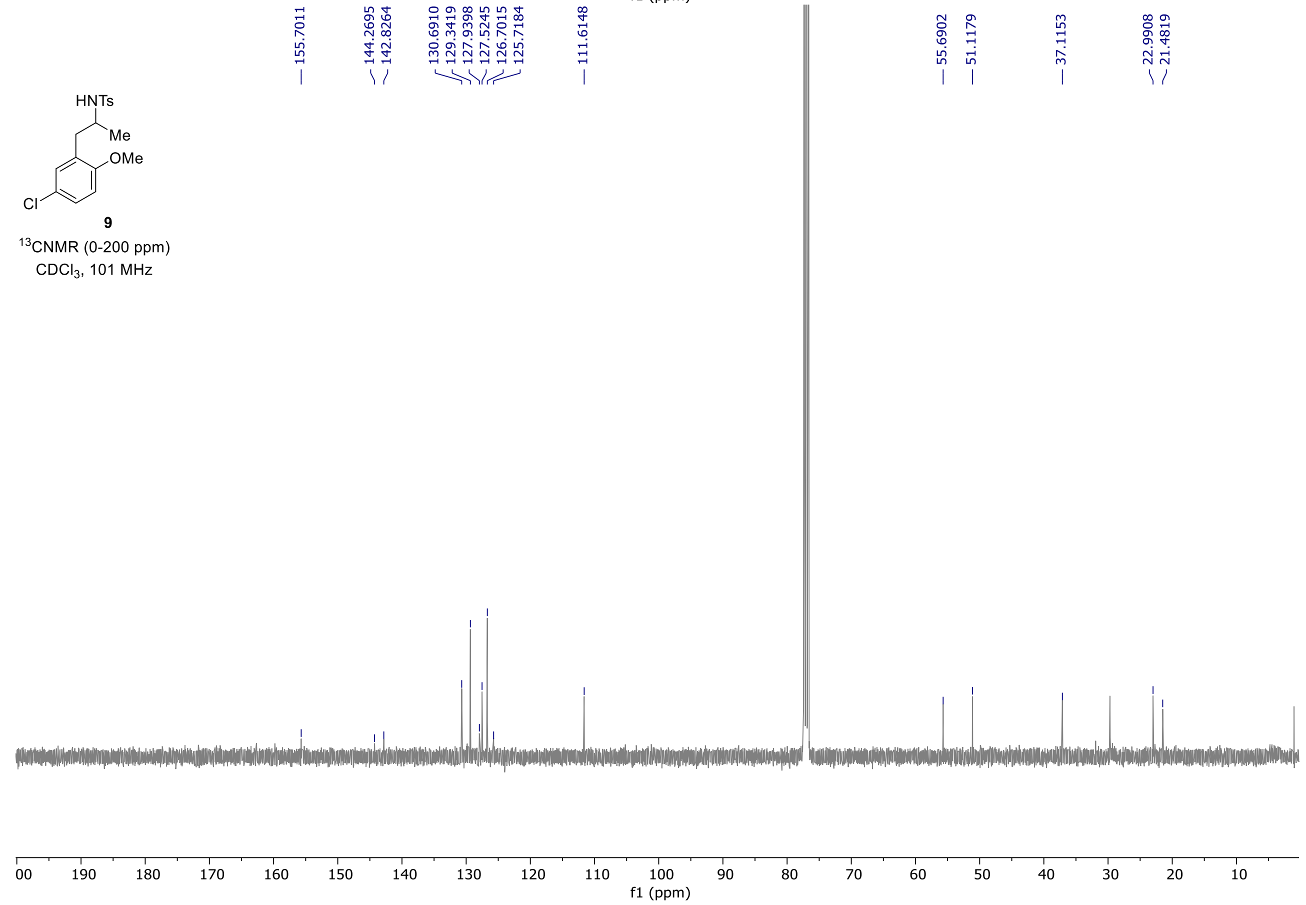

1-(5-Bromoanisole)-2-tosylamino-propane (10)

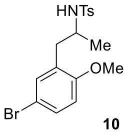

10

^{1}HNMR (0-9 ppm)
$CDCl_3$, 400 MHz

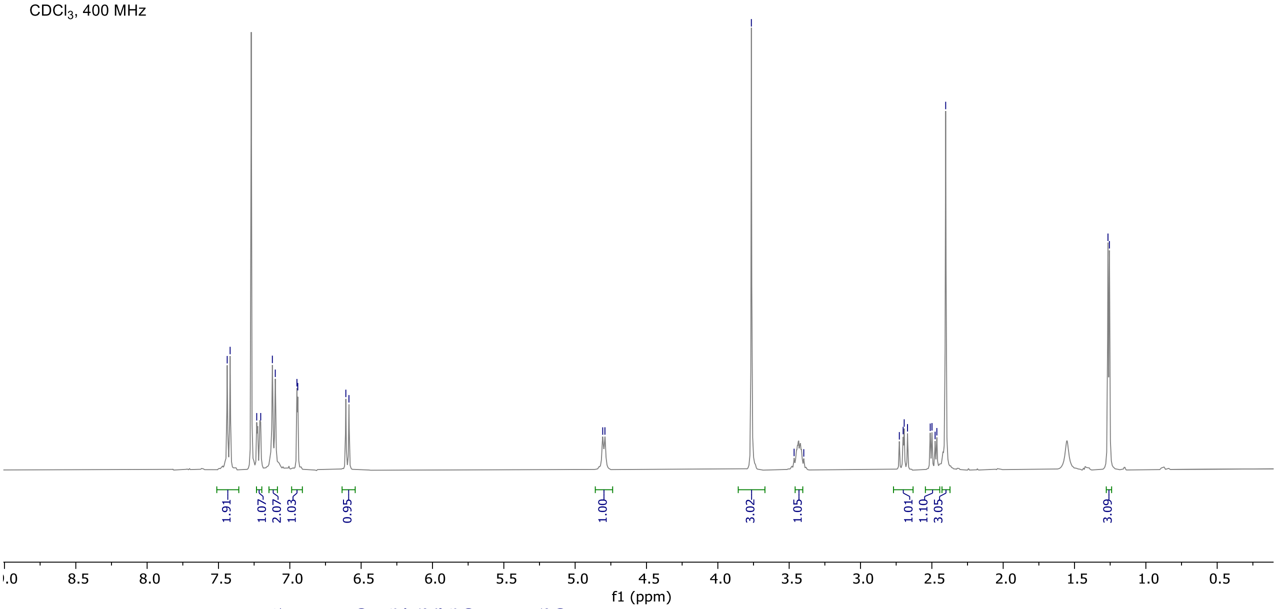

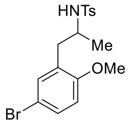

10

$^{13}C\{^{1}H\}$ NMR (0-200 ppm)
$CDCl_3$, 101 MHz

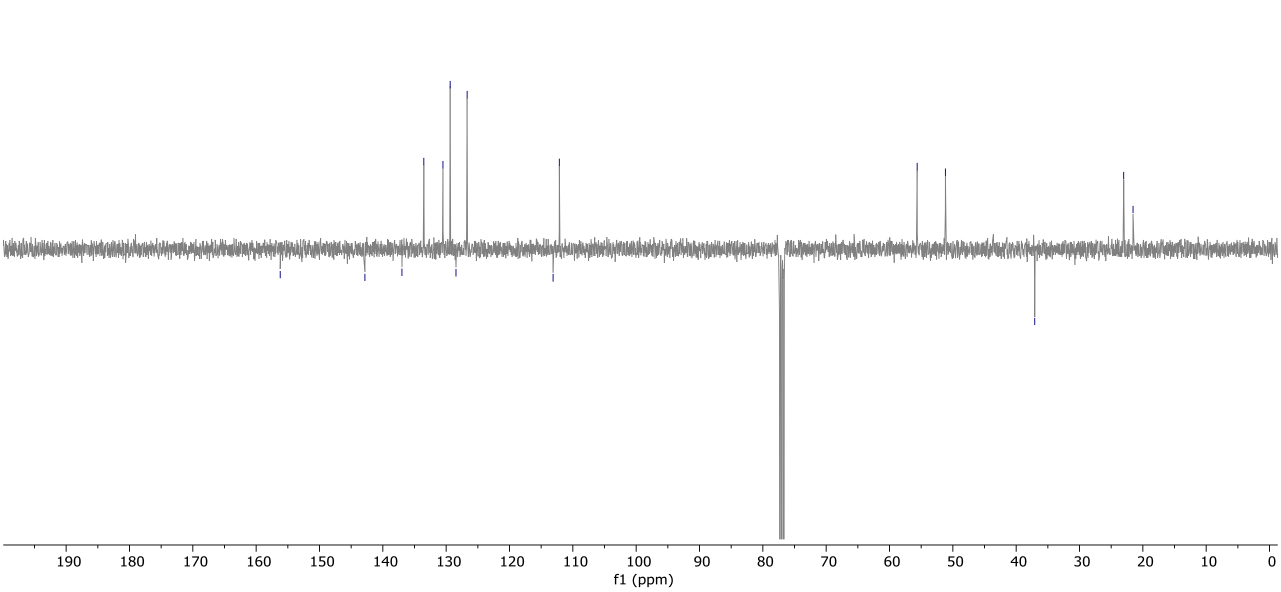

1-(5-Bromoanisole)-2-tosylamino-propane (11)

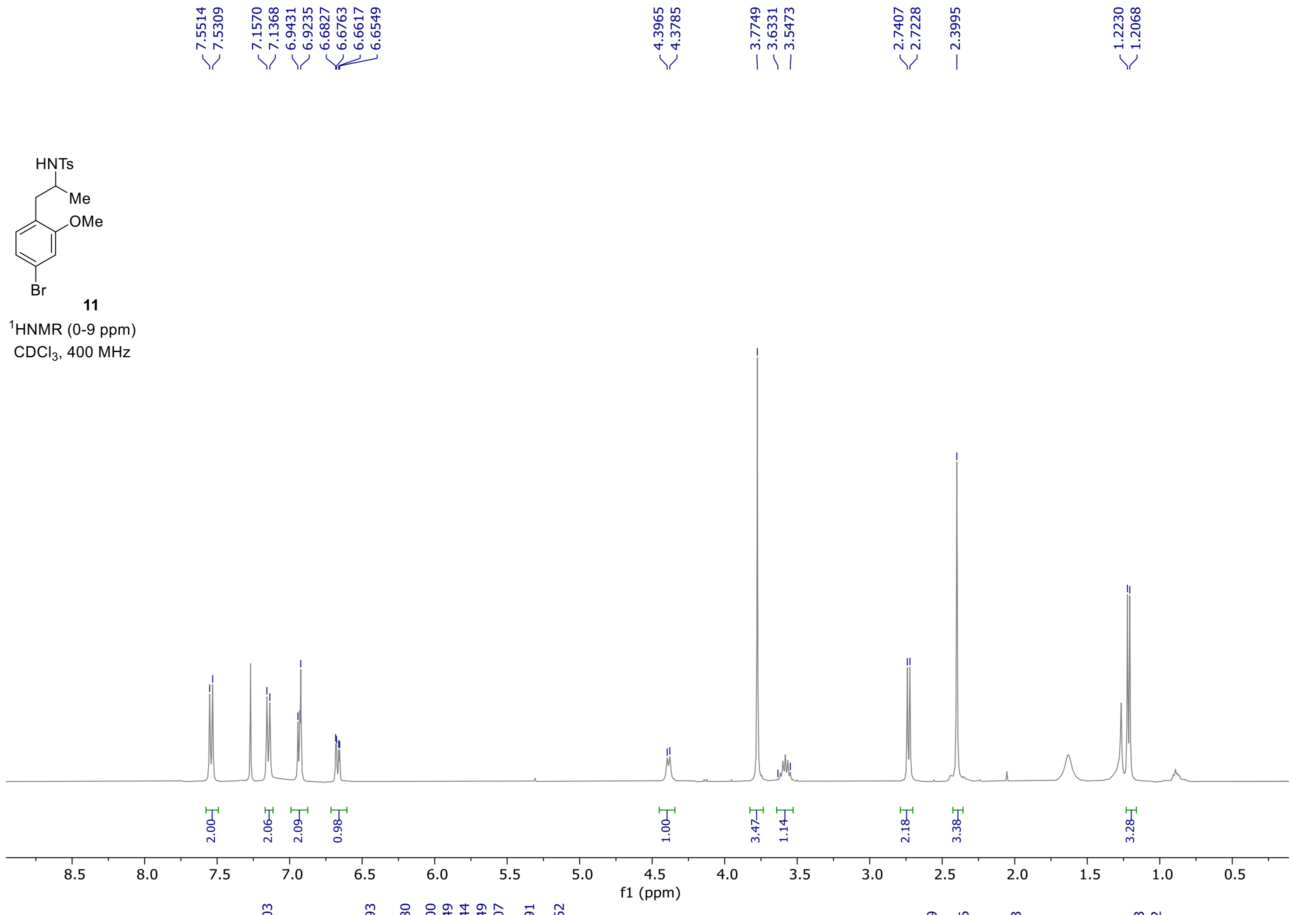

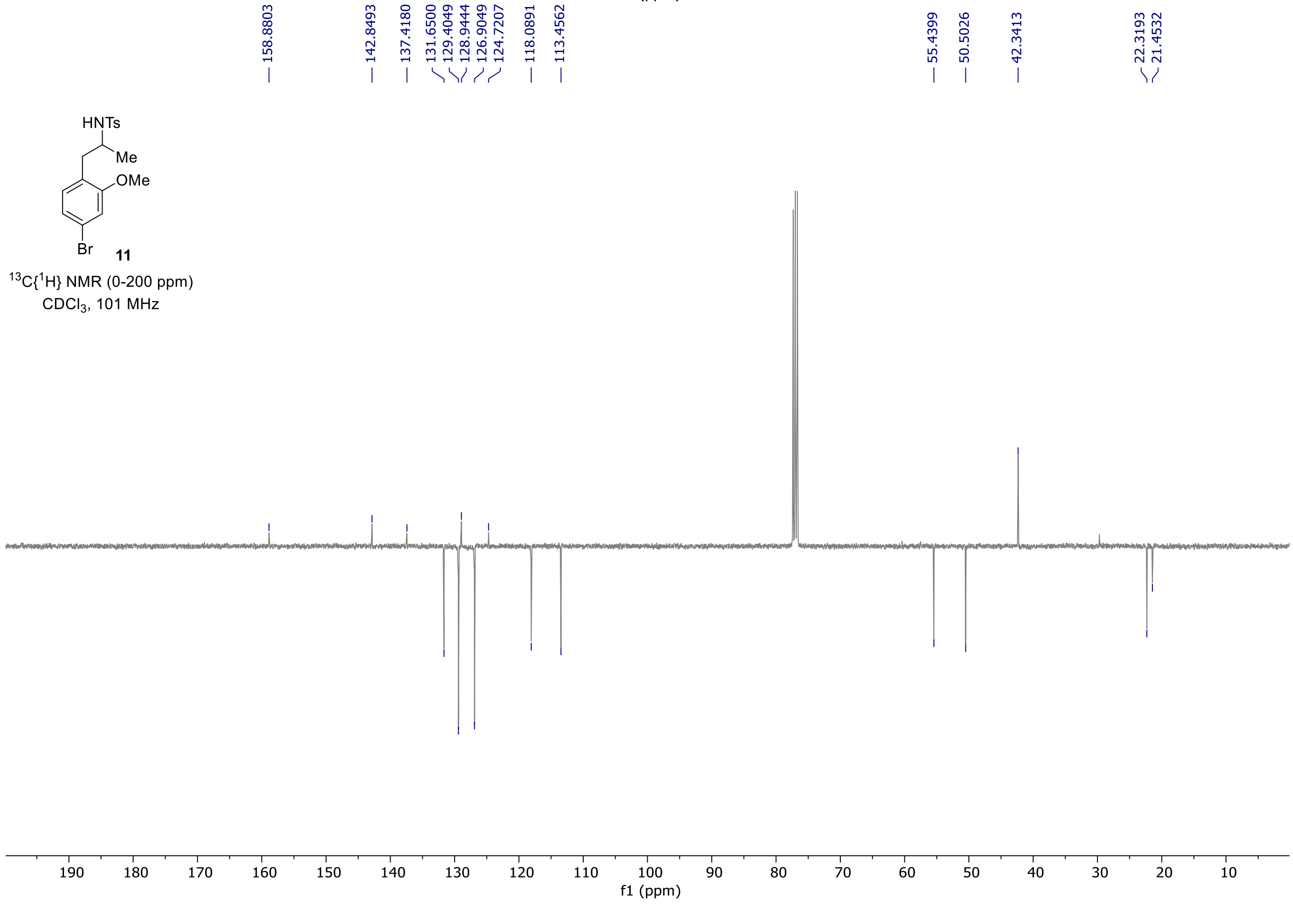

1-(2-Trimethoxyphenyl)- 2-tosylamino-propane and 1-(4-trimethoxyphenyl)-2-tosylamino-propane (12a + 12b)

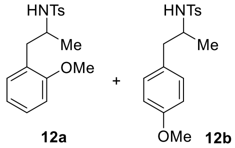

12a **12b**

^{1}HNMR (0-9 ppm)
$CDCl_3$, 400 MHz

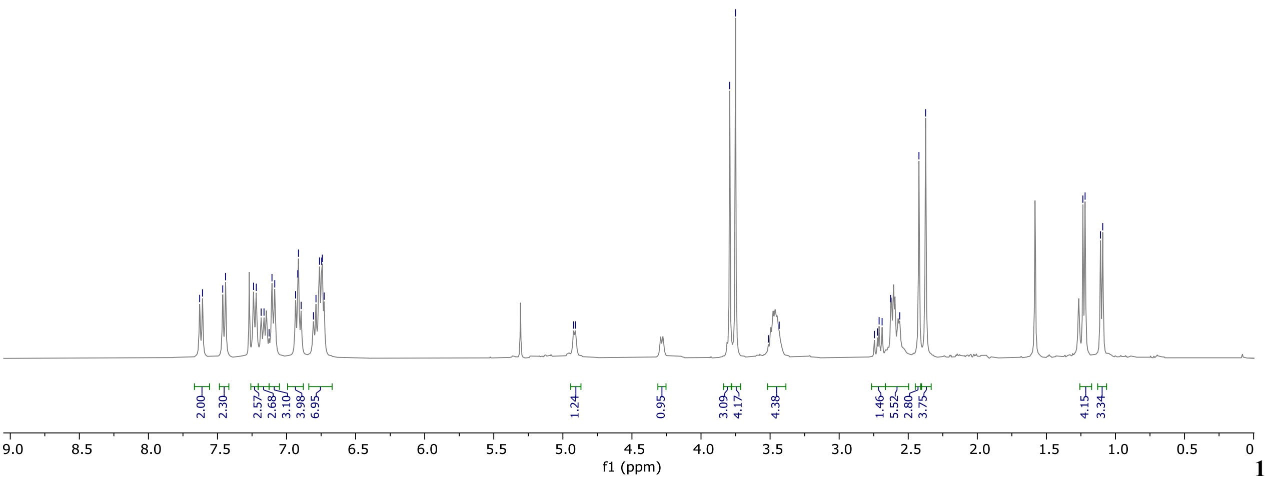

1-(3-Thienyl)-2-tosylamino-propane (13b)

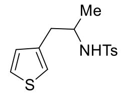

13b

^{1}HNMR (0-9 ppm)
$CDCl_3$, 400 MHz

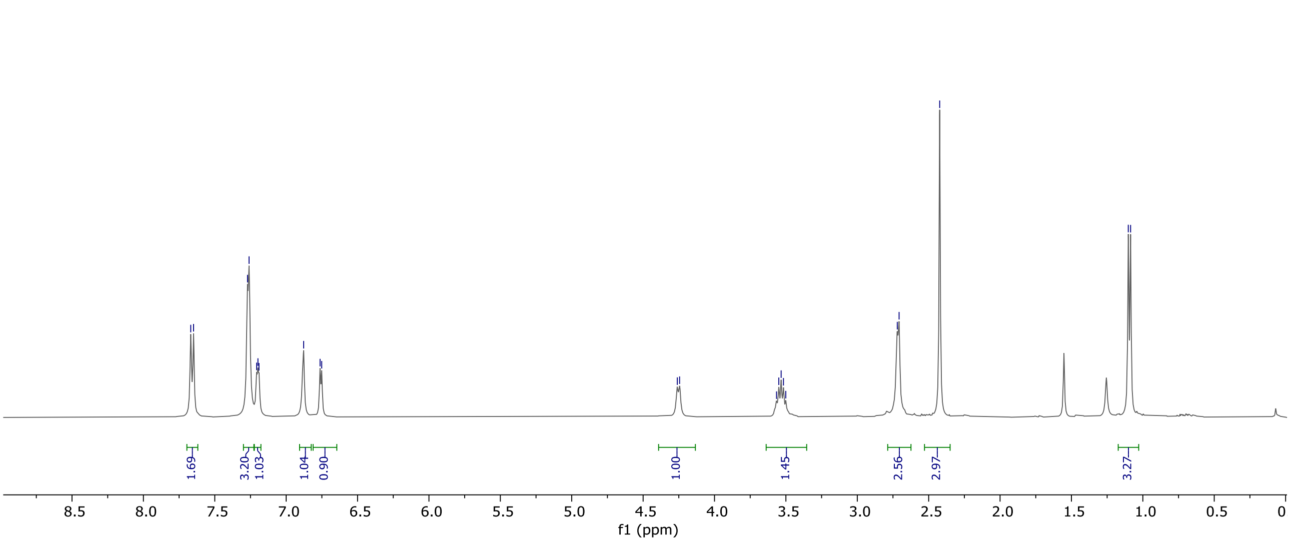

1-(2,4,6-Trimethylphenyl)-2-(benzenesulfonamido)-butane and 1-(2,4,6-trimethylphenyl)-3-(benzenesulfonamido)-butane (14a + 14b)

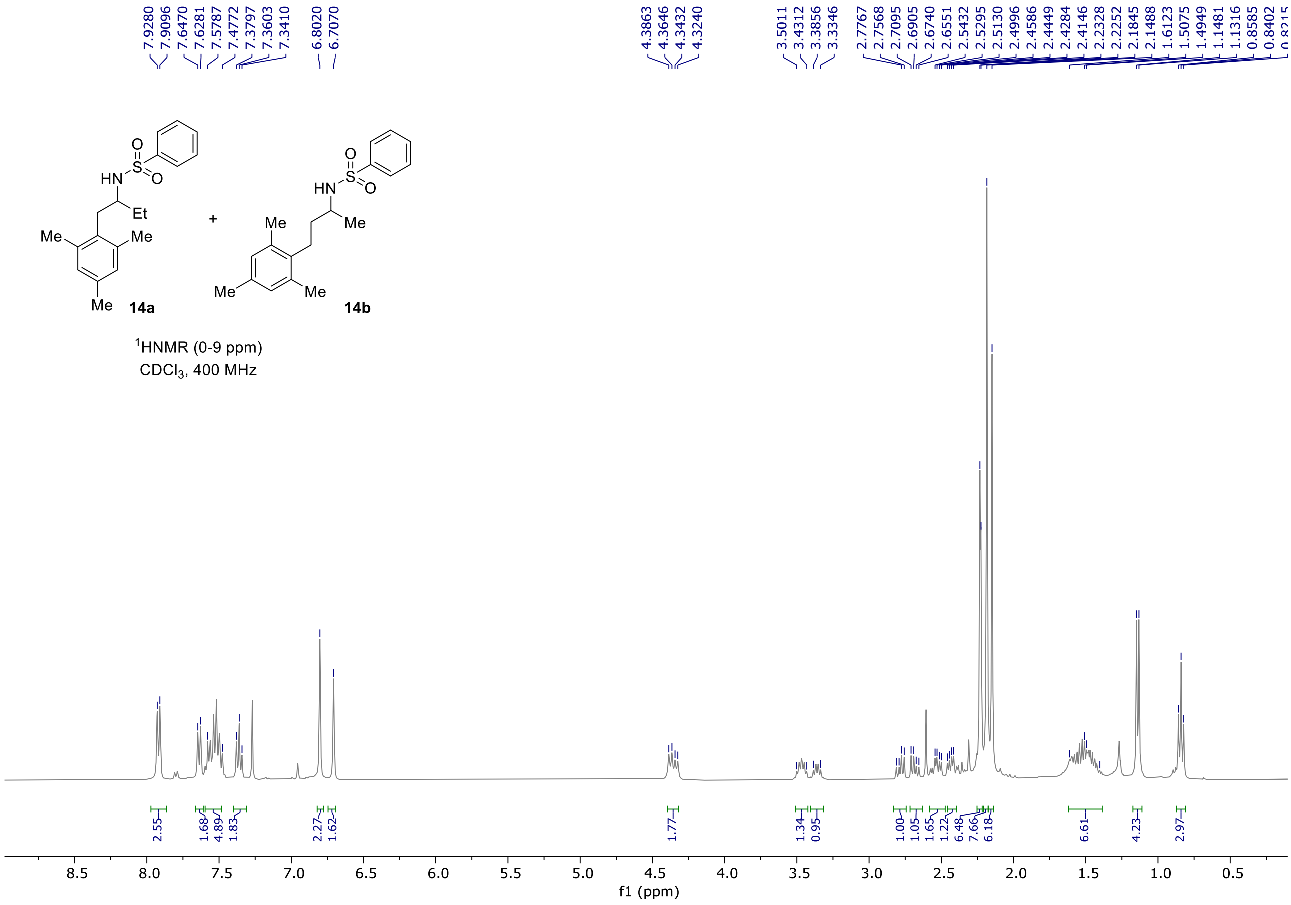

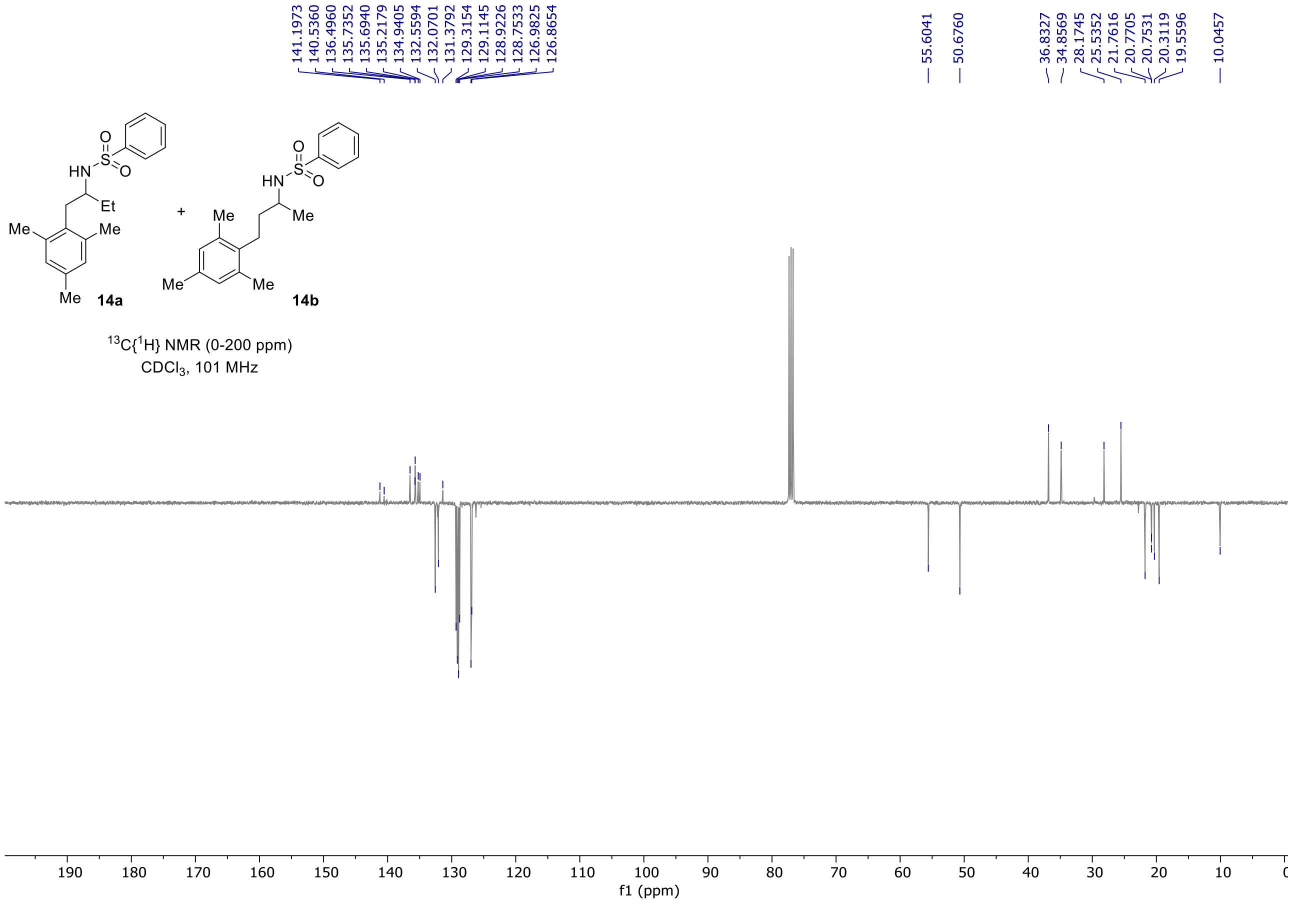

1-(2,4,6-Trimethylphenyl)-2-(4-chlorobenzenesulfonamido)-butane and 1-(2,4,6-trimethylphenyl)-3-(4-chlorobenzenesulfonamido)-butane (15a+ 15b)

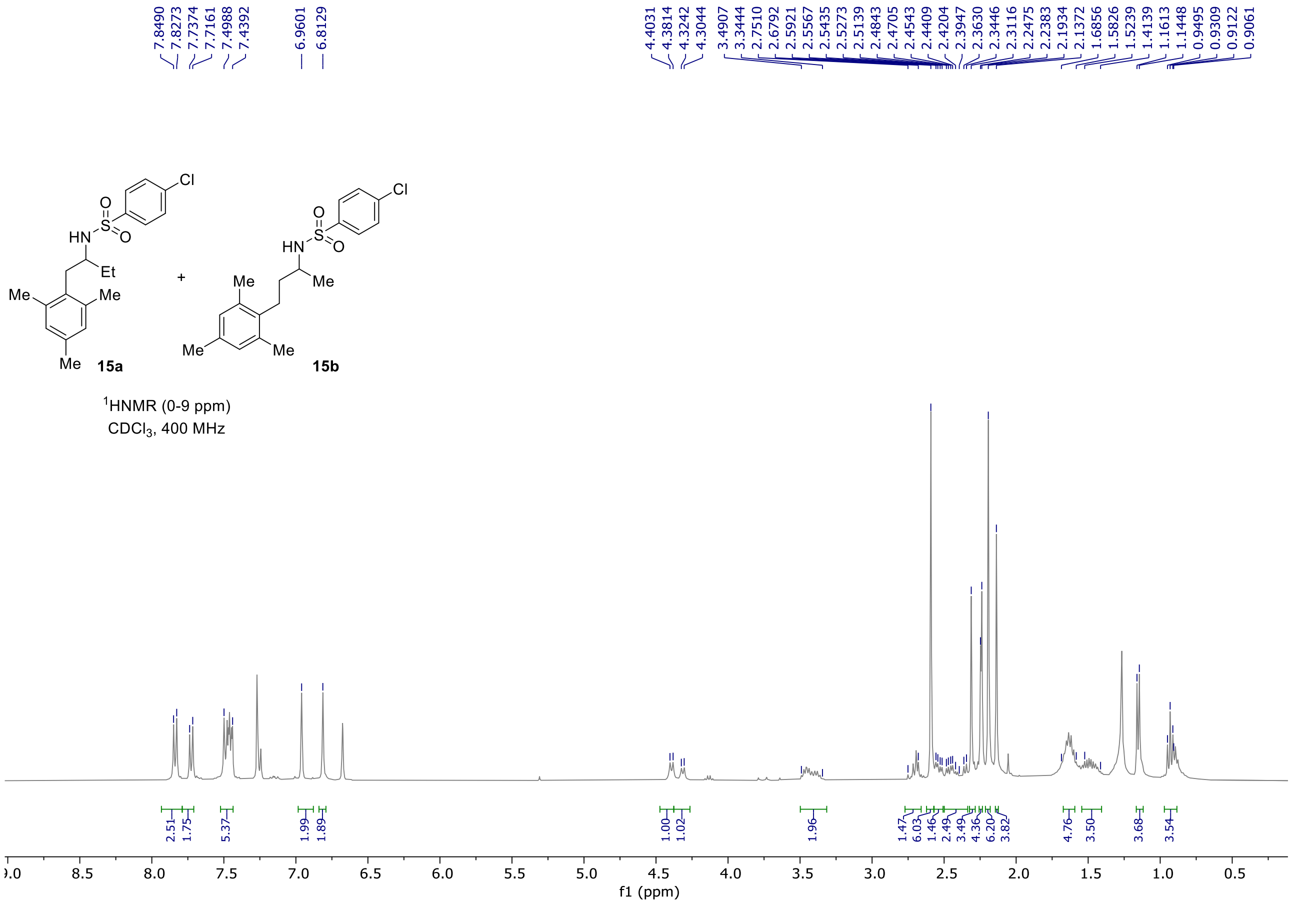

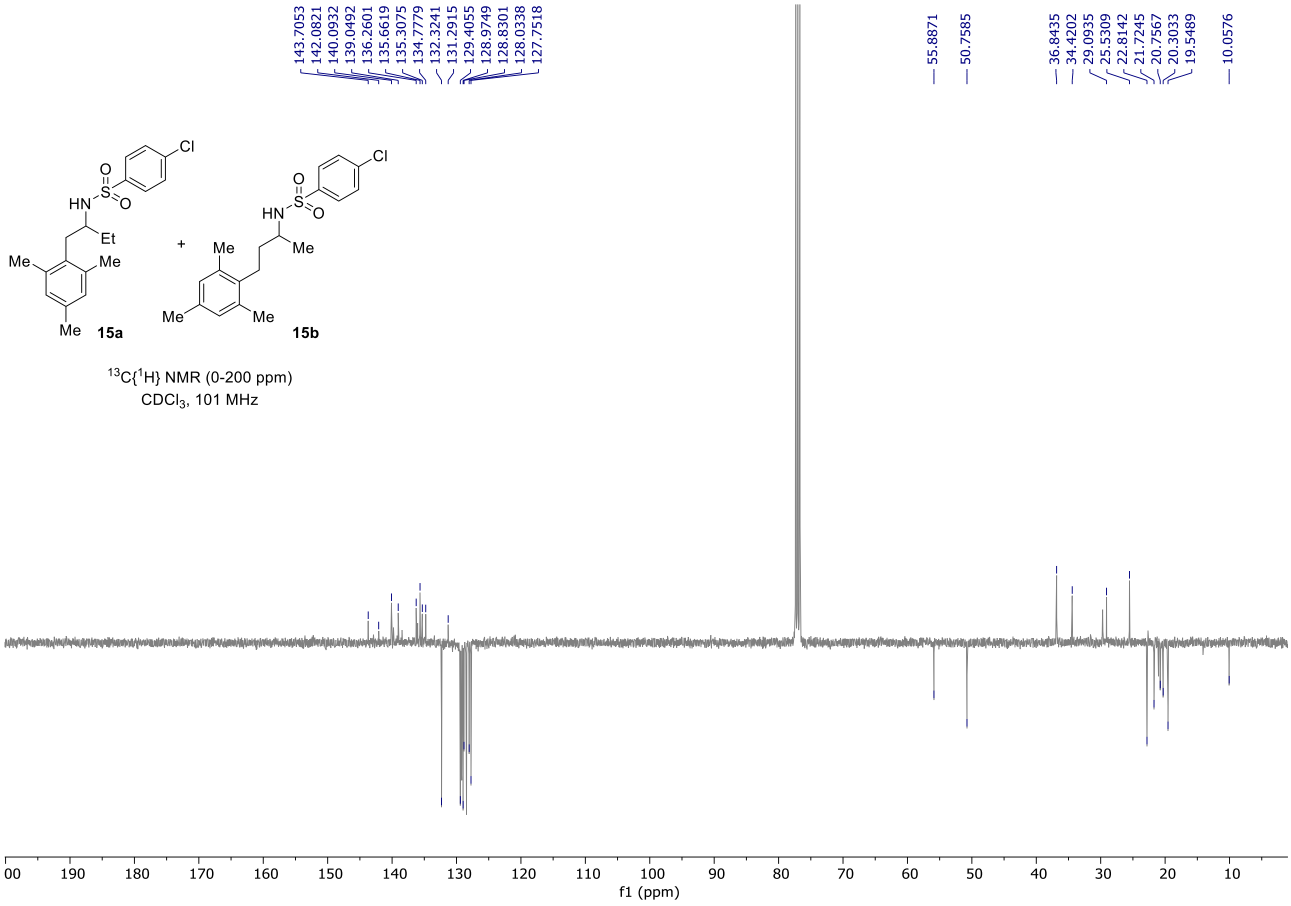

1-(2,4,6-Trimethylphenyl)-3-(4-chlorobenzenesulfonamido)-butane (15b)

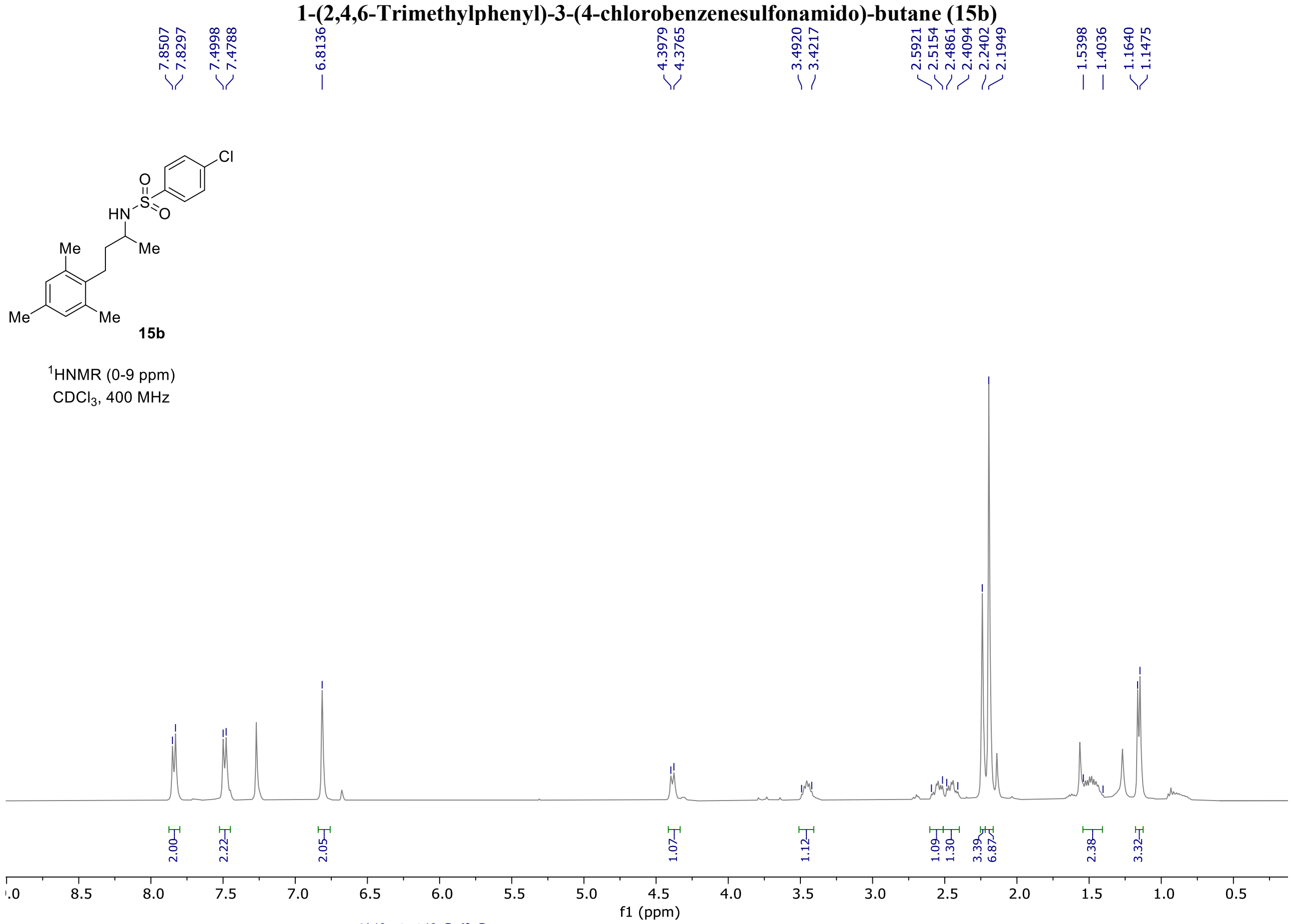

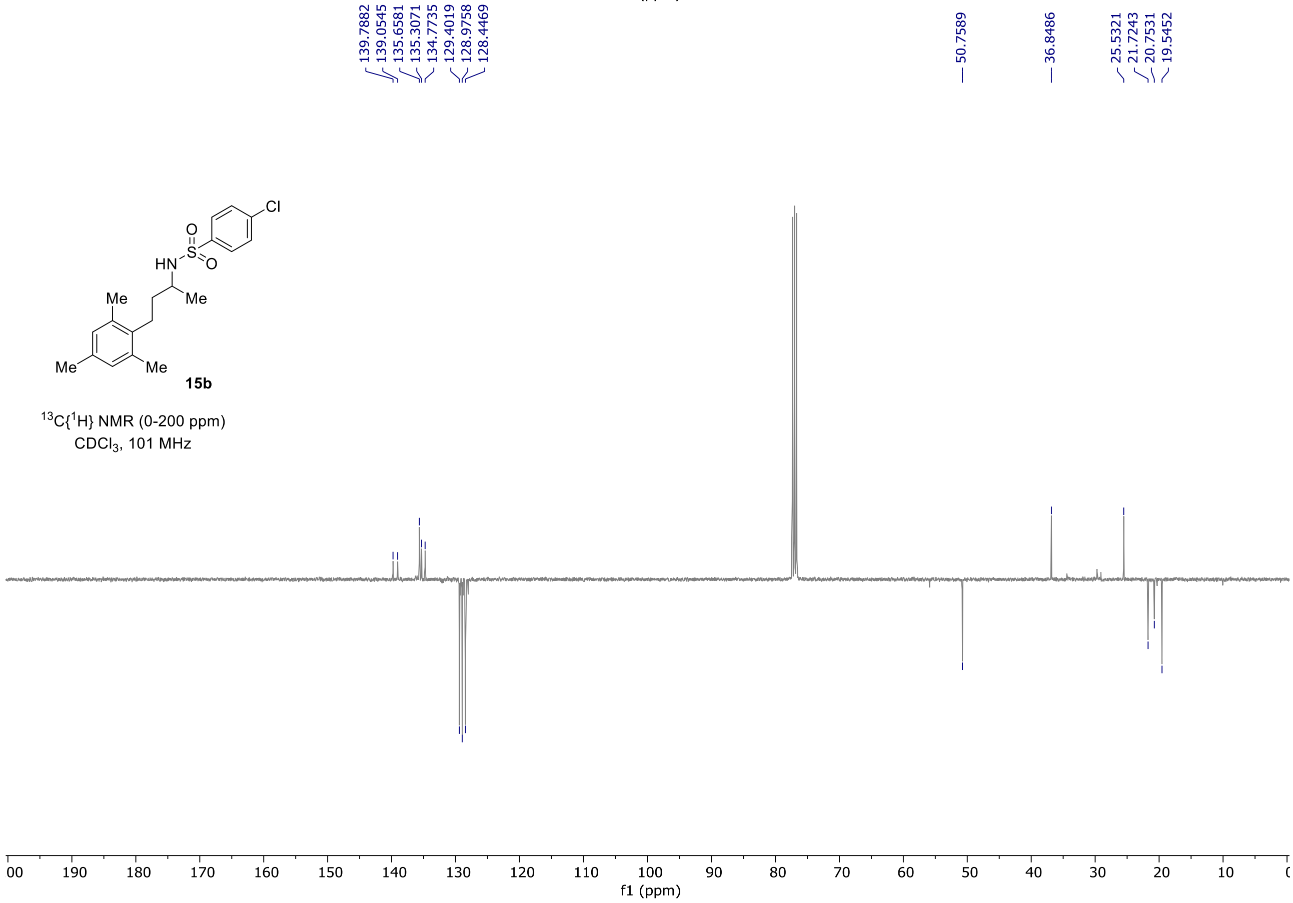

1-(2,4,6-Trimethylphenyl)-2-(*o*-nosylamino)-butane (16)

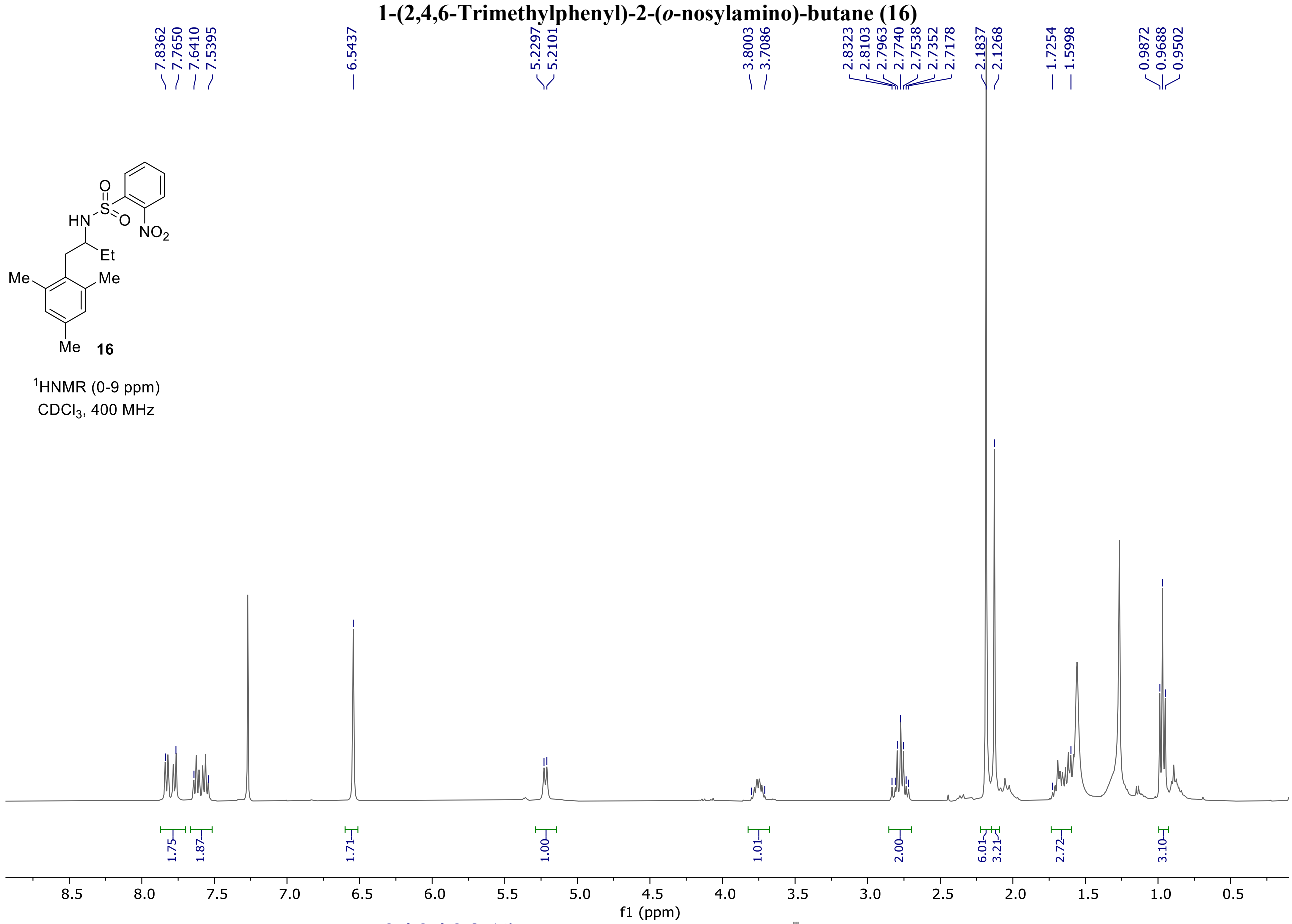

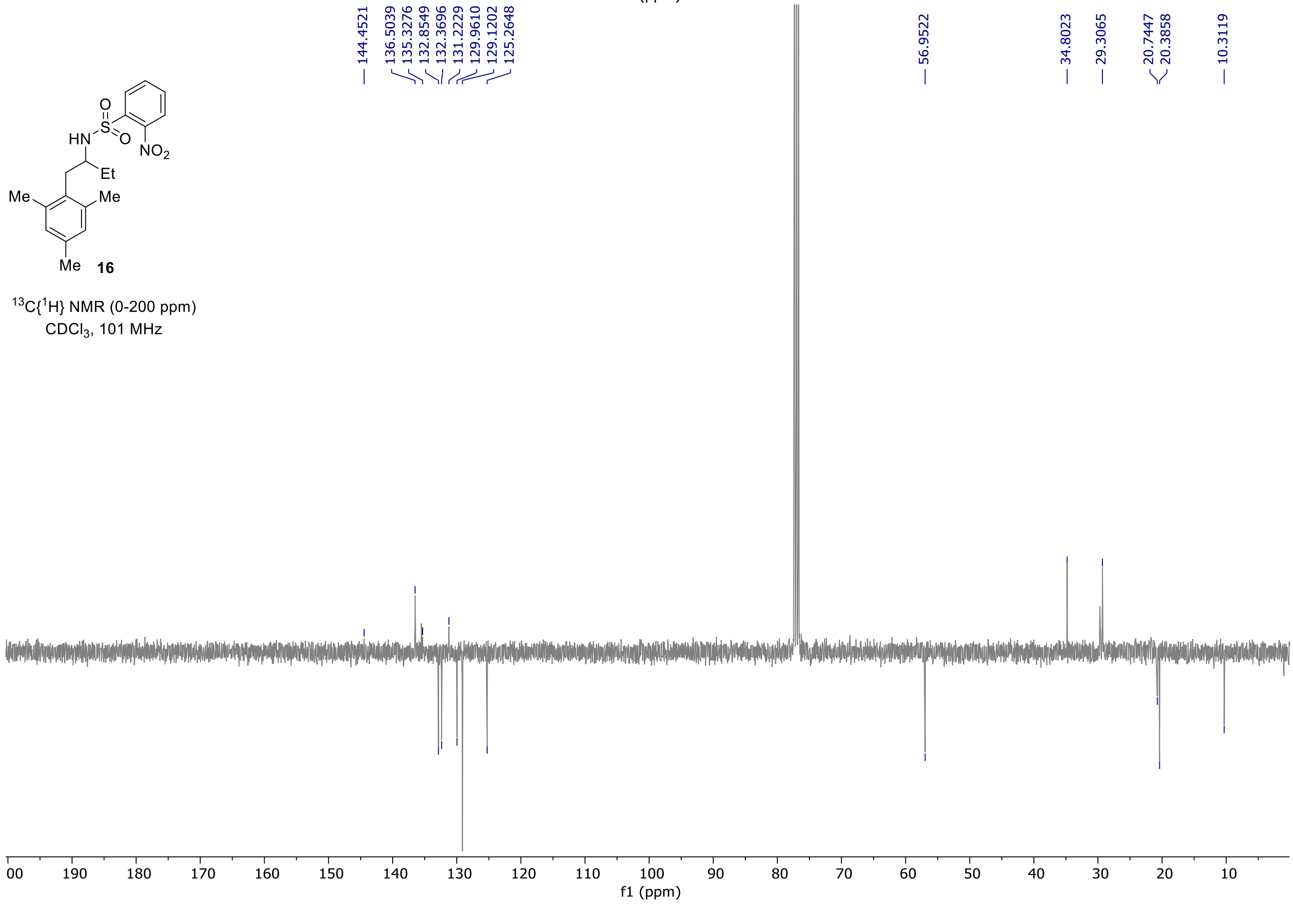

1-(5-1-(2,4,6-Trimethylphenyl)-2-(*p*-nosylamino)-butane (17)

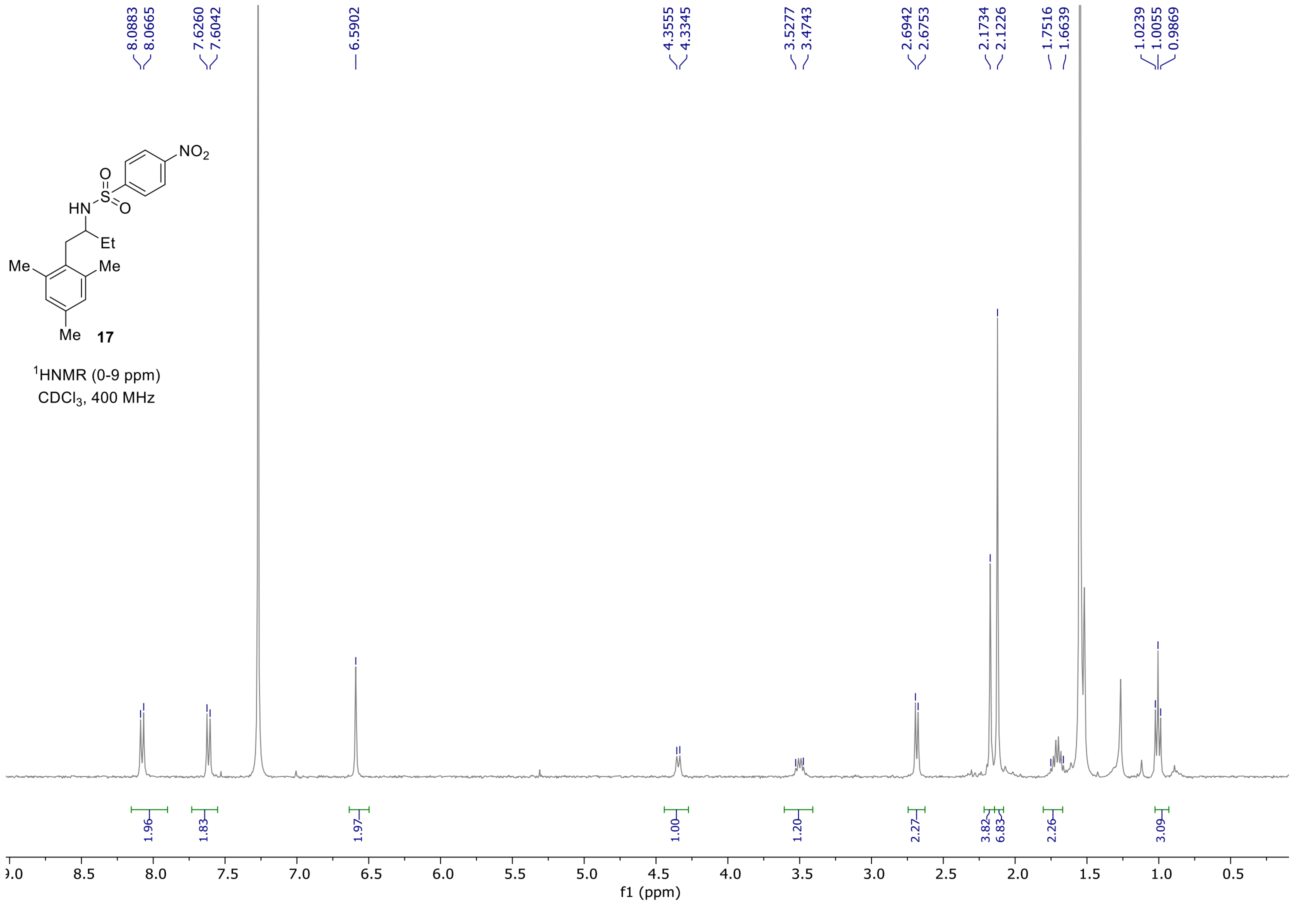

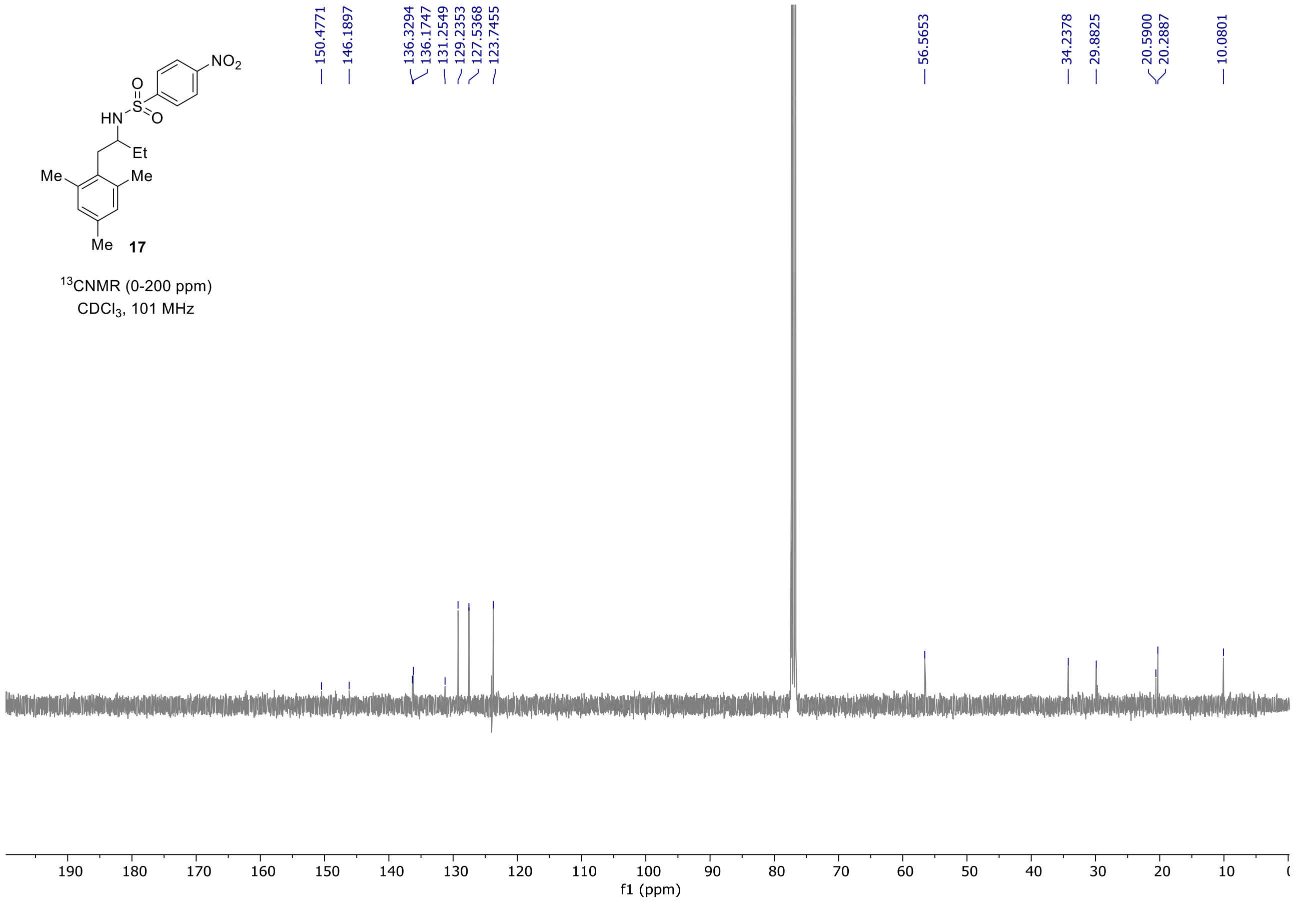

Supplement: Supplementary file 1 — jo3c01536_si_001.pdf [file jo3c01536_si_001.pdf]
